# Expression profiles of cell-wall related genes vary broadly between two common maize inbreds during stem development

**DOI:** 10.1186/s12864-019-6117-z

**Published:** 2019-10-29

**Authors:** Bryan W. Penning, Tânia M. Shiga, John F. Klimek, Philip J. SanMiguel, Jacob Shreve, Jyothi Thimmapuram, Robert W. Sykes, Mark F. Davis, Maureen C. McCann, Nicholas C. Carpita

**Affiliations:** 1 Department of Botany & Plant Pathology, Purdue University, 915 West State Street, West Lafayette, IN 47907 USA; 2 Department of Biological Sciences, Purdue University, 915 West State Street, West Lafayette, IN 47907 USA; 3 Present Address: USDA-ARS, Wheat Quality Research Unit, 1680 Madison Avenue, Wooster, OH 44691 USA; 4 Present Address: Departamento de Alimentos e Nutrição Experimental, FCF-USP F, 3091-3647 / 3091-3007, Av. Prof. Lineu Prestes, 580 - BL-14 CEP 05508-000, Butantã, Sâo Paulo, SP Brazil; 5 Genomics Core Facility, Purdue University, 170 South University Street, Purdue University, West Lafayette, IN 47907 USA; 6 Bioinformatics Core Facility, Purdue University, 155 South Grant Street, West Lafayette, IN 47907 USA; 7 Present Address: Department of Internal Medicine, Cleveland Clinic, 9500 Euclid Ave, Cleveland, OH 44195 USA; 8 National Bioenergy Center, National Renewable Energy Laboratory, Golden, CO 80401 USA; 9 Present Address: Los Alamos National Laboratory, P.O. Box 1663, Los Alamos, NM, Los Alamos, NM 87545 USA; 10 Purdue Center for Plant Biology, West Lafayette, USA

**Keywords:** *Zea mays* (maize), Stem development, Cell-wall biosynthesis, Gene expression, Transcript profiling, Lignocellulosic biomass

## Abstract

**Background:**

The cellular machinery for cell wall synthesis and metabolism is encoded by members of large multi-gene families. Maize is both a genetic model for grass species and a potential source of lignocellulosic biomass from crop residues. Genetic improvement of maize for its utility as a bioenergy feedstock depends on identification of the specific gene family members expressed during secondary wall development in stems.

**Results:**

High-throughput sequencing of transcripts expressed in developing rind tissues of stem internodes provided a comprehensive inventory of cell wall-related genes in maize (*Zea mays*, cultivar B73). Of 1239 of these genes, 854 were expressed among the internodes at ≥95 reads per 20 M, and 693 of them at ≥500 reads per 20 M. Grasses have cell wall compositions distinct from non-commelinid species; only one-quarter of maize cell wall-related genes expressed in stems were putatively orthologous with those of the eudicot Arabidopsis. Using a slope-metric algorithm, five distinct patterns for sub-sets of co-expressed genes were defined across a time course of stem development. For the subset of genes associated with secondary wall formation, fifteen sequence motifs were found in promoter regions. The same members of gene families were often expressed in two maize inbreds, B73 and Mo17, but levels of gene expression between them varied, with 30% of all genes exhibiting at least a 5-fold difference at any stage. Although presence-absence and copy-number variation might account for much of these differences, fold-changes of expression of a *CADa* and a *FLA11* gene were attributed to polymorphisms in promoter response elements.

**Conclusions:**

Large genetic variation in maize as a species precludes the extrapolation of cell wall-related gene expression networks even from one common inbred line to another. Elucidation of genotype-specific expression patterns and their regulatory controls will be needed for association panels of inbreds and landraces to fully exploit genetic variation in maize and other bioenergy grass species.

## Background

The disassembly of lignocellulosic biomass to release sugars and aromatics, as substrates for fuels and chemicals, could be enhanced by the ability to modulate both the composition and the interactions of the polymers of cell walls [1]. The component sugars and aromatics exist in complex polymers that interact to form higher-order architectures that differ by cell type and species. Various grass species, including maize, are potential bioenergy crops but recalcitrance, the intrinsic resistance of cell walls to disassembly, needs to be overcome. The primary walls of grass species contain a network of phenylpropanoids, one of several features that distinguishes them from the primary walls of dicot and non-commelinid monocot species [2]. Secondary walls are thickened and lignified in specific cell types that contribute to substantial amounts of biomass. Genome-wide transcript-profiling technologies have been used to identify suites of genes involved in deposition of thickened and lignified secondary walls in Arabidopsis and poplar [3–5] and in the synthesis and assembly of grass-specific wall components abundant in C4 grass species [6, 7].

The cellular machinery for cell wall synthesis and metabolism is encoded by members of large multi-gene families and comprises an estimated 10% of plant genes [8]. All plant genomes sequenced thus far have cell wall-related genes represented in the same gene families. However, maize family subgroup structure reflects genome duplication events in grass species, and neo- and sub-functionalization associated with synthesis of walls specific to cell type or developmental stage, or in response to biotic or abiotic stimuli [9]. Comparison of grass gene families to those of Arabidopsis revealed variations between grass and dicot that parallel compositional differences and abundances of their respective phenylpropanoid, glucuronoarabinoxylan (GAX), xyloglucan (XyG), and pectin constituents [9]. To gain genetic control of maize secondary wall architecture, we need to identify regulatory networks and the specific gene family members expressed in stems.

Here, we used high-throughput RNA sequencing (RNA-seq) to identify genes expressed in rind tissues of stem internodes during secondary wall development in maize (*Zea mays* cv. B73). Of 1239 cell wall-related maize B73 genes, 854 at ≥95 reads per 20 M reads were expressed in one or more of seven internodes that represented five developmental stages from elongation and primary wall synthesis to secondary wall formation. Establishing gene expression networks for maize is complicated by large genetic variation within the species [10, 11]. Previously, we found significant transgressive segregation in an Intermated B73 x Mo17 population that established quantitative trait loci for lignin abundance and enzyme digestibility of stem walls, and even broader phenotypic variance in a collection of maize genotypes capturing 80% of species diversity [12].

Paschold et al. [13] found genome-wide differences in gene expression between B73 and Mo17 cultivars in primary root tissues. We also found expression differences between the B73 and Mo17 of 5-fold or greater for at least 30% of all genes, genome-wide, during all stages of stem development. For secondary wall-related genes, a set of fifteen motifs were represented in promoter regions that are potential regulatory elements. Future strategies for genetic improvement of maize and other grasses as bioenergy crops will need to account for genotypic differences in expression networks of cell wall-related genes that give rise to walls of similar composition and architecture.

## Results

### Cellulose, xylan, and lignin contents increase in maize rind tissue during internode development

**Fig. 1:**
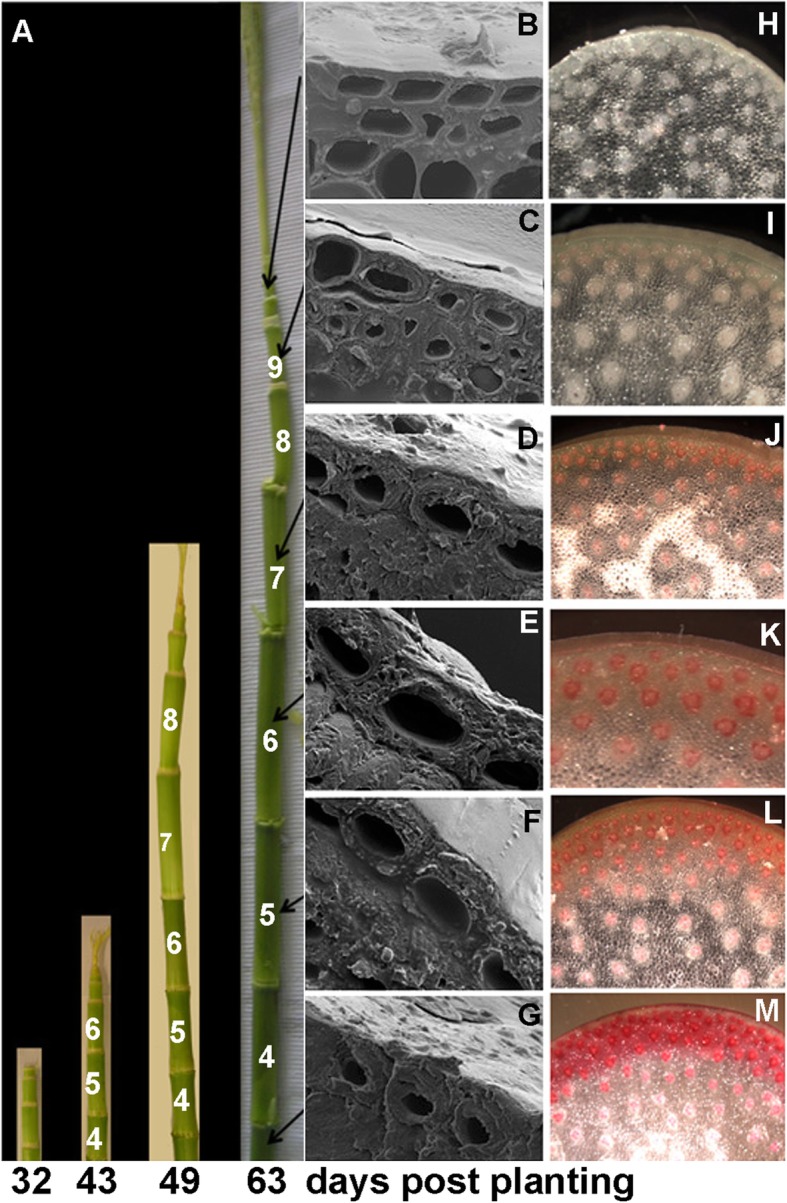
Cell wall thickness and lignin content increase in rind tissues of maize internodes with developmental age. **a** Maize stems at 35, 42, 49, and 63 days after planting add new internodes at their apex and elongate over time. Scale bar, 10 cm. **b-g** Scanning electron micrographs show cell walls of rind tissue from internodes nearer the apex of the maize stem have thinner cell walls compared to internodes closer to the base. Scale bar, 10 μm. **h-m:** Phloroglucinol staining intensity increases from faint pink to dark red in stem sections from the apex to base of the maize stem indicating increasing lignin content towards the base. Scale bar, 1 mm

Maize stem development began at the fifth-leaf stage and culminated with tassel formation after five weeks. Stem elongation began in basal internodes and proceeded sequentially with those closer to the apex elongating later (Fig. 1a). Wall thickening of the rind epidermis and sclerenchyma (Fig. 1b-g) and their subsequent lignification as indicated by phloroglucinol staining of transverse sections (Fig. 1h-m), occurred first in basal internodes and progressed in a gradient towards the apex (Fig. 1, a-m). In greenhouse-grown plants sampled at 49-d after planting, internodes 6 and 7 were maximally elongating and older internodes 4 and 5 deposited lignifying secondary walls.

**Fig. 2:**
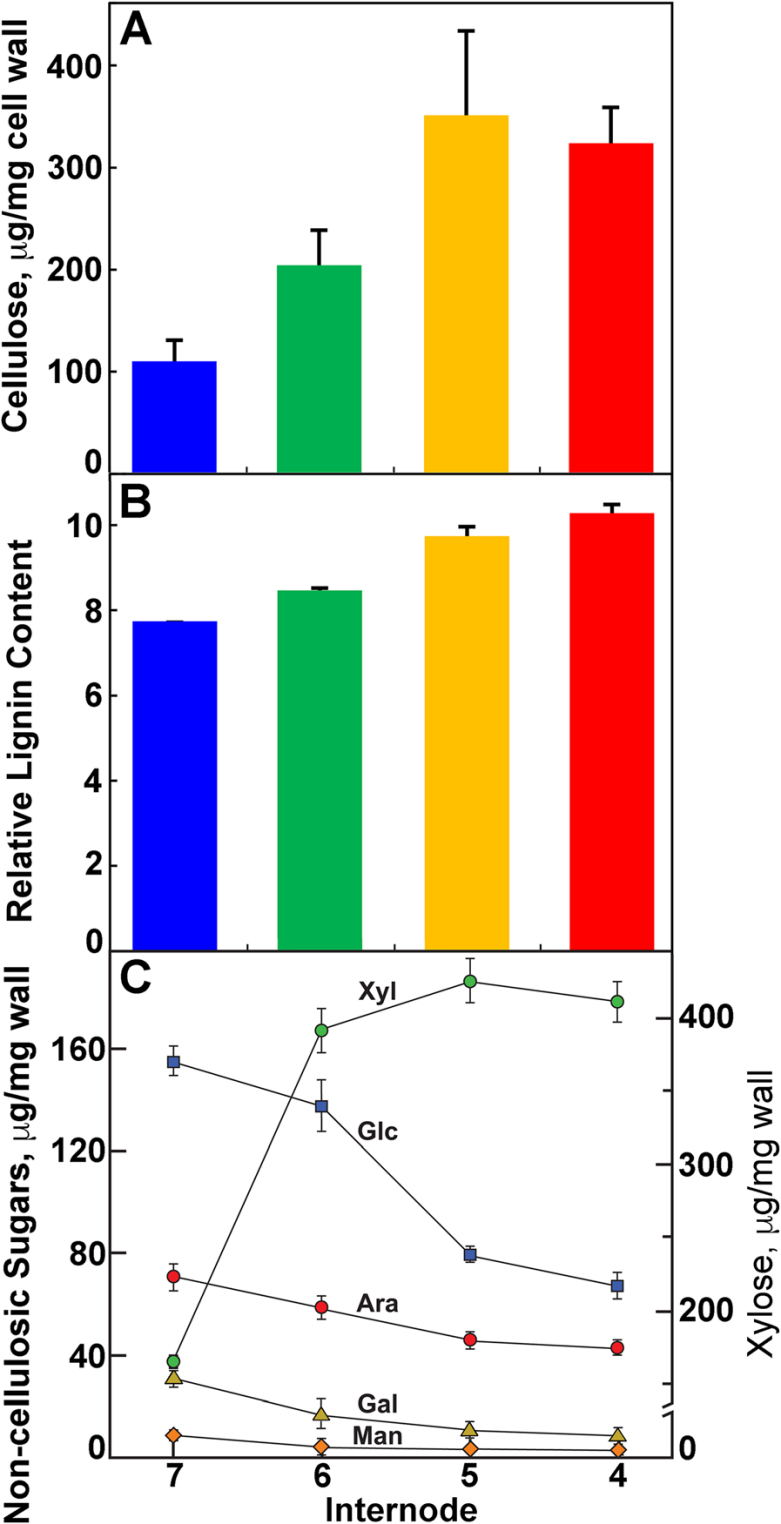
Cellulose, lignin, and xylan content of maize internodes increase with developmental age. **a** Cellulose content in maize stems at 49 days after planting increases towards the base of the stem with the most rapid change between Internodes 6 and 5. Values are mean ± S.D. of three biological replicates. **b** Total lignin abundance estimated by pyrolysis molecular beam mass spectroscopy increases towards the base of the stem, peaking in Internode 4. Values are mean ± S.D. of three biological replicates, except for 7, which is the mean ± variance of two biological replicates. **c** Distribution of non-cellulosic monosaccharides yielded by hydrolysis of cell walls isolated from rind tissues in TFA. Values are mean ± S.D. of three biological replicates

In greenhouse-grown materials, acetic-nitric-insoluble cellulose, a measure of crystalline cellulose content, increased 3-fold in internodes 4 and 5 compared to wall material isolated from internode 7 (Fig. 2a). Lignin, as estimated using pyrolysis molecular-beam mass spectroscopy (PyMBMS), was most abundant in internode 4 (Fig. 2b). Xyl content per gram of cell wall material increased four-fold between internodes 6 and 7 (Fig. 2c). In contrast, the weight % of other major non-cellulosic sugars, Glc, Ara, Gal, and Man, decreased with developmental age of the internodes. Thus, xylan content increased in older internodes, slightly in advance of lignification and cellulose deposition.

### Identification of gene family members for biosynthetic enzymes of cellulose, xylan and lignin in stems

We identified over 70 families and sub-groups of cell wall-related genes that function in nucleotide-sugar and monolignol substrate generation, synthesis and glycosyl transfer, growth, and hydrolysis and transglycosylation in maize B73 (Additional file 1: Dataset 1). We used the MaizeGDB v.2/v.3 for annotation of the cell-wall genes; because of numerous instances of missing genes and annotation errors, our attempts to update sequences with v.4 were unsuccessful. For RNA-seq analysis, we sampled rind tissues of field-grown plants between 35 and 63 days after planting: internodes 8 and 9 represented elongating tissue, internodes 6 and 7 were in transitional stages, and internodes 3, 4 and 5 represented tissues enriched for secondary wall development. Twenty-four maize housekeeping genes [14], were consistently expressed in all tissues except internode 7, which was excluded from subsequent analysis (Additional file 2: Table S1). The gene IDs and expression in reads per 20 M for all genes expressed in the stem internode rind tissues are provided in Additional file 3: Dataset 2.

**Fig. 3:**
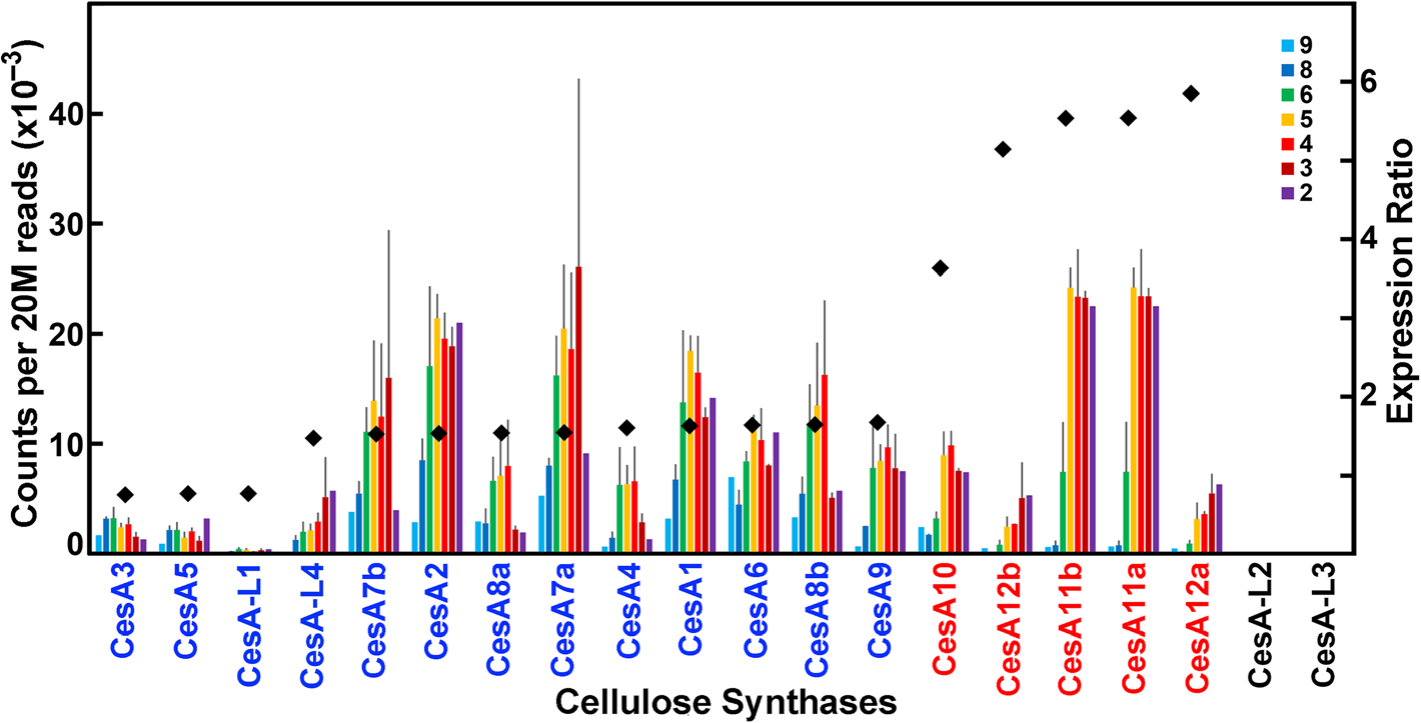
Differential expression of the maize B73 cellulose synthase (CesA) gene family members during stem development. Transcript levels in rind tissues from Internodes 9 through 2 were normalized and compared as counts per 20 M reads. Values are the mean ± variance or S.D. of two or three independent rind collections, respectively. Genes with expression greater than 500 reads per 20 M were ordered by their ratio of expression (black diamonds) in secondary cell-wall-forming tissues (Internodes 5 through 3) to elongating tissue (Internodes 9 and 8). Blue text indicates the closest Arabidopsis homolog to the maize gene is similarly expressed constitutively or in elongating rind tissues, and red text indicates that the closest Arabidopsis homolog to the maize gene is similarly expressed in secondary cell-wall-forming tissues

**Fig. 4:**
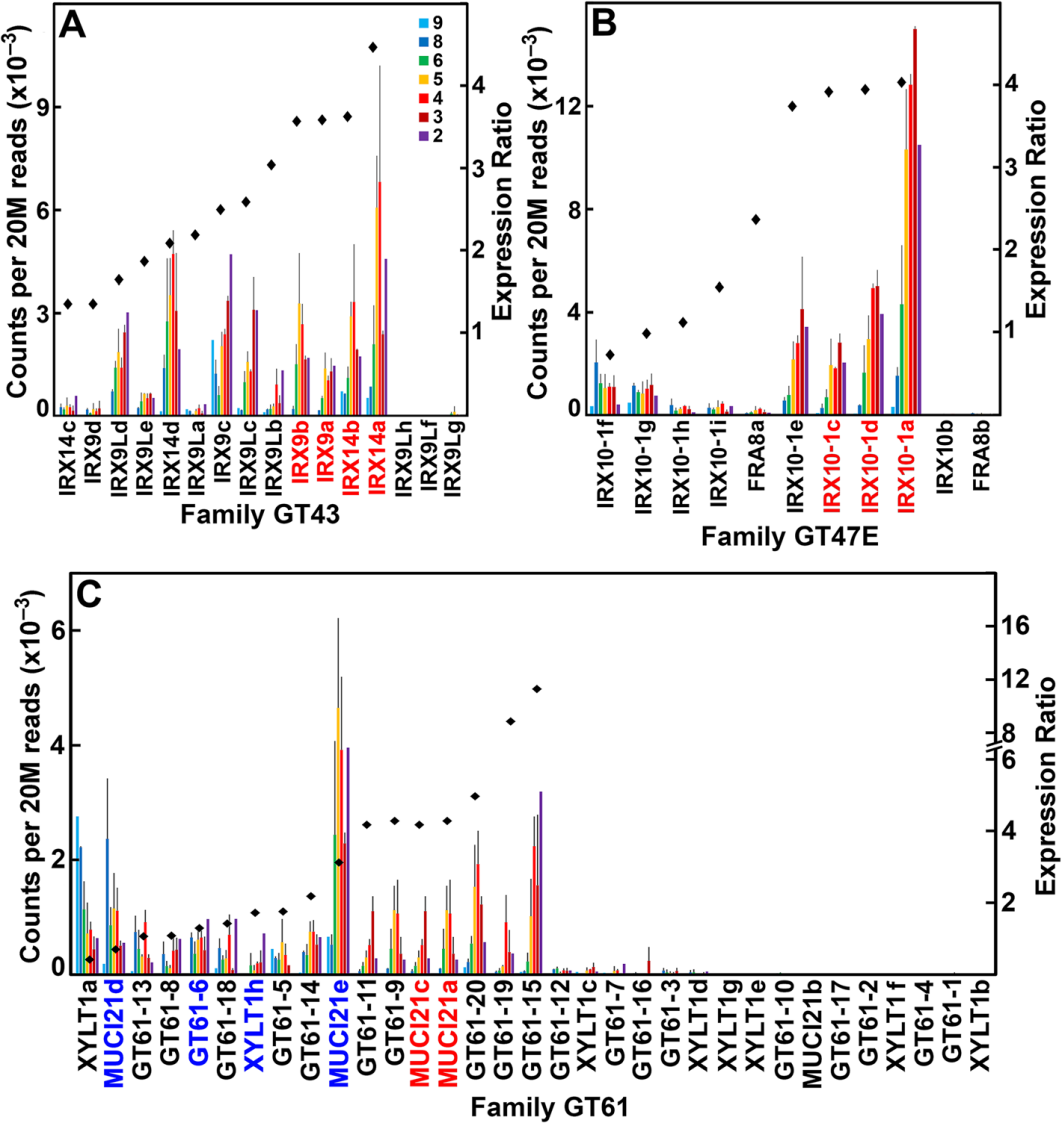
Differential expression of genes of maize B73 in families associated with glucuronoarabinoxylan synthesis during stem development. **a** Family GT43, containing xylan xylosyl transferases. **b** Family GT47 subgroup E, containing xylan glucuronosyl transferases. **c** Family GT61, containing xylan arabinosyl- and xylosyl transferases. Expression ratios and potential Arabidopsis orthologs determined as described in the legend of Fig. 3

**Fig. 5:**
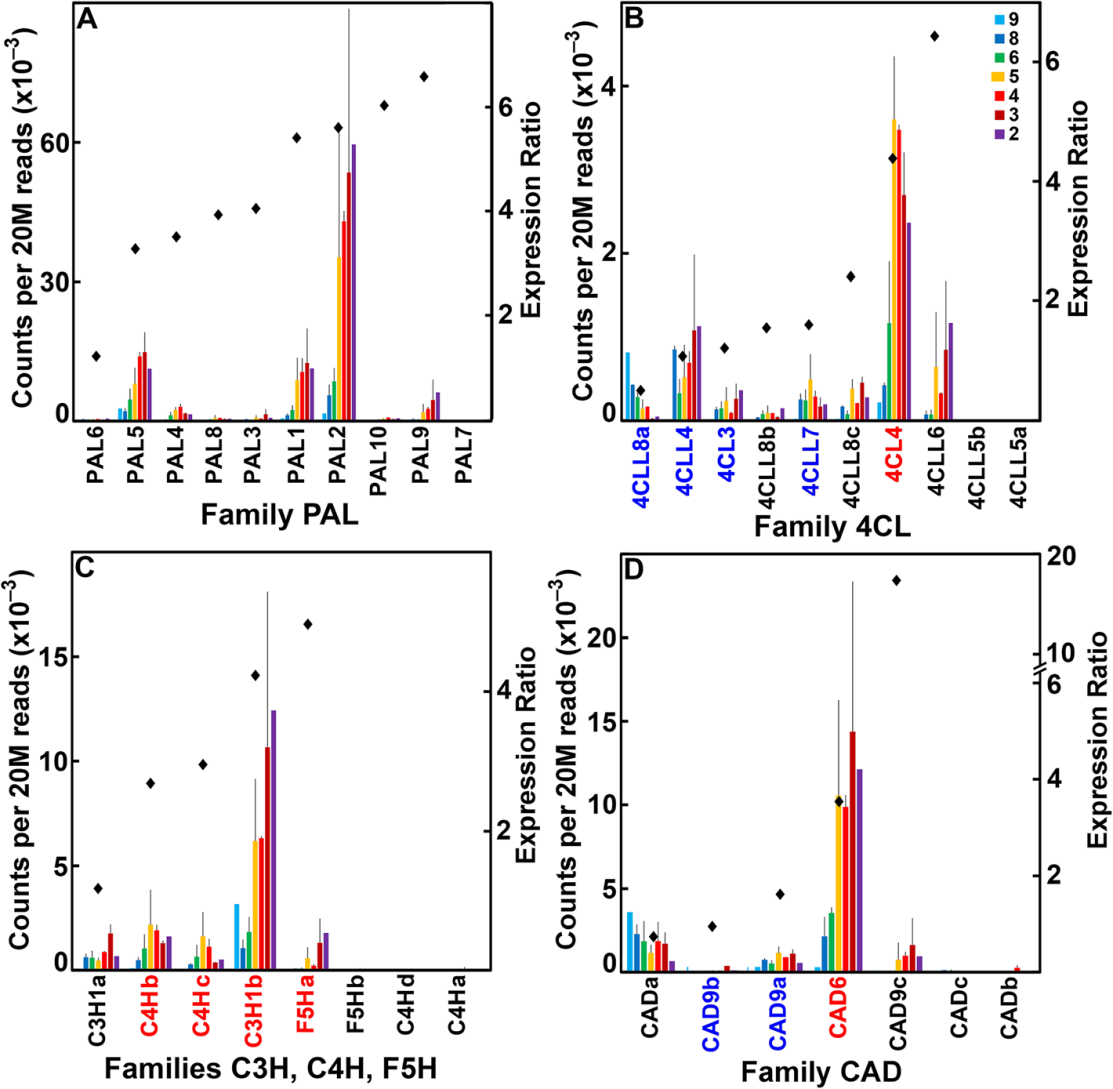
Differential expression of genes of maize B73 in families associated with monolignol synthesis during stem development. **a** Family PAL, phenylalanine ammonia lyases. **b** Family 4CL, 4-coumarate CoA ligases. **c** Families C3H (coumarate-3-hydroxylases), C4H (cinnamate-4-hydroxylases), and F5H (ferulate-5-hydroxylases). **d** Family CAD, cinnamyl alcohol dehydrogenases. Expression ratios and potential Arabidopsis orthologs determined as described in the legend of Fig. 3

Although 854 cell-wall related genes were expressed at ≥95 reads per 20 M, we used a criterion of genes expressed at a threshold of ≥500 reads to reflect significant expression levels in internodes. We used an expression ratio of 2-fold or higher of transcript abundances in internodes 3 through 5 compared to those of internodes 8 and 9 to indicate expression related to secondary wall formation. Conversely, ratios of 1.0 or less indicated genes associated with primary wall formation during internode elongation. Using these criteria, we identified, among 693 cell wall-related genes highly expressed during stem development, 199 genes with greater than 2-fold transcript abundance in older internodes compared to elongating internodes; 171 genes exhibiting intermediate ratios between 1 and 2, and 323 with ratios ≤1 (Table 1; Additional file 1: Dataset 1). About 1/3 of the cell wall-related genes were not expressed or exhibited expression below 95 reads per 20 M. We provide a compendium of the cell wall-related gene families of maize B73, levels of expression in stems, the ratios that predict predominantly primary or secondary wall expression, and Arabidopsis homologs most similar in sequence (Additional File 1: Dataset 1). For most of these families, we plotted those with significant expression across the seven internodes and their ratios of expression during elongation and growth through secondary wall development (Figs. 3-5; Additional file 4: Figures S1-S23).

**Table 1:** Putative orthologous expression of maize and Arabidopsis cell wall-related genes during elongation, transitional and secondary wall stages of stem development

Expression	Secondary/Elongation	Maize	Expression	Putative	Ortholog
Category	Ratio^1^	Genes	Fraction	Arabidopsis	Fraction^3^
				Orthologs^2^	
Elongation	≤ 1.04	323	0.47	275	0.56^3^
Transitional	1.05–1.94	171	0.25	–	–
Secondary	≥1.95	199	0.29	39	0.20
Total Expressed^4^		693(854)	–	314	–
Unexpressed^4^		546(385)	–	–	–
Total Genes		1239			

^1^Ratio of transcripts from rind tissue of Internodes 3 through 5 (Secondary): Internodes 8 and 9 (Elongation) of genes expressed at ≥500 reads per 20 M

^2^Number of potential Arabidopsis orthologs among maize genes expressed with ≥500 reads per 20 M

^3^Fraction of expressed maize genes from elongation and transitional expression that have putative Arabidopsis orthologs

^4^Total genes expressed with ≥500 reads per 20 M. In parentheses are the total genes expressed with ≥95 reads per 20 M

The cellulose synthase (*CesA*) gene family comprises ten genes in Arabidopsis and in rice, but 20 in maize as a result of recent genome duplication [9]. Five *CesA* genes showed 3- to 6-fold increase in transcript abundance in internodes associated with secondary wall formation (Fig. 3; Additional file 1: Dataset 1). Ten *CesA*s had intermediate ratios, and three others were expressed predominantly in younger internodes. Several other gene families are associated with cellulose biosynthesis, as mutations in specific family members result in mutant phenotypes of reduced cellulose content. Of these, the Glycosylphosphatidylinositol (GPI)-anchored ‘*skewed growth*’ *SKU* genes were expressed primarily during elongation (Additional file 4: Figure S1A). GPI-anchored COBRA proteins are implicated in orientation and patterning of cellulose microfibrils during cell elongation [15, 16], but two *COBRA*-like genes, *COBL4a* and *COBL4b*, were expressed during secondary wall formation. Mutations in *COBL4* in Arabidopsis result in weaker floral stems [3], and the *Brittle stalk2* mutation in maize was traced to a mutation in *COBL4a* that results in defects in lignin-cellulose interactions required to maintain stem flexibility [17] (Additional file 4: Figure S1A). The *Glycosyl Hydrolase 9* (*GH9*) gene family includes KORRIGAN (KOR), a membrane-associated endo-β-glucanase [18, 19]. In maize, five *KOR* homologs were expressed broadly across all developmental stages, and two, *GH9B8a* and *GH9B8b*, were differentially expressed during secondary wall formation (Additional file 4: Figure S1B). *SUCROSE SYNTHASE4a*, thought to channel substrate to the active site of CesAs, was expressed at all stages, with low expression of other family members (Additional file 4: Figure S1C).

The GAXs are the major non-cellulosic glycans in the Type II primary walls of grasses [20], synthesized by members of three major families of glycosyl transferases. Members of family GT43 number 16 in maize and are inverting type xylosyl transferases required for xylan backbone synthesis (Fig. 4a), nine of which had expression ratios greater than 2. Family GT47 is a large family of inverting glycosyl transferases; subgroup GT47E, known to contain *IRREGULAR XYLEM10* (*IRX10)* xylan xylosyl transferase genes [21], and comprises 11 genes in maize; five were expressed predominantly during secondary wall formation (Fig. 4b). All members of other GT47 subgroups were more highly expressed during elongation stages or constitutively expressed (Additional file 4: Figure S2). Family GT61 includes members that encode arabinosyl and xylosyl transferases that add these sugars as subtending groups on the xylan backbone. The family comprises 33 genes, seven of which were expressed 2-fold or higher (Fig. 4c). The family of *TRICHOME-BIREFRINGENCE-like* (*TBL-like*) genes encode enzymes involved in acetylation of xylans [22, 23] (Additional file 4: Figure S3). Gene family members *TBLa*, *REDUCED WALL ACETYLATIONa* (*RWAa)*, *RWAe*, *RWA2*, *ALTERED XYLOGLUCAN4A* (*AXY4a*), *AXY9a,* and seven Group E family members were more highly expressed during secondary wall formation.

In contrast to genes encoding other polysaccharide synthases and glycosyl transferases, most of the enzymes of monolignol synthesis were upregulated in older internodes. Eight *Phenylalanine/tyrosine Ammonia Lyase* (*PAL*) genes, two *Cinnamate 4-Hydroxylase* (*C4H*) genes (*C4Hb* and *C4Hc*), a *Coumarate 3-Hydrolase* (*C3H1b*) gene, a *Ferulate 5-Hydroxylase* (*F5Ha*), and two *Cinnamyl Alcohol Dehydrogenase* genes (*CAD6* and *CAD9c*) were more highly expressed during secondary wall formation (Fig. 5a, c and d). Three of the eight expressed *4-Coumarate CoA Ligase* (*4CL*) genes were associated with secondary wall formation, and one, *4CLL8a*, was predominantly associated with elongation stages (Fig. 5b). Fourteen genes of the *Hydroxycinnamoyl-CoA Shikimate/quinate Hydroxycinnamoyl Transferase* (*HCT*) family were expressed at ≥500 reads per 20 M, with five highly expressed during secondary wall formation (Additional file 4: Figure S4A). Four members of the 18-member *Cinnamyl CoA Reductase* (*CCR*) family, *CCR1a*, *CCRL5b*, *CRL1a*, and *CRL1e*, and three of six expressed members of the *Caffeoyl-CoenzymeA 3-O-Methyltransferase* (*CCoAOMT1b, CCoAOMT1d,* and *CCoAOMT1e*) family were associated with secondary wall formation (Additional file 4: Figure S4, B and C).

Peroxidases are encoded by 124 genes classified into seven subgroups (Additional file 4: Fig. S5), and genes encoding laccases numbered 24 (Additional file 4: Figure S6). For these large gene families, only a few genes were expressed in stems. Of 57 expressed peroxidase-encoding genes, and 17 laccase encoding genes (Table 2), 16 and 10, respectively, had expression ratios greater than 2 (Additional file 4: Figures. S5 and S6). The *BAHD* family of acyl-CoA transferases are thought to feruloylate xylans during synthesis [24, 25]. All but four of the 12-member gene family were differentially expressed in secondary cell-wall-forming rind tissues, with *BAHD2a* and *BAHD9* expressed at higher levels (Additional file 4: Figure S7).

**Table 2:** Classification of putative orthologous genes among maize and Arabidopsis for cell wall-related functions. Putative orthology is based on common elongation/primary wall or secondary wall expression profiles of genes with highest sequence similarity (Additional File 1: Dataset 1)^1^

Cell Wall function	Number of Genes (Number expressed)^2^	Putative	Putative
		Primary wall	Secondary Wall
		Orthologs	Orthologs
Sucrose Synthase	8(8)	4	0
Nucleotide-sugar interconversion	46(39)	8	0
Nucleotide-sugar transport (NST)	65(61)	27	0
Cellulose synthase (CesA)	20(19)	12	5
Callose synthase	12(12)	6	0
Cellulose synthase-like (Csl)	35(30)	6	0
Glycosyl Transferase (GT)	265(183)	70	9
Acetyltransferase (TBL/BAHD)	77(61)	1	2
ER-Golgi resident protein	41(36)	17	0
AGP/Glycoprotein/RLK	52(38)	9	1
GPI-anchored protein	22(15)	1	2
Expansin/XTH/Yieldin	97(45)	18	1
Methylesterase/acetylesterase	45(26)	13	0
Polysaccharide Hydrolase/lyase	155(105)	49	3
Protease	51(28)	4	2
Monolignol Synthesis	100(74)	16	9
Peroxidase	124(57)	13	1
Laccase	24(17)	1	4
Total	1239(854)	275	39

^1^Ratio of transcripts from rind tissue of Internodes 3 to 5 (Secondary): Internodes 8 and 9 (Elongation)

^2^Total expression ≥95 reads per 20 M

### Many other gene families have specific family members differentially expressed during secondary wall deposition

Members of nucleotide-sugar interconversion gene families exhibited primarily constitutive expression (Additional file 4: Figure S8). However, at least one gene of almost every family was highly expressed during secondary-wall-formation, including a *UDP-Glc Epimerase* (*UGE2*), a *Rhamnose Synthase* (*RHM1a*), a *UDP-Glc Dehydrogenase* (*UGD3b*), two *UDP-Xylose 4-Epimerases* (*UXE4a* and *UXE4c*), a *GDP-Man 3,5-Epimerase* (*GME1b*), and three *UDP-GlcA Decarboxylases* (*AUD1b*, *AUD3b*, and *AUD3c*). Five members of the 9-member GT75 *UDP-Ara Mutase* (*UAM*) family known to function in conversion of UDP-Ara*p* to UDP-Ara*f* were expressed, with two members, *UAM1b* and *UAM5a*, with ratios above 2 (Additional file 3: Figure S8F). At least one member in five of the six classes of nucleotide-sugar transporters exhibited over 2-fold higher expression during secondary wall formation (Additional file 4: Figure S9).

Of the *Cellulose Synthase-like* (*Csl*) genes (Additional file 4: Figure S10), only the most highly expressed *CslD3a* gene (Additional file 4: Figure S10B), and two *CslC* genes (*CslC12a* and *CslC12b*) (Additional file 4: Figure S10C), had expression ratios greater than 2. Among flowering plants, the mixed-linkage (1 → 3),(1 → 4)-β-D-glucans (MLGs) are found in grasses and related Poales species [26]. MLGs are synthesized and secreted during cell elongation, where they coat cellulose microfibrils and interact with other wall matrix polysaccharides during growth [27], and are largely degraded after elongation [28]. No *CslF* genes that encode mixed-linkage β-glucan synthase unique to grasses had ratios above 2, but three *CslF* genes were highly expressed lower and middle internodes (Additional file 4: Figure S10E), consistent with the presence of MLG in rice secondary walls [29]. No member of GT34 *Xyloglucan Xylosyl transferase* (*XXT*s) had a ratio greater than 2 (Additional file 4: Figure S10D). All twelve callose synthase genes were expressed, with only two highly expressed during secondary wall formation (Additional file 1: Dataset 1).

Retaining glycosyl transferases of family GT8 are involved in pectin synthesis and and xylan side-group attachment. All members of GT8D, the *Galacturonosyl Transferase* (*GAUT*) gene family, were expressed at ≥95 reads per 20 M during elongation and primary wall formation or constitutively expressed (Additional file 4: Figure S11A; Additional file 1: Dataset 1). Of the *Galacturonosyl Transferase-like* (*GATL*) genes, only *GATL7b* showed high secondary wall expression (Additional file 4: Figure S11B). In contrast, three members of the 7-member *Glucuronosyl Transferase* (*GUX*) family (*GT8A*), which attach α-GlcA residues on GAX, were more highly expressed during secondary wall formation (Additional file 4: Figure S11C). Genes involved in synthesis of RG-I include those of family GT106 subgroup A *Rhamnosyl Transferases* (*RRT*s) (Additional file 4: Figure S12A) [30]. The GT106 family also include members that contain putative *Mannan synthesis-related transferase* genes in subgroup B [31] and *Pectin Arabinogalactan Synthesis-Related (PAGR)* genes in subgroup C [32] (Additional file 4: Figure S12, B and C). Three of the four *RRTs* were expressed, one of them during primary wall formation, and one *RRT1b*, with an expression ratio above 2. *(*Additional file 4: Figure *S12, B and C).*

With the exception of *PGaseA11* and *PGaseA12*, numerous polygalacturonase genes in six families and *RG-I lyases* of the PL4 family were expressed mostly during primary wall formation (Additional file 4: Figure S13). Groups D and E, and many Group B and C members of the GH17 family associated with hydrolysis of (1 → 3)-β-glucans, including side-chains of AGPs and callose, were expressed during elongation stages, but most members of Group A, and a *GH17B13*, and three members of Group C (*GH17C12*, *GH17C13*, and *GH17C14*) had high expression during secondary wall formation (Additional file 4: Figure S14). Expression of *β-Galactosidase* (*BGAL*) genes of family GT35 were in two clusters, one associated with primary wall formation, and one with intermediate ratios (Additional file 4: Figure S14F).

Two, *FLA2a* and *FLA11*, of ten members of the *AGP/Fasciclin-like* gene family showed secondary wall expression (Additional file 4: Figure S15). Family GT31 represents a large family of six sub-groups and includes GalTs that are predicted to form the (1 → 3)-β- and (1 → 6)-β-linked galactan chains of type II AGPs. Three members of GT31A, *GALT4e*, *GT31E1*, *GT31E2*, and two members of GT31F were differentially expressed during secondary wall formation (Additional file 4: Figure S16). For activities atypical of grass cell walls, one GT37 fucosyl transferase, *FUTL11*, and one GT77 arabinosyl transferase had expression ratios above 2 (Additional file 4: Figure S17).

ER-resident glycosyl transferases involved in *N*-linked glycoprotein synthesis were either expressed constitutively or in elongation-associated patterns, except for GT14 *GLCAT14Ac* and *GLCAT14Ad*, and *GT17–3* (Additional file 4: Figure S18), whereas no members of Golgi-resident GT10, GT64, or GT66 gene families had ratios above 2 (Additional file 4: Figure S19). No *Prolyl-4-hydroxylase* genes showed expression above a ratio of 2 (Additional file 4: Figure S20A). Expression of the large receptor-like kinase family fell into three groups: high elongation expression, transitional expression, and five highly expressed in secondary wall formation (Additional file 4: Figure S20B). Several types of cell-wall protease genes were differentially expressed in secondary wall formation, notably four *Aspartyl Protease* genes, and two *Metalloprotease* genes, *MPL1d* and *MPL1e* (Additional file 4: Figure S21).

Expansins and the GH16 family of XTHs are implicated in stress relaxation associated with cellulose microfibril separation during growth and the rejoining of XyGs to maintain tensile strength, respectively [33, 34]. Most *α-Expansin* (*α-Exp*), *α-Expansin-like* (*α-Exp-like*), and *β-Expansin (β-Exp*) genes were expressed during elongation growth, but an *α-Exp-like2c* and *α-Exp-like2d*, and five *β-Exp* genes were expressed during secondary wall formation (Additional file 4: Figure S22). Similarly, most members of the three sub-groups of *Xyloglucan Endotransglucosylase/Hydrolase* (*XTH*) genes were expressed during elongation and primary wall stages of growth, but five subgroup *XTHB* genes and two subgroup *XTHC* genes were differentially expressed during secondary wall formation (Additional file 4: Figure S23).

### Patterns of cell wall-related gene expression are complex

**Fig. 6:**
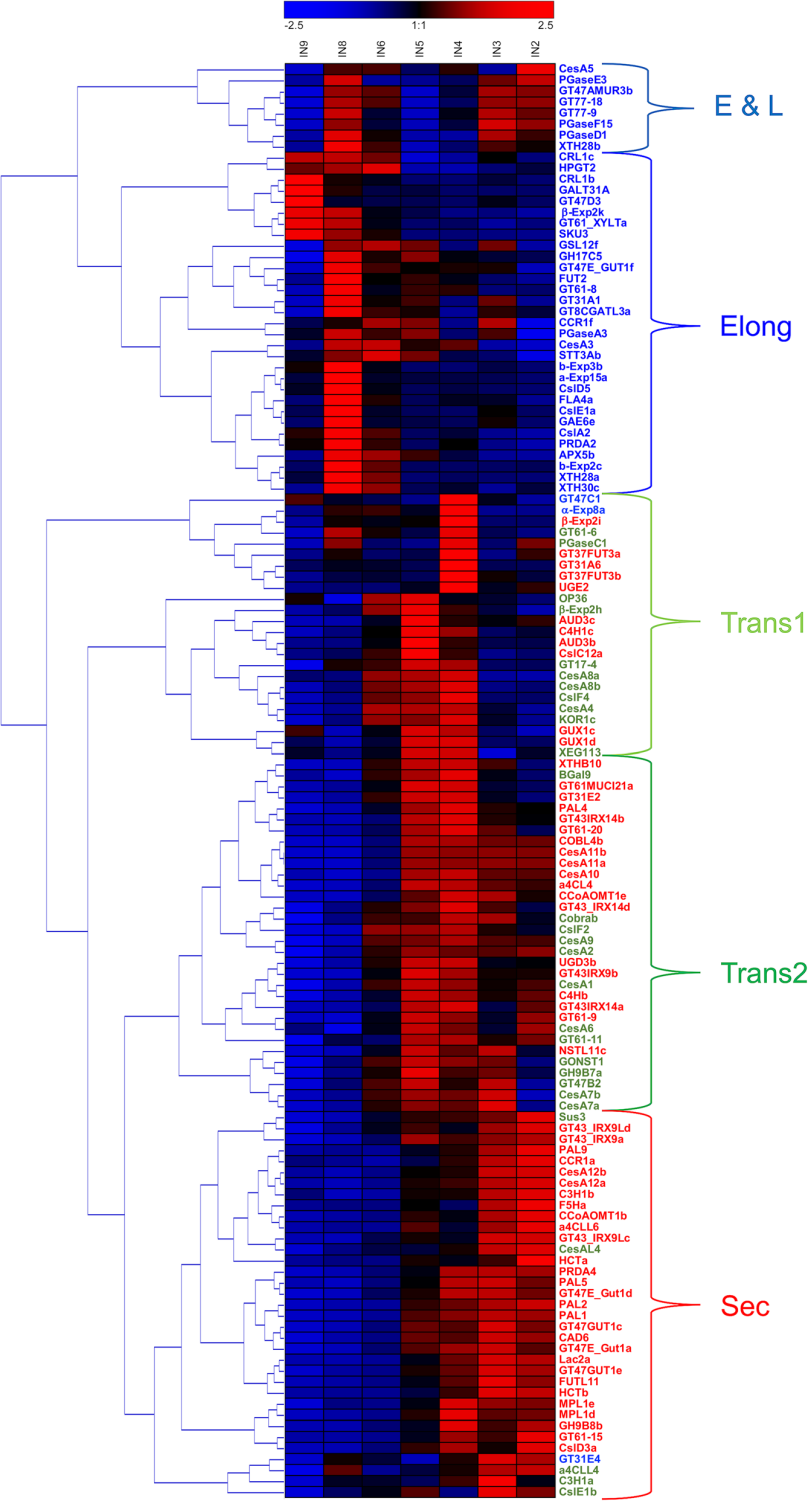
Hierarchical clustering reveals a complex pattern of cell wall gene expression in maize stem tissue. Transcript levels in rind tissues from internodes 2–9 were normalized and grouped by hierarchical clustering. Thirteen subclades were grouped into five distinct patterns representing an Elongation (Elong) stage, two transitional (Trans1 and Trans2) stages, a secondary wall development (Sec) stage, and an Early and Late (E&L) stage. Genes comprising these clusters are colored by ratio of Transitional/Secondary wall stages (Internodes 5 through 3) to Elongation stages (Internodes 8 and 9). Genes with expression ratios ≤1.04 are in blue, ratios between 1.05 and 1.94 in green, and ratios ≥1.95 in red

**Fig. 7:**
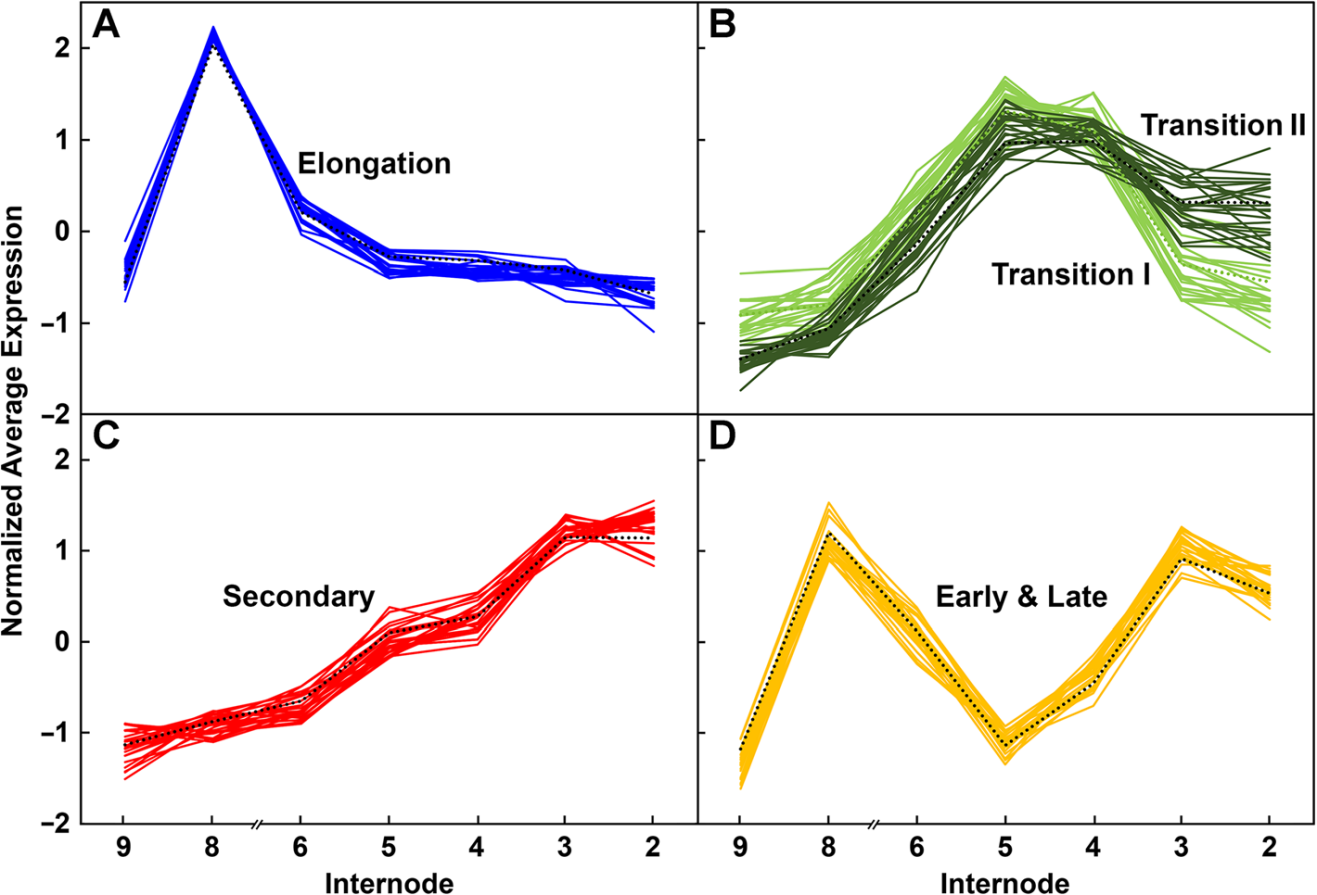
Expression patterns of maize B73 genes during stem development derived from hierarchical clustering. Transcript levels in rind tissues from Internodes 2 through 9 were normalized. **a** Pattern 1 (Elongation) shows highest expression in the younger internodes, a stage associated with elongation stages and primary wall formation. **b** Pattern 2 shows low expression during elongation stages, with either low (Transition I) or high (Transition II) expression in older internodes. **c** Pattern 3 (Secondary) shows low expression in younger internodes and increasing in older internodes. **d** Pattern 4 (Early & Late) shows moderate to high expression during early elongation stages, decreased expression during peak secondary wall formation, and returns to elevated expression during late secondary wall formation

Of 693 genes with ≥500 reads per 20 M, 171 displayed an expression ratio between 1 and 2, and their profiles across the seven internodes indicated more complex patterns of expression. We applied Hierarchical Clustering (HC), with average linkage clustering, and Principal Components Analysis (PCA) to the patterns of 134 of the most highly expressed cell wall-related genes across Internodes 2 through 9. Although thirteen distinct clades were clustered (Fig. 6), these could be grouped by five patterns corresponding to genes highly expressed during elongation, two subclasses of genes expressed during transition to secondary wall formation, genes expressed during secondary wall formation, and genes with high expression during both early and late development but with lower expression during transitional stages (Fig. 7). The Elongation pattern was matched by genes of growth and development, including several expansins, XTHs, and AGPs; the two Transitional patterns were matched by a majority of the *CesA*s, and several synthases and glycosyl transferase genes involved in GAX synthesis. Secondary wall *CesAs* and genes of monolignol synthesis matched the late Secondary wall pattern (Fig. 6; Additional file 5: Dataset 3). Genes categorized into these five stages by HC could also be clustered with little overlap by exploratory PCA, with over 80% percent of variance accounted for by the first three PCs (Additional file 6: Figure S24). Loading 1 was similar to the early Elongation pattern, whereas loading 2 was similar to Transitional patterns, and loading 3 showed similarity to the Early and Late pattern.

We then applied a slope-metric pattern-matching algorithm genome-wide for genes that best matched the five patterns established by highly expressed cell wall-related genes (Fig. 7; Additional file 5: Dataset 3). The number of genes within one standard deviation varied widely, from 750 and 492 genes for the Elongation and Early & Late patterns, respectively, to 107 and 54 for the two transitional patterns (Additional file 5: Dataset 3). Genes of unknown function represented 40 and 46% of genes matched to the Elongation and Early & Late patterns, respectively, and cell wall-related genes represented 5 and 6% (Additional file 7: Figure S25). By contrast, the proportions of cell wall-related genes increased from 9% with the Transition I pattern, to 18% with the Transition II pattern, to 28% with the Secondary wall pattern. Genes associated with apoptosis were less than 1% of all patterns except the secondary wall pattern, where they represented 6% (Additional file 7: Figure S25). In the 72 genes matching the Secondary pattern, several genes were identified that encode transcription factors, including a *NAC* gene and a *MYB* gene, or are involved in signal transduction, including several *MAP kinase* genes, and also several classes of genes of metabolism and its regulation, synthesis and cellular function (Additional file 5: Dataset 3).

### Comparison of expression profiles of maize and Arabidopsis cell wall-related genes indicates only limited orthology

We compared maize expression profiles of genes homologous to those expressed during Arabidopsis floral stem development [3]. Homologs closest in sequence that were similarly expressed in primary or secondary wall-enriched internodes were considered putative orthologs. All but two of the 19 expressed members of the CesA gene family were putatively orthologous to Arabidopsis sequences (Table 2, Fig. 3). Five of 13 laccases expressed at ≥500 reads per 20 M had putative orthology to Arabidopsis sequences, with four of them more highly expressed during secondary wall formation (Additional file 4: Figure S6). By contrast, fewer orthologs were found among members of all other maize gene families with Arabidopsis genes, with more associated with synthesis of primary wall than of secondary wall (Table 2, Additional file 1: Dataset 1). Of the 693 cell-wall related maize genes expressed during stem development at ≥500 reads per 20 M, about 56% of those were associated with primary wall synthesis were putatively orthologous with an Arabidopsis gene, but only 20% of maize genes highly expressed during secondary wall formation were putative orthologs (Tables 1 and 2). Using a combination of ratio of expression and expression pattern as criteria, other putative orthologs of Arabidopsis genes with secondary wall expression included two *IRX9* and two *IRX14* genes associated with xylan synthesis (Fig. 4a), and three *IRX10* genes (*IRX10–1a, IRX10–1c*, and *IRX10–1d*) associated with xylan synthesis (Fig. 4b). Two of 22 expressed GT61 genes associated with xylosyl- or arabinosyl side-group addition to GAX (*MUCI21a* and *MUCI21c*), were putatively orthologous with Arabidopsis genes expressed during secondary wall formation, and five other GT61 genes were putatively orthologous with Arabidopsis sequences expressed during primary wall synthesis (Fig. 4c). The highest proportion of genes encoding secondary wall-related synthesis putatively orthologous with Arabidopsis were those in monolignol and lignin synthesis (Table 2). In several families of monolignol synthesis in maize, *4CL*, *C3H*, *C4H*, *F5H*, *CCoAOMT*, *HCT*, and *CAD*, the most highly expressed member was closest in sequence with an Arabidopsis homolog during secondary wall formation (Fig. 5; Additional file 4: Figure S4, A and C).

Several of the putatively orthologous genes of nucleotide sugar interconversion, and their transport, and callose synthases were differentially expressed during primary wall formation, but none was potentially orthologous with one expressed during secondary wall formation (Table 2; Additional file 4: Figures. S8, S9, and S10G; Additional file 1: Dataset 1). Only a few members of the maize *Csl* family, and of pectin synthesis and depolymerization, were expressed predominantly during secondary wall formation, none of which had an apparent Arabidopsis ortholog. In summary, for gene families involved in cellulose and lignin biosynthesis, putative orthologs were identified. For the majority of other gene families, most putative orthologs were primary wall expressed and only rarely was the most highly expressed maize gene potentially orthologous to an Arabidopsis gene involved in secondary wall formation (Table 2, Additional file 4: Figure S9-S23). Four exceptions were a *Fasciclin-like FLA11* gene involved in AGP core synthesis (Additional file 4: Figure S15), an *XTH30b* involved in XyG transglucosylation (Additional file 4: Figure S23), and two metalloprotease (*MPL1d* and *MPL1e*) genes (Additional file 4: Figure S21B).

### Novel promoter motifs for secondary wall synthesis were identified

**Fig. 8:**
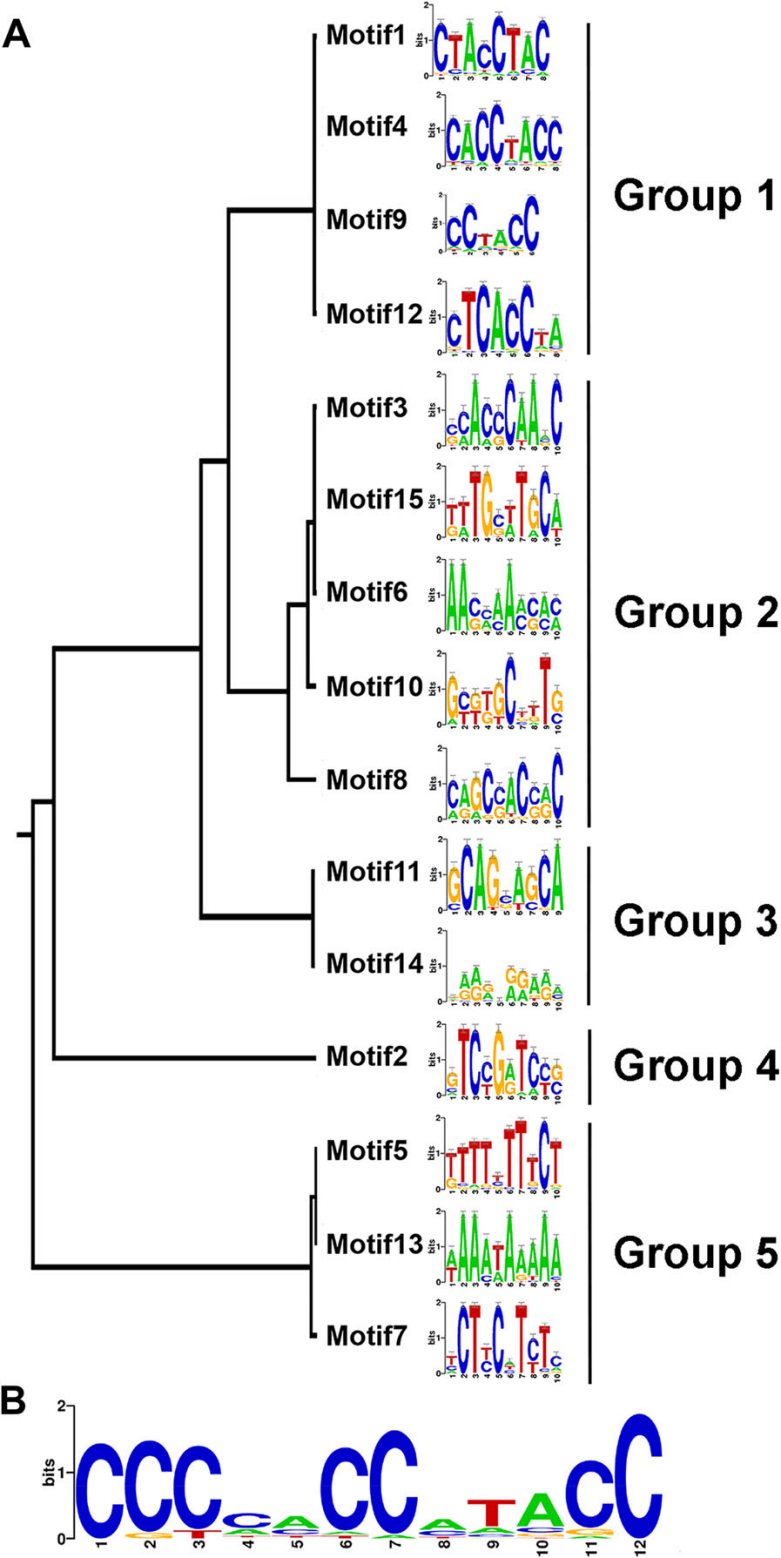
Potential promoter response element motifs are associated with the Secondary wall pattern of expression during maize B73 stem development. **a** Fifteen motifs defined by the STAMP algorithm [36] were clustered in five groups. **b** Consensus sequence derived from overlapping motifs of Groups 1 and 2

Analyses of upstream regions of the five sets of co-expressed genes established common promoter motifs among the genes associated with each of the five patterns. As defined by the Promzea pipeline [35], using the on-line web tool ‘STAMP’ for exploring DNA-binding motif similarities [36], and by Plant PAN 3.0 [37], fifteen overlapping sequence motifs clustered into five groups were identified within 1 kb of sequence immediately upstream from the transcriptional start sequence among the 72 genes matching the secondary wall pattern (Fig. 8a; Additional file 8: Table S2). Known promoter motifs found using STAMP for the secondary cell wall-related expression group included MYB and PALBOXA motifs with expect values between 10^− 7^ and 10^− 10^ and considered associated with the Promzea-defined motif. Four Group 1 motifs, with consensus sequence CC(TA)CC, were represented in most of the genes (Fig. 8b; Additional file 9: Table S3). This sequence is consistent with the motif CCWACC defined for a P Myb factor [38] and a longer sequence associated with a promoter of PAL2 activated during lignification of loblolly pine [39, 40]. Secondary wall *CesAs*, *IRX9*, *PAL9*, *CCR1* and *C2H1b* also have Group 2 motifs 3 and 15 in their promoters (Additional file 8: Table S2). Group 1 motifs are underrepresented in promoter regions of *C3H1b* and *Lac2a* (Additional file 8: Table S2). The PALBOXA promoter motif, CCGTCC, a sequence in promoters of lignin biosynthesis genes [41, 42], matched perfectly eight of the maize secondary wall genes defined by the slope-metric algorithm; although missing only the last nucleotide in 10 others, the motif occurred multiple times within genes with other functions (Additional file 9: Table S3). Many of the genes also had a related SBOXATRBCS motif associated with ADP-ribosylation factors involved in signal transduction of biotic- and abiotic stresses [43, 44], with matches to AGTACSAO, a motif associated with response to drought stress [45, 46].

### Two common maize inbred lines have two-fold or greater difference in transcript abundances for 70% of cell wall-related genes

Greenhouse-grown B73 and Mo17 inbred lines had similar patterns of cellulose, lignin, and xylan accumulation in their internodes to those of field-grown B73 plants (Additional file 10: Figure S26). Transcript profiles were determined for cell wall-related genes from the rind tissues of four internodes in Mo17 and B73, at 49 days after planting. Internodes 4 and 5 in both inbred lines expressed secondary wall-related genes and Internodes 6 and 7 had transcript abundances characteristic of elongation-related genes. However, the higher accumulation of Xyl in Int 7 (Additional file 10: Figure S26C) and higher transcript abundances of many secondary wall-related genes in Mo17 indicated an earlier onset of secondary wall development (Additional file 11: Dataset 4). For simplicity of comparison, we averaged the elongation-related Internodes 6 and 7 and secondary wall-rich Internodes 4 and 5 for each genotype. No bias was found between the two genotypes with respect to abundances of transcripts based on size (Additional file 12: Figure S27). However, 60 to 70% of all genes expressed in B73 and Mo17 stem internodes showed greater than a two-fold difference in transcript abundance at both stages. About 30% of genes were differentially expressed at the five-fold level, and 1 to 2% at the > 100-fold level (Additional file 13: Table S4). Often, genes with > 100-fold differences resulted from the absence of the gene in one of the inbred lines. A noteworthy example was a particularly large deletion in chromosome 6, where 2.7 Mb is absent from the Mo17 genome and 53 B73 genes are measured as differentially expressed at 26- to 500-fold greater levels (Additional file 12: Figure S28). We have summarized the fold-changes for the cell-wall related genes expressed by B73 and/or Mo17 (Additional file 11: Dataset 4), and provided a compendium for comparative elongation- and secondary-wall stage-specific expression profiles between B73 and Mo17 (Additional file 12: Figures. S29-S52). The gene IDs and expression in reads per 20 M for all genes of B73 and Mo17 expressed in the stem internodes are also provided (Additional file 14: Dataset5).

**Fig. 9:**
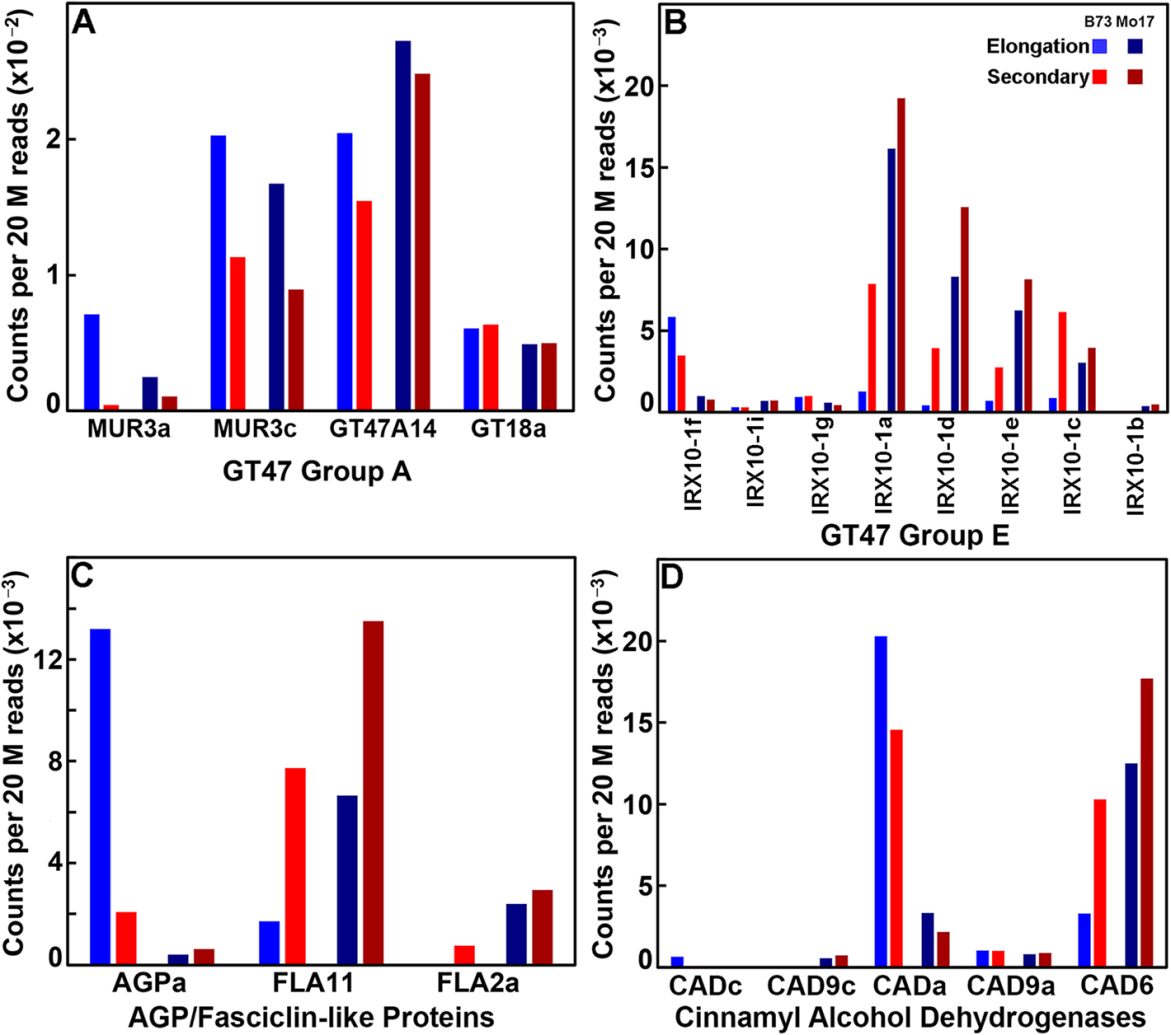
Maize inbreds B73 and Mo17 exhibit large differences in gene expression during elongation and secondary wall stages of stem development. Transcript levels in rind tissues of greenhouse-grown plants taken at elongation stages (Internodes 8 and 6) and secondary wall synthesis stages (Internodes 5 and 4) of each inbred were pooled and normalized and compared as counts per 20 M reads. **a** GT47 Group A xyloglucan galactosyl transferase genes showing relatively common expression. **b** GT47 Group E xylan xylosyl transferase genes that show the same relative patterns of expression but significant expression fold-differences. **c** AGP/Fasciclin-like proteins that show unique patterns of expression that result in fold-change differences. **d** Cinnamyl alcohol dehydrogenase (CAD) genes that show dominant expression of different genes that result in fold-change differences

Although Copy-Number (CNV) and Presence-Absence (PAV) variation might account for some of the large fold-differences in expression, single-nucleotide polymorphisms within the promoters of genes might also contribute to differential expression between the two inbreds. For many cell wall-related genes, expression in B73 and Mo17 followed the same pattern, but varied in transcript abundances, as exemplified by *MUR3c*, *GT47A14*, and *GT18a* (Fig. 9a). Three B73 genes, *MUR3a*, *IRX10–1f*, and *IRX10–1c* have higher levels of expression at either elongation or secondary wall stages (Fig. 9a and b). In Mo17, *IRX10–1c* exhibited a higher fold-change during elongation stages but lower fold-change during secondary wall formation (Fig. 9b). However, three AGP-related genes show distinctive expression patterns. The B73 *AGPa* was expressed at levels over 10-fold greater than that of Mo17, whereas the Mo17 *FLA2a* exhibited higher expression levels at both stages (Fig. 9c). Similarly, the *CADa* gene was more highly expressed in B73, whereas *CAD6* was more highly expressed in Mo17 (Fig. 9d). In these two instances, mutations within several MYB- and/or NAC-related motifs in Mo17 *CADa* and B73 *FLA11* might be causative of reduced expression (Table 3).

**Table 3:**
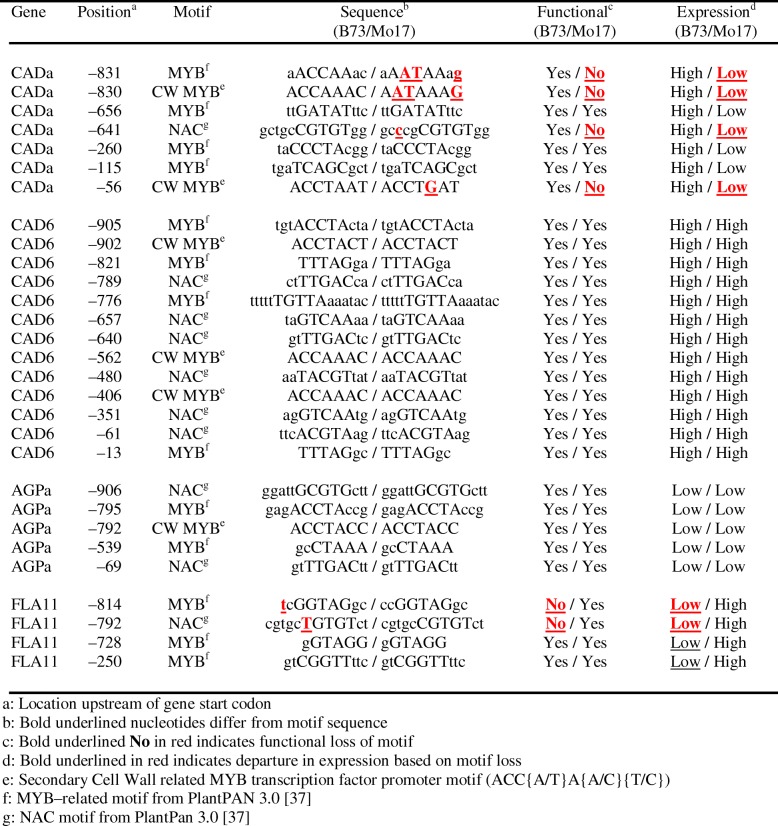
MYB- and NAC-related promoter locations indicating putative mutations in four genes with differential expression between B73 and Mo17

a: Location upstream of gene start codon

b: Bold underlined nucleotides differ from motif sequence

c: Bold underlined **No** in red indicates functional loss of motif

d: Bold underlined in red indicates departure in expression based on motif loss

e: Secondary Cell Wall related MYB transcription factor promoter motif (ACC{A/T}A{A/C}{T/C})

f: MYB–related motif from PlantPAN 3.0 [37]

g: NAC motif from PlantPan 3.0 [37]

## Discussion

Grass species have been bred for centuries for food and feed, but exploiting the genetic diversity of grasses for improved utility as biomass feedstocks in the production of fuels and chemicals has been a much more recent endeavor. As a result, current feedstocks are not optimized for energy efficiencies in downstream conversion processes [1]. The optimization of biomass for cell wall deconstruction depends on identification of the regulatory networks that control secondary wall formation and the genes involved in its construction and assembly. Work initiated in Arabidopsis [3, 47–49] has been extended to poplar and other dicot woody species [4, 5], but a deeper understanding is needed of the molecules and architectures characteristic of grass cell walls as distinct from those of other dicots and non-commelinid monocots [50]. These compositional differences are reflected in the phylogeny of membership in cell wall-related gene families between the eudicot *Arabidopsis thaliana* and two grasses, rice and maize [8]. An ancient tetraploidy event greatly expanded the number of paralogs within each family of the maize genome. Here, we identify the specific gene family members and potential *cis*-regulatory motifs of the major cell-wall relevant families associated with secondary wall development in two elite inbreds. We find that it is common for different family members to be expressed, or vary more than two-fold in the level of expression, between B73 and Mo17, and compared to Arabidopsis. The implications for improvement of bioenergy grasses are two-fold: first, improvement of a single genotype might be predicated on identifying the specific regulatory genes and gene network members, and second, mining genetic diversity across the entire species might provide substantial opportunity to modulate cell wall composition and architecture.

### Expression analyses of stem development define the compendium of maize secondary cell-wall related genes

In grasses, alteration of lignin content and/or composition improves both feed digestibility [51, 52] and saccharification yields [53–55]. However, even changes in low-abundance components, such as pectin, can have a dramatic impact on the yields of glucose and xylose in saccharification assays with poplar wood [56, 57]. We used RNA-seq to develop a comprehensive overview of specific isoforms of cell wall-related genes that are expressed during stem development among over 70 families and their subgroups.

We constructed a simulated time course from seven internodes of stem development. Using validated sets of cell-wall genes known to be involved in primary or secondary wall synthesis, we used a ratio of relative transcript abundance of 2.0 or greater of older vs. younger internodes to identify comprehensive sets of genes associated with secondary wall formation as distinct from elongation growth and primary wall synthesis. Compared to Arabidopsis stem development [3], maize members of the same gene families are represented but the specific homologs expressed are not necessarily the most similar in sequence.

We showed previously that no obvious orthology was evident in the structures of many of the multigene families of cell wall-related genes, and some subclades were unique to the grass species and others unique to dicots [8]. An exception is the CesA family, where specific clades are populated by members of both grass and dicot species [8, 58]. At least three isoforms are expressed during primary wall formation and a separate set of three isoforms is expressed during secondary cell wall synthesis [3, 59–61]. Quantitative antibody-labeling studies indicated that these isoforms are present in 1:1:1 ratios in Arabidopsis [62, 63], indicating CesA complexes of six specific heterotrimer interactions that account for an 18-chain microfibril. However, the equal ratios of three isoforms do not hold for poplar species [64] or maize [65]. Five maize genes *ZmCesA10*, *ZmCesA11a* and *b*, and *ZmCesA12a* and *b* are highly expressed during secondary wall formation and have the highest sequence similarity with Arabidopsis secondary wall cellulose synthases, *AtCesA4*, *AtCesA7*, and *AtCesA8* [58]. Unlike Arabidopsis and rice, more than three primary and secondary wall-related CesAs are expressed in maize and at different levels (Fig. 3). Differential expression of ten primary wall CesAs of the maize coleoptile resulted in different abundances of their isoforms as determined by proteomic analysis [65].

For synthesis of xylan backbones, putative orthologs are found in Family GT43 Xylan xylosyltransferases, defined by *irregular xylem* (*irx*) mutations [66, 67], as two *IRX9* and *IRX14* genes are among the more highly expressed genes during secondary wall development (Fig. 4; Additional file 1: Dataset 1). In contrast, few potential orthologs are found among genes involved in addition of side-groups. Three orthologs of Family GT47E xylan xylosyl transferase (*IRX10–1*) genes [68, 69] are among the highest expressed during secondary wall formation; but the two closest homologs, *MUCI21a* and *MUCI21c*, in the large GT61 family of arabinosyl and xylosyl transferases [70, 71] (Additional file 12: Figure S49B), are not among the most highly expressed maize genes (Fig. 4; Additional file 1: Dataset 1). Consistent with the presence of a phenylpropanoid network in primary walls of grasses, phenylpropanoid biosynthetic enzymes were expressed throughout stem development. With the exception of *PAL* and *CCR* gene families, for which no obvious secondary-wall orthologs were detected, the most highly expressed genes of all genes of monolignol synthesis were most similar in sequence to the respective Arabidopsis family members (Fig. 5; Additional file 4: Figure S2). Thus, maize genes encoding the biosynthetic enzymes for the major secondary wall constituents, cellulose, xylans, and monolignols, are more likely to be orthologous with those of Arabidopsis. However, distinct isoforms of nucleotide-sugar interconversion enzymes and their transporters, other polysaccharide synthases and glycosyl transferases associated with primary wall formation, such as CslF MLG synthase, GPI-anchored COBRA proteins, KORRIGAN family of endo-glucanases, and wall modifying enzymes, such as expansins and XTHs, are expressed during secondary wall formation.

### Patterning matching and comparison of expression profiles found in B73 to Mo17

Five characteristic patterns of gene expression were identified by HC, including one characteristic of secondary wall formation as judged by expression of lignin biosynthetic genes and secondary wall CesAs. As each multi-gene family contains members that have distinct co-expression profiles, we next probed promoter sequences to map response elements common to genes with each of the five patterns. We found some elements common to Arabidopsis promoters, but we identified several novel *cis*-elements, and multiple numbers of them, in the maize promoter sequences of genes expressed during secondary wall formation, including potential binding sites for MYBs and transcription factors.

Maize is recognized for its remarkable variation in genome structure among its many haplotypes [72], as well as CNV and PAV in genome content among its many landraces and inbreds, and its progenitors [11, 73]. Consistent with the findings of Paschold et al. [74] and Baldauf et al. [75] a substantial number of cell-wall related genes were differentially expressed in Mo17 compared to B73, two elite inbreds. While single-nucleotide polymorphisms (SNPs) and small insertion/deletions (InDels) can account for most fold-differences in expression [11, 73], CNV and PAV are likely to account for the large differences, greater than 10-fold, in expression [11, 73, 76]. The deletion of 53 genes in B73 absent from Mo17 across a 2.7 Mb sequence of Chromosome 6 in B73 is an example of significant PAV (Additional file 12: Figure S28). The extensive CNV and PAV in B73 and Mo17 are not significantly biased to one inbred over another [73, 76–78]. Consistent with this assessment, we find little bias in distribution of transcript sizes from either inbred (Additional file 12: Figure S27).

In addition to CNV and PAV, instances of differential gene expression are related to SNPs or small Indels within promoters, possibly resulting in mutation of a functional response element. Five *IRX10* genes involved in xylan synthesis are expressed to greater than 2-fold (Fig. 9b), and this variability might be attributed to the early onset of secondary wall formation in Mo17. In other instances, a different gene family member is expressed more highly. The *CAD6* is more highly expressed during secondary wall formation in Mo17 than B73, but *CADa* expressed during secondary wall formation is greatly attenuated in Mo17 compared to B73 (Fig. 9d). In this example, *cis*-response element ACC(A/T)AA(C/T) is present in promoters from B73 and Mo17 for *CAD6* but is mutated in the *CADa* of Mo17 and correlated with the low expression of this gene (Table 3).

## Conclusions

The genetic diversity of maize is greater than that among Hominidae [79, 80]. Even between two elite inbreds, over one-third of expressed genes differ in expression level. This may explain in part the transgressive segregation observed in recombinant inbred lines derived from B73 and Mo17 [12] and the even larger range of variance in biomass-relevant traits (lignin amount, S:G ratio, glucose and xylose release in saccharification conditions) when the maize Association Panel is examined by Genome-wide association studies (GWAS). Strategies to gain genetic control of biomass structural features therefore need to identify the specific gene networks in each genotype that give rise to a common set of wall polymers and architectures. Pan-genomic characterizations are needed to capture and exploit genetic diversity through identification of ‘core’ genes that are commonly expressed and those that are expressed in a genotype-specific fashion.

As a starting point, we provide here a compendium of cell wall-related genes and their patterns of expression in stems. Considering the extensive development of the maize diversity collections and their comprehensive genotyping [81–83] and their proven utility in genome-wide association [84, 85], this rich resource could be employed to develop and map the collection of regulatory genes required for modulation of developmental networks. Regulatory control of genotype-specific gene networks for secondary wall formation could be a strategy to improve both quantity and quality of lignocellulosic biomass for end-uses of fuel and chemical production.

## Methods

### Maize tissue collection and RNA isolation

The maize (*Zea mays*) Mo17 and B73 lines were grown at the Purdue University Agricultural Center for Research and Education in West Lafayette, IN, or in the Purdue University greenhouses. Greenhouse-grown plants received 16 h per day of 150–250 μmol m^− 2^ s^− 1^ supplemental lighting by metal halide bulbs approximately 1 m from the top of the plants. Field-grown plant materials were harvested from 35 to 63 d after planting, at stages of early and late elongation through deposition of most secondary cell wall biomass, for internodes 2 through 9. A separate collection of internodes 4 through 7, at 49 days after planting in the green house, yielded upper internodes that were elongating and lower internodes that had ceased elongation and exhibited maximal secondary wall formation.

Rind tissues from a minimum of three plants for all samples were excised aseptically from the internodes and immediately plunged in liquid N_2_. Frozen samples were pulverized by mortar and pestle in liquid N_2_. Approximately 2 mg of ground tissue was incubated with 1 mL of ice-cold TRIzol reagent (Invitrogen, Life Technologies) and extracted according to the manufacturer’s directions. Purified RNA was dissolved in 100 μL of diethyl pyrocarbonate-treated Barnstead GenPure (Thermo Fisher Scientific) water and RNA quality and concentration were determined spectrophotometrically. The three RNA samples were pooled for a single RNAseq analysis, and two to three independent isolations were analyzed.

### Lignin, cellulose, and sugar determinations

Lignin was estimated using pyrolysis molecular-beam mass spectroscopy as described in Penning et al. [86]. Briefly, ~ 4 mg of ground, ethanol-washed sample was placed in 80 μL stainless steel cups and pyrolyzed in less than 30 s in a pyrolysis oven at 500 °C with an interface of 350 °C and helium flow of 0.9 L min^− 1^ to transfer samples through a 0.32-cm diameter line at 350 °C into the mass spectrometer. A Merlin data acquisition system gathered mass spectral data from *m/z* 30 to 450. All mass-to-charge ratios were normalized to remove any unequal loading effects and those associated with S or G lignin were added to estimate lignin amounts [86].

Carbohydrates were analyzed as previously described [50]. Briefly, five mg of ground, ethanol-washed samples were hydrolyzed in 1 mL of 2 M trifluoroacetic acid with 0.5 μmol of *myo*-inositol for 90 min at 120 °C. Cellulose and other material was pelleted by centrifugation. The cellulose pellet was washed and suspended in 1 mL of water and cellulose content determined by phenol-sulfuric acid assay [87]. The supernatant fraction was transferred to a clean tube and 1 mL *tert*-butyl alcohol added. The liquids were evaporated under a stream of nitrogen gas. The hydrolyzed sugars were re-suspended in water and alditol acid derivatives were made as previously described [50]. The derivatives were separated into seven components representing the major sugars in plant cell walls by gas-liquid chromatography on an SP-2330 (Supelco, Bellefonte, PA) using a 0.25-mm × 30-m column in a helium flow of 1 mL min^− 1^. Upon an initial hold at 80 °C for 1 min, oven temperatures were raised to 170 °C at 25 °C min^− 1^, then ramped to 240 °C at 5 °C min^− 1^ to 240 °C. Electron-impact mass spectrometry was carried out on a Hewlett-Packard MSD at 70 eV with a source temperature of 250 °C. Ion abundances for each sugar derivative were scaled to mg per mg of sample tissue using the *myo*-inositol internal standard.

### Light and scanning Electron microscopy

One-half-inch-long internode stem sections were cut free-hand then frozen to − 80 °C in Neg 50 frozen section medium (Richard-Allan Scientific, Kalamazoo, MI) on a metal chuck. Stem sections were cross-sectioned to a thickness of 100 μm using a Microm HM550 Cryostat (Richard-Allan Scientific) at − 20 °C. Sections were thawed, the medium washed away with water, and stained using 2% w/v Wiesner’s solution phloroglucinol in equal parts ethanol and 50% HCL (v/v), freshly diluted to 5% in water. Images were taken using a SPOT Insight FireWire 4 Megasample Color Mosaic Camera (SPOT imaging systems, www.spotimaging.com) attached to a Nikon SMZ 1500 stereomicroscope (Nikon Corporation, Kanagawa, Japan) using a 1-11x objective lens set to 10x. Images were captured using SPOT Advanced software version 4.1 (SPOT imaging systems).

SEM imaging was performed on hand-sectioned fresh maize internodes attached to a sample holder by carbon tape with a cryo-adhesive and plunged into liquid nitrogen slush. The samples were placed in a Gatan Alto 2500 pre-chamber, cooled to − 170 °C under vacuum, and sputter-coated for 60 s with platinum. Samples were placed in the cryostage of an FEI Quanta 3D FEG field emission SEM (FEI Company, Hillsboro, OR) for ion-ablating and imaging. Rind areas were ablated by ion milling for one to three minutes in a 65 nA current over a 100 × 300 μm area to remove ice and create a flat surface. Parameters used to view ablation were 30 kV accelerating voltage, 10 mm working distance and 52° tilt. Images were taken at magnifications of 250 to 2500 X with an Everhart-Thornley detector using SEM parameters of 5 kV accelerating voltage, 10 mm working distance, spot 4, and 30 μm aperture.

### Expression analysis

Expression analysis was carried out as previously described [12]. Briefly, pooled total RNA from three biological replicates was subjected to library construction using Illumina’s TruSeq RNA Library Prep Kit and then clustered on a HiSeq 2000 to produce paired-end 100 base sequences. High-quality trimmed sequences were mapped to the Maize B73 sequence V2 from Plant GDB (http://www.plantgdb.org) using Bowtie2 [88], except in instances where the reads mapped to exactly two loci due to the high degree of gene duplication in maize. In these instances, a custom Perl script was used to split the reads between the two loci [12]. An average mapping rate of 80% was achieved over all samples. A separate set of Perl scripts was used to add closest Arabidopsis homolog by nucleotide sequence with description and expect value to the file. One count per million or greater was used as a threshold for the detection of transcript [89, 90]. The RNAseq data are available at NCBI with the following link https://www.ncbi.nlm.nih.gov/sra/PRJNA522448. For hierarchical clustering, Principal Components Analysis (PCA) and slope metric analysis, expression counts were normalized by gene in Genesis version 1.7.7 using the Adjust: Normalize Genes function [91].

### Statistical analyses

Hierarchical clustering was performed using Genesis release 1.7.7 [91]. Gene expression was clustered by average linkage clustering using custom heat-map positive and negative values with gradients between them. The PCA was performed on gene expression by covariance using the values from Genesis in R [92], with the prcomp function of factoextra [93]. Groups were identified either by known association with secondary or primary cell wall biology or by hierarchical clustering. Loadings and a graph of values from the three PCs with the highest correct assignments were saved to a CSV file with the R write.csv command and graphed in Microsoft Excel.

Using a slope-metric algorithm, a custom Perl script was used to identify co-expressed genes most similar to the pattern established for each stage by hierarchical clustering of target cell wall-related genes [3]. The equation to determine the slope metric was as follows:

$${\sum}_{i=1}^{n-1}\mathrm{abs}\left[\left(\mathrm{A}i+1-\mathrm{A}i\right)-\left(\mathrm{X}i+1-\mathrm{X}i\right)\right]$$

where X_*i*_ was the expression of the test gene for the *i*th internode, A_*i*_ was the expression of the bait gene in *i*th internode, X_*i* + 1_ was the expression of the test gene at the next internode in the series, A_*i* + 1_ was the expression of the average of all cell wall-related genes for the stage from hierarchical clustering and PCA at the next internode of the series, and *n* was the number of internodes. The gene set included all genes where at least one internode sample had one RNAseq read per million reads, for a total of 33,232 genes. Gene expression levels in each sample were normalized in Genesis version 1.7.7 prior to applying the slope metric [88]. Standard deviations for each stage using all transcript abundances were calculated, and genes with slope-metric values less than one standard deviation were rank-ordered.

Differential expression analysis was performed in R [93], using the EdgeR package [94], with raw read counts for each internode and replicate-normalized to 20 million reads per tissue, the average of all tissue replicates. Genes with no expression in any internode of greater than 20 reads were removed prior to expression analysis using a custom Perl script. All gene expression was further normalized using the estimate GLM CommonDisp, TrendedDisp, and TagwiseDisp functions and compared by low vs. high expression using the exactTest function in EdgeR with the appropriate design group as follows: For Early and Late pattern, internodes 2, 3, and 8 were highly expressed versus internodes 4, 5, and 9, while the Elongation pattern tested internode 8 versus internode 2, Transitional patterns tested internodes 4 and 5 versus internodes 8 and 9, and the Secondary pattern tested internodes 2 and 3 versus internodes 8 and 9. Gene names, fold changes, *p*-values, and false discovery rates were exported to a CSV file using the write.csv command in R for the topTags command of EdgeR, and n equal to all genes. In Microsoft Excel, genes ranked with false discovery rates of < 0.05 were reported as significant.

### Promoter analysis

Promoter sequences (500 or 1000 bp) from maize or Arabidopsis were found by the Promzea program [35] for cell wall related genes defining each of the five expression groups as over-represented compared to 500 randomly selected genes. Over-represented motifs were reported as WebLogos. These motifs were matched to previously identified promoter motifs using STAMP with AGRIS, PLACE, and AthaMap plant promoter databases [36] and PlantPlan 3.0 [37]. Expect (E) scores based on pairwise alignment of the Promzea promoter to the known plant promoter were calculated, and a WebLogo representation of the known promoter was generated. E values below 10^− 5^ were considered significant.

### Tree building

Phylogenetic trees were constructed as described previously [9]. Briefly, protein-coding sequences for gene families and nucleotide sequences for promoter regions were assembled using the neighbor-joining method for a slow, accurate alignment in ClustalW [95, 96]. The trees were bootstrapped 1000 times, and the number of times that the same clade occurred is indicated on the tree. The trees were visualized using TreeDyn (http://www.treedyn.org) [97].

## Supplementary information

**Additional file 1: Dataset 1.** Cell wall-related genes of maize B73.

**Additional file 2: Table S1.** ‘Housekeeping’ genes with constitutive expression across all developmental stages.

**Additional file 3: Dataset 2.** Genes of the maize B73 genome associated with five expression patterns as determined by slope-metric analysis.

**Additional file 4: Figures S1-S23.** Differential expression in families of genes associated with cell-wall synthesis.

**Additional file 5: Dataset 3.** Comparison of fold-change of gene expression between inbreds B73 and Mo17 in rind tissues of developing internodes of greenhouse-grown plants representing four stages of stem development.

**Additional file 6: Figure S24.** Principal Components Analysis of expression classes defined by hierarchical clustering.

**Additional file 7: Figure S25.** Abundance within classes of genes that best fit the secondary wall pattern defined by slope-metric analysis.

**Additional file 8: Table S2.** Numbers of each of the fifteen motifs identified by PromZea analysis in the promoter regions of genes expressed during the Secondary wall stage of development. Motifs and their classification by STAMP are provided in Fig. 8.

**Additional file 9: Table S3.** Twenty-five genes best fitting the Secondary wall pattern contain the PALBOXA promoter consensus sequence CCGTCC.

**Additional file 10: Figure S26.** Comparison of cellulose, lignin, and sugar accumulation in developing internodes of greenhouse-grown B73 and Mo17.

**Additional file 11: Dataset 4.** Comparison of fold-change of gene expression between inbreds B73 and Mo17 in rind tissues of developing internodes of greenhouse-grown plants representing four stages of stem development.

**Additional file 12: Figures S27-S52.** Comparative expression of maize B73 and Mo 17 gene families during stem development.

**Additional file 13: Table S4.** Fold-change differences in levels of expression of B73 and Mo17 genes in common for both cell wall-related and all genes of elongation and secondary wall stages of stem development.

**Additional file 14: Dataset 5.** Relative expression of the transcriptomes of maize B73 and Mo17 stem development.

## Data Availability

The RNA-seq data are available at NCBI with the following link https://www.ncbi.nlm.nih.gov/sra/PRJNA522448. Our updated maize B73 annotations of cell-wall–related genes are available at Cell Wall Genomics (https://www.maizegdb.org/gbrowse/maize_ v2test?q = Chr1:1..301354135;label = CellWallGenes).

## Abbreviations

4CL: 4-Coumarate CoA Ligase
AGP: Arabinogalactan-protein
AUD: UDP-GlcA Decarboxylase
AXY: Altered Xyloglucan
BAHD: mixed acyl-CoA transferase
C3H: Coumarate 3-Hydrolase
C4H: Cinnamate 4-Hydroxylase
CAD: Cinnamyl Alcohol Dehydrogenase
CCoAOMT: Caffeoyl-CoenzymeA 3-*O*-methyltransferase
CCR: Cinnamoyl CoA reductase
CesA: Cellulose synthase
CNV: Copy-number variation
Csl: Cellulose synthase-like
Exp: Expansin
F5H: Ferulate 5-Hydroxylase
FLA: Fasciclin-like
GALT: Galactosyl Transferase
GATL: GAlacturonosyl Transferase-like
GAUT: Galacturonosyl Transferase
GAX: Glucuronoarabinoxylan
GH: Glycosyl Hydrolase
GME: GDP-Man 3,5-Epimerase
GPI: Glycosylphosphatidylinositol
GT: Glycosyl Transferase
GUX: Glucuronosyl transferase
GWAS: genome-wide association studies
HC: Hierarchical clustering
HCT: Hydroxycinnamoyl-CoA Shikimate/quinate hydroxycinnamoyl Transferase
InDel: Insertion/Deletion
IRX: Irregular Xylem
MLG: Mixed-linkage (1 → 3),(1 → 4)-β-D-glucan
MUCI: Mucilage-related
MUR: Murus (wall-related)
PAGR: Pectin arabinogalactan synthesis-related
PAL: Phenylalanine/tyrosine Ammonia Lyase
PAV: presence-absence variation
PCA: Principal Components Analysis
PGase: Polygalacturonase
PyMBMS: Pyrolysis molecular-beam mass spectroscopy
RG-I: Rhamnogalacturonan-I
RHM: Rhamnose synthase
RNA-seq: high-throughput RNA sequencing
RRT: Rhamnosyl Transferase
RWA: Reduced Wall Acetylation
SEM: Scanning Electron Microscopy
SNP: Single-nucleotide polymorphism
TBL: Trichome-Birefringence-like
UAM: UDP-Ara Mutase
UGD: UDP-Glc Dehydrogenase
UGE: UDP-Glc Epimerase
UXE: UDP-Xylose 4-Epimerase
XTH: Xyloglucan endoTransglucosylase/Hydrolase
XXT: Xyloglucan Xylosyl Transferase
XyG: Xyloglucan
