# Supplementary material for: Expression profiles of cell-wall related genes vary broadly between two common maize inbreds during stem development
Source: BMC Genomics. 2019 Oct 29;20:785. doi: 10.1186/s12864-019-6117-z (PMC6819468; doi:10.1186/s12864-019-6117-z)
Supplement: Supplementary file 2 — Additional file 2: Table S1. ‘Housekeeping’ genes with constitutive expression across all developmental stages. [file 12864_2019_6117_MOESM2_ESM.pdf]

Table S1. 'Housekeeping' maize genes with constitutive expression across all developmental stages of field grown maize stem development.[1]

Gene Designator Maize GDB v.2/v.3	Description
AC203173.3_FG004	Elongation factor2
GRMZM2G007721	Sterol 3-β-glucosyltransferase
GRMZM2G018103	Membrane protein PB1A10.07c
GRMZM2G020801	Aconitate hydratase, cytoplasmic
GRMZM2G027378	Ubiquitin-conjugating enzyme
GRMZM2G038401	Cell division protease ftsH homolog8, mitochondrial
GRMZM2G048371	Enolase
GRMZM2G059015	Nucleoporin autopeptidase family protein
GRMZM2G064475	Centromere/kinetochore protein zw10
GRMZM2G067176	Molybdenum cofactor biosynthesis protein
GRMZM2G090887	Splicing endonuclease positive effector sen1
GRMZM2G102471	Ubiquitin carrier protein
GRMZM2G109383	Phosphoglucomutase, cytoplasmic1
GRMZM2G111238	Conserved gene of unknown function
GRMZM2G117100	Chromo domain-containing protein LHP1
GRMZM2G128613	Conserved gene of unknown function
GRMZM2G139419	OJ000114_01.14 protein
GRMZM2G164418	Ubiquitin carboxyl-terminal hydrolase
GRMZM2G166694	Cullin
GRMZM2G179097	Cyclin-dependent kinase G-2
GRMZM2G334457	SIN3 component, histone deacetylase complex
GRMZM2G393334	Folylpolyglutamate synthase
GRMZM2G421857	Vacuolar ATP synthase catalytic subunit A
GRMZM2G425377	LEUNIG

[1]Housekeeping genes assembled by Sekhon RS, Lin H, Childs KL, Hansey CN, Buell CR, de Leon N, Kaeppler SM. (2011) Genome-wide atlas of transcription during maize development. Plant J. 66, 553–63.
