# Supplementary material for: Expression profiles of cell-wall related genes vary broadly between two common maize inbreds during stem development
Source: BMC Genomics. 2019 Oct 29;20:785. doi: 10.1186/s12864-019-6117-z (PMC6819468; doi:10.1186/s12864-019-6117-z)
Supplement: Supplementary file 4 — Additional file 4: Figures S1-S23. Differential expression in families of genes associated with cell-wall synthesis. [file 12864_2019_6117_MOESM4_ESM.pdf]

Additional file 4: Figures S1-S23. Differential expression in gene families associated with cell-wall biosynthesis.

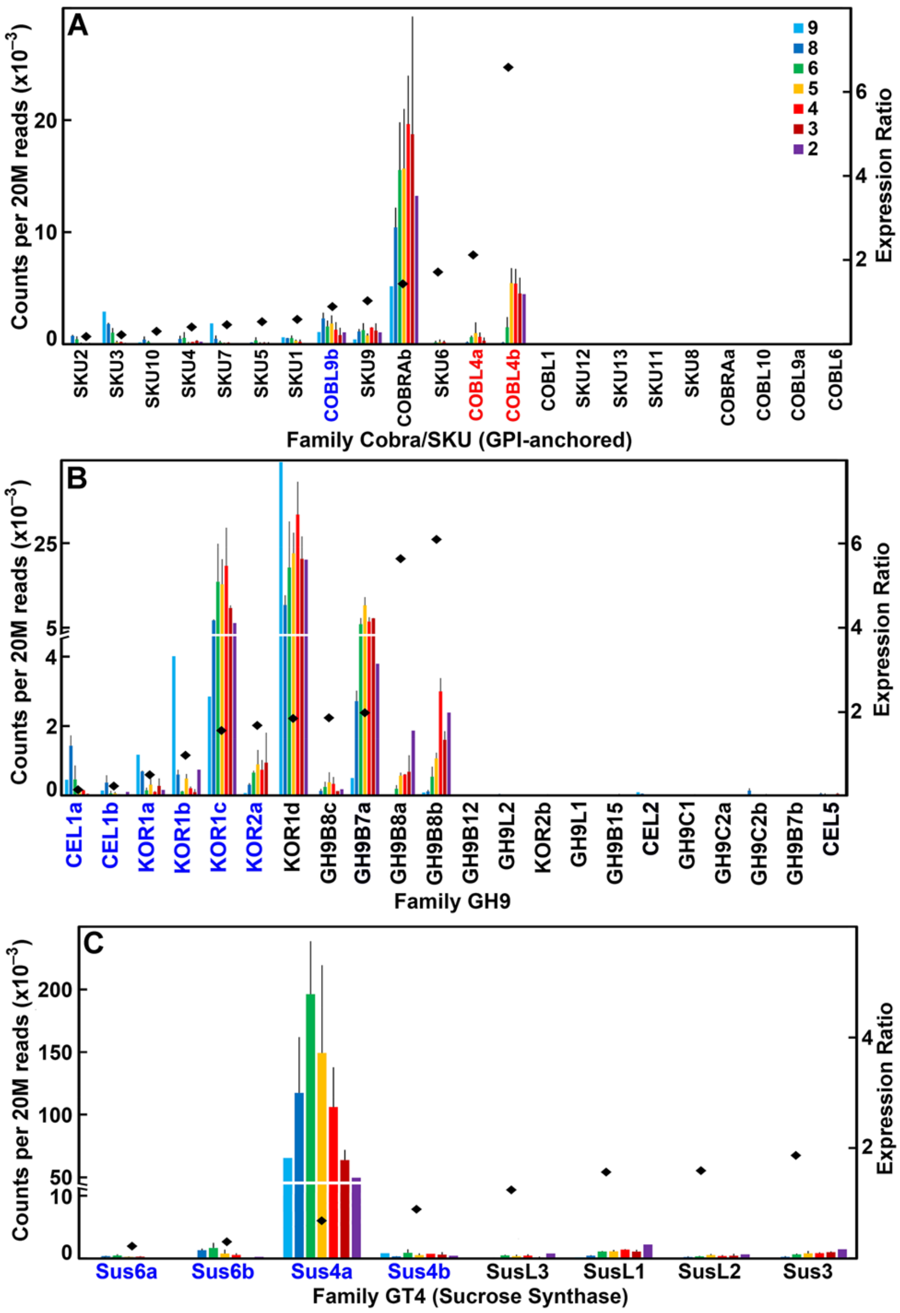

Figure S1. Differential expression in gene families associated with cellulose synthesis. Transcript levels in rind tissues from Internodes 9 through 2 were normalized and compared as counts per 20M reads. Values are the mean ± variance or S.D. of two or three independent rind collections, respectively. Genes with expression ≥ 500 reads were ordered by their ratio of expression (diamonds) in secondary cell-wall-forming tissues (Internodes 5 through 3) to elongating tissue (Internodes 9 and 8)(see Additional file 3: Dataset 1 for expression levels). Blue text indicates the closest Arabidopsis homolog to the maize gene is similarly expressed constitutively or in elongating rind tissues, and red text indicates that the closest Arabidopsis homolog to the maize gene is similarly expressed in secondary cell-wall-forming tissues. **A**. Families COBRA and COBRA-like, and SKU. **B**. Family KORRIGAN and related GH9 glycan hydrolases. **C**. Family GT4, sucrose synthases.

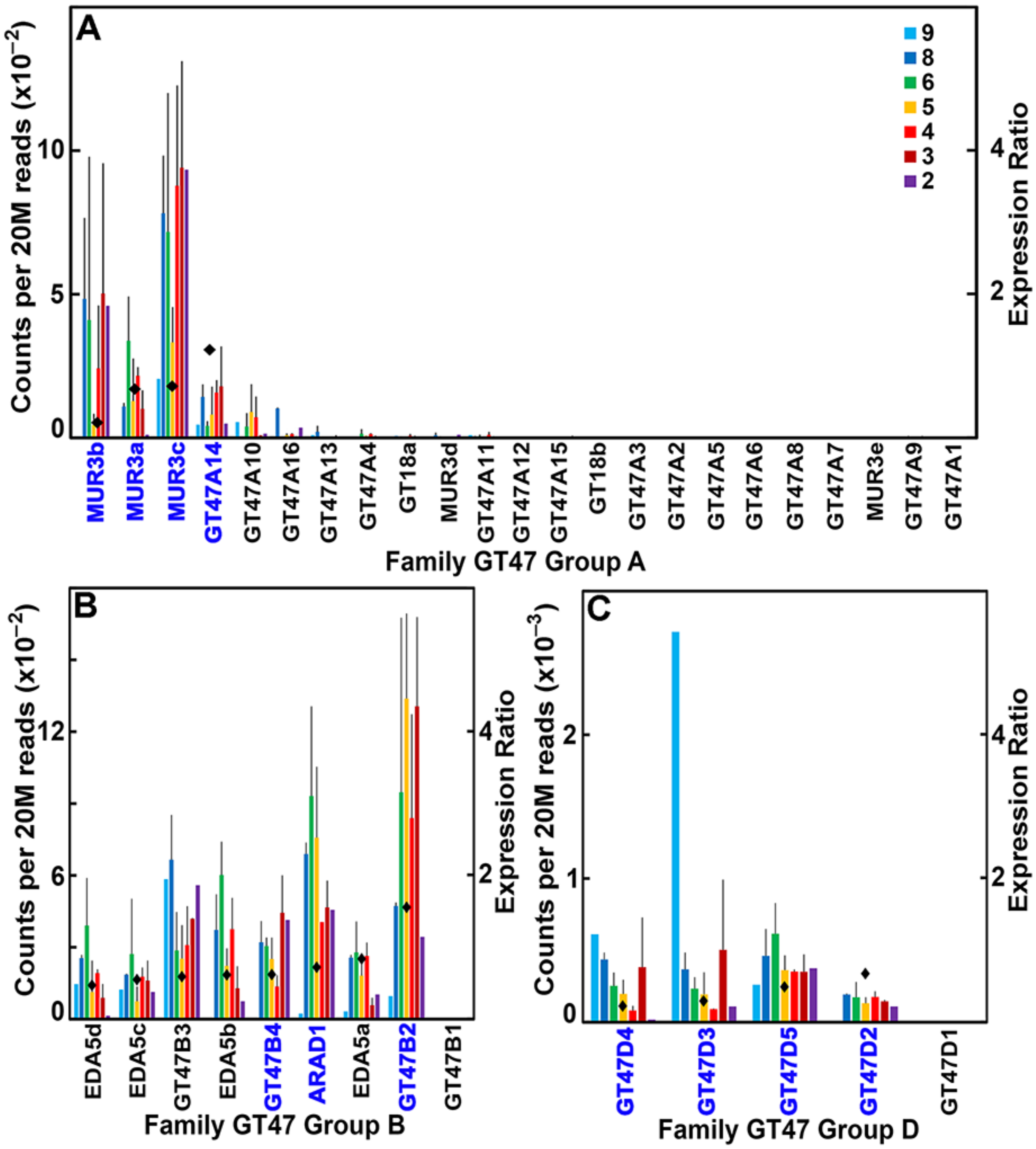

Figure S2. Differential expression within subgroups of gene families of GT47 inverting glycosyl transferases. Expression ratios and potential Arabidopsis orthologs were determined as described in the legend of Figure S1. **A**. Family GT47A. **B**. Family GT47B. **C.** Family GT47D.

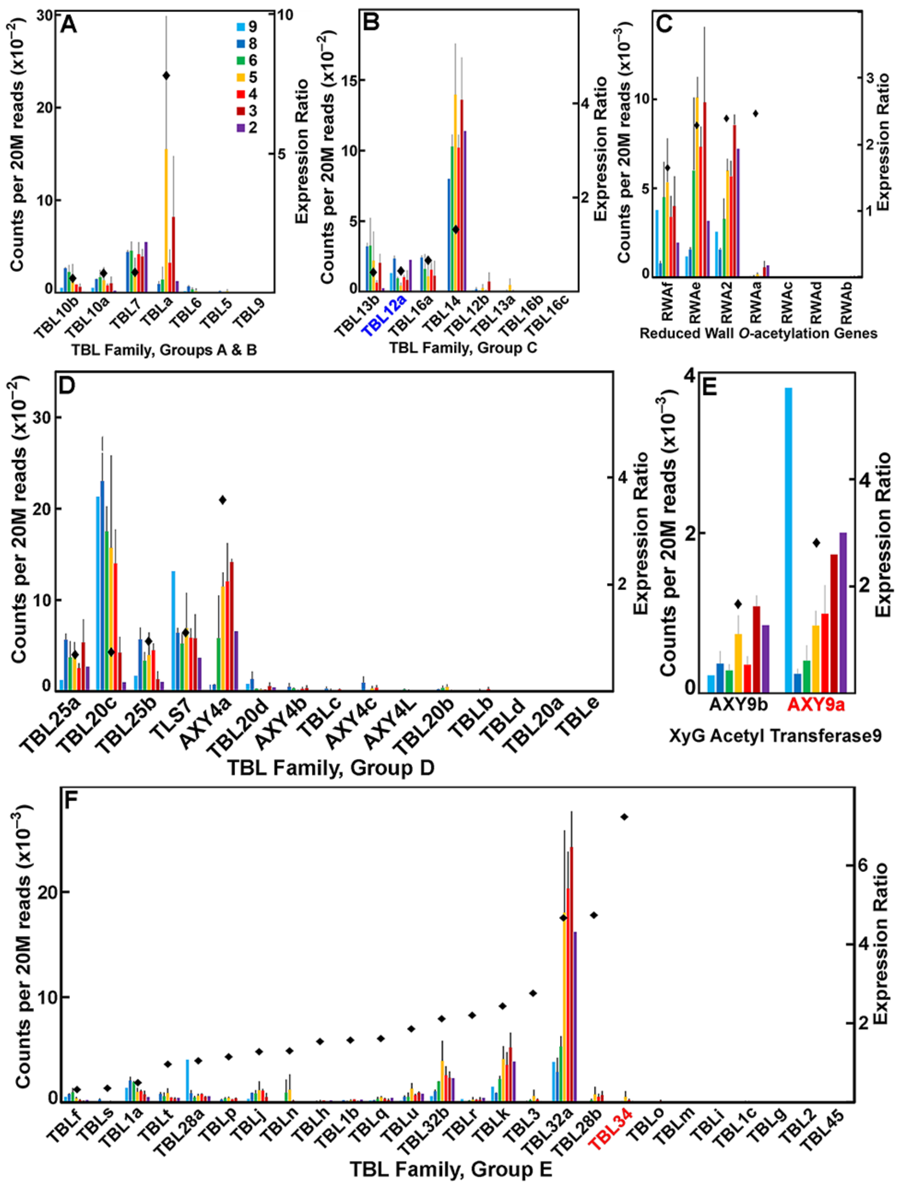

Figure S3. Differential expression in gene families of xylan acetylating enzymes. Expression ratios and potential Arabidopsis orthologs were determined as described in the legend of Figure S1. **A**. Family Trichome Birefringence-like (TBL) acetyl-transferases, Groups A and B. **B.** Family TBL, Group C. **C.** The Reduced Wall Acetylation (RWA) gene family. **D.** Family TBL, Group D. **E.** Family Xyloglucan (XyG) Acetyl transferases. **F.** Family TBL, Group E.

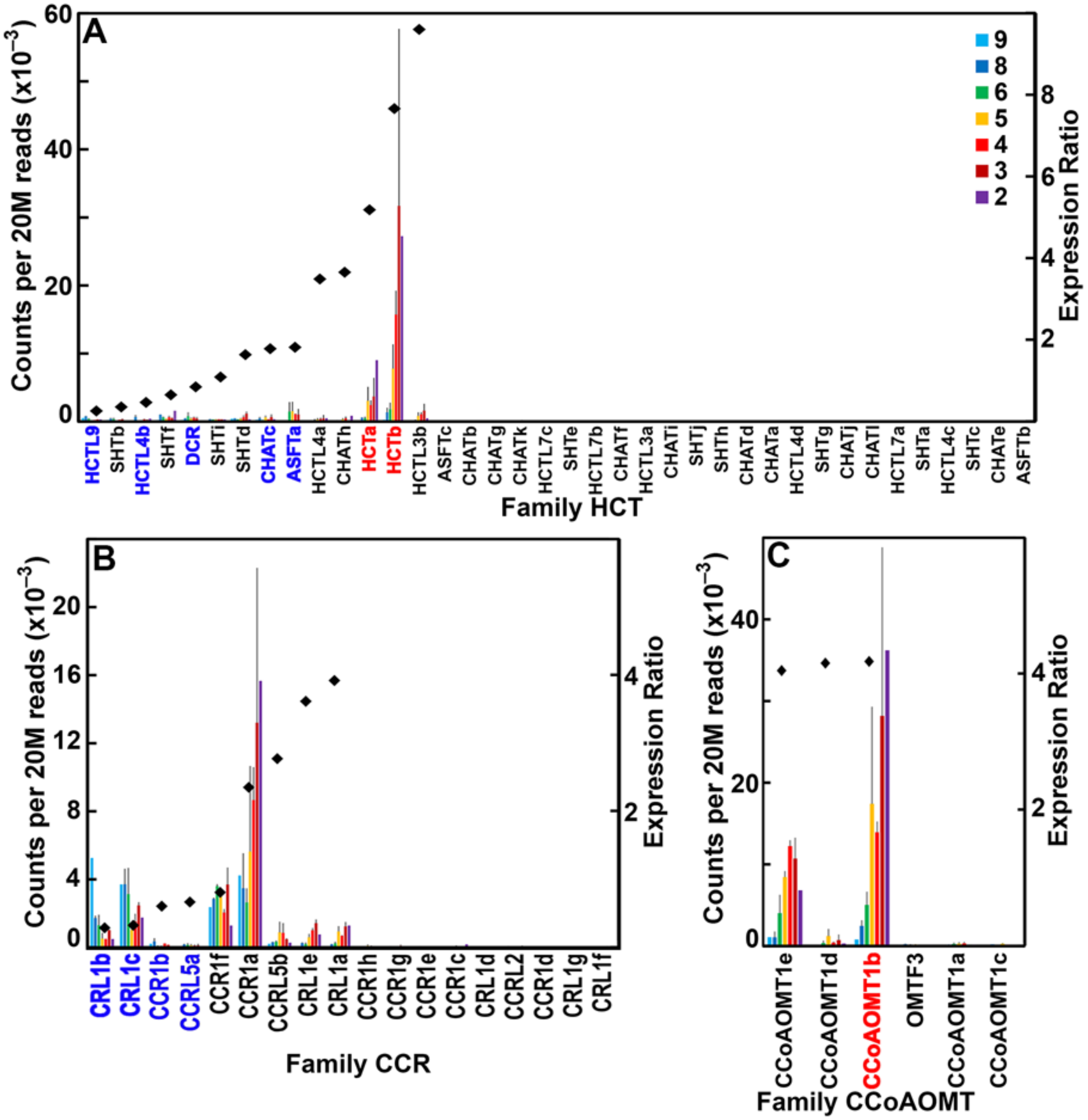

Figure S4. Differential expression in gene families of monolignol synthesis. Expression ratios and potential Arabidopsis orthologs were determined as described in the legend of Figure S1. **A.** Family HCT, Hydroxycinnamoyl-CoA:Shikimate Hydroxycinnamoyl Transferases. **B.** Family CCR, Cinnamoyl-CoA reductases. **C.** Family CCoAOMT, Caffeoyl-CoA *O*-methyltransferases. **D.** Family COMT, Caffeic acid *O*-methyltransferases.

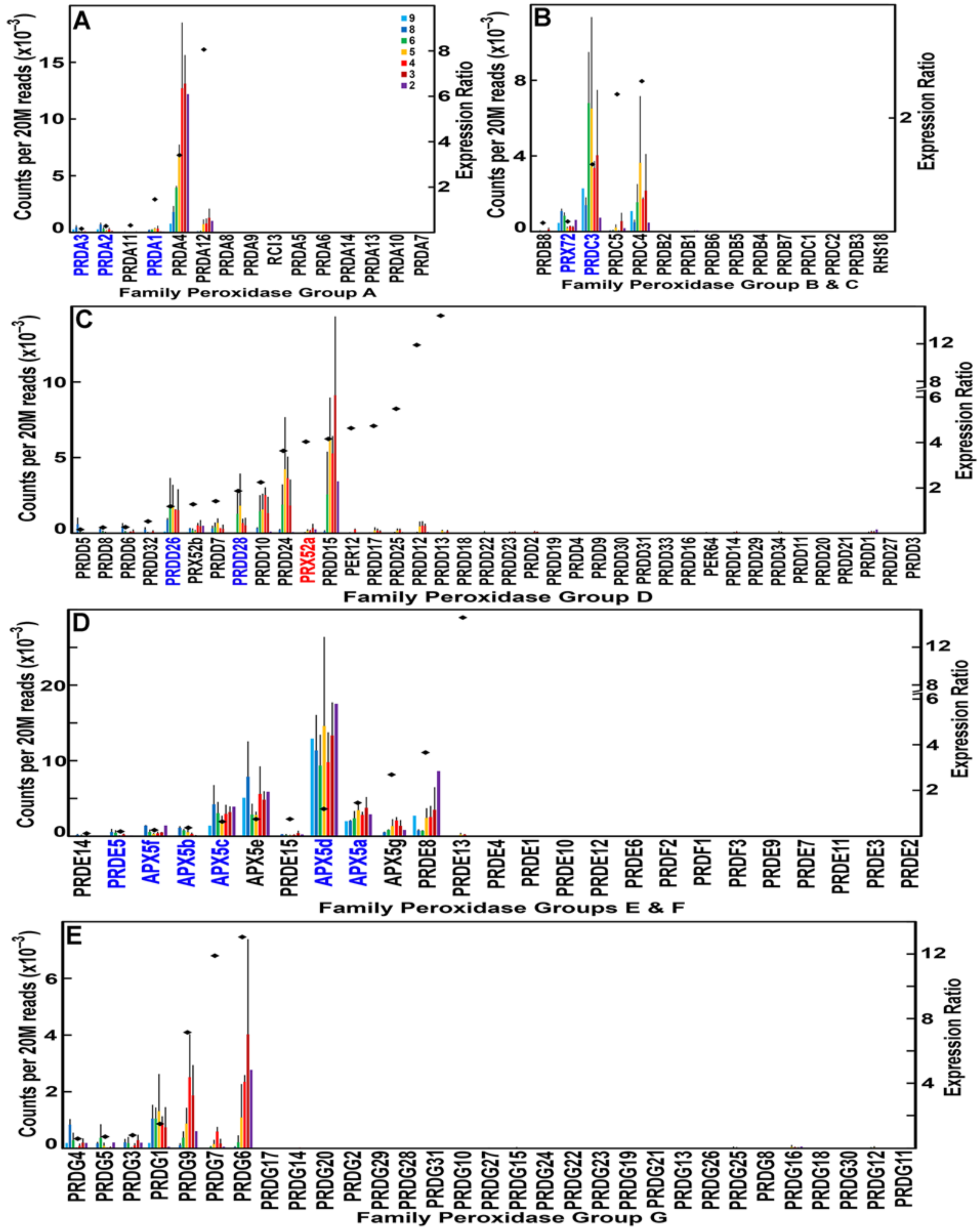

Figure S5. Differential expression in genes of the peroxidase superfamily. Expression ratios and potential Arabidopsis orthologs were determined as described in the legend of Figure S1. **A**. Family Peroxidase, subgroup A. **B**. Family Peroxidase, subgroups B and C. **C**. Family Peroxidase, subgroups D. **D.** Family Peroxidase, subgroups E and F. **E.** Family Peroxidase, subgroup G.

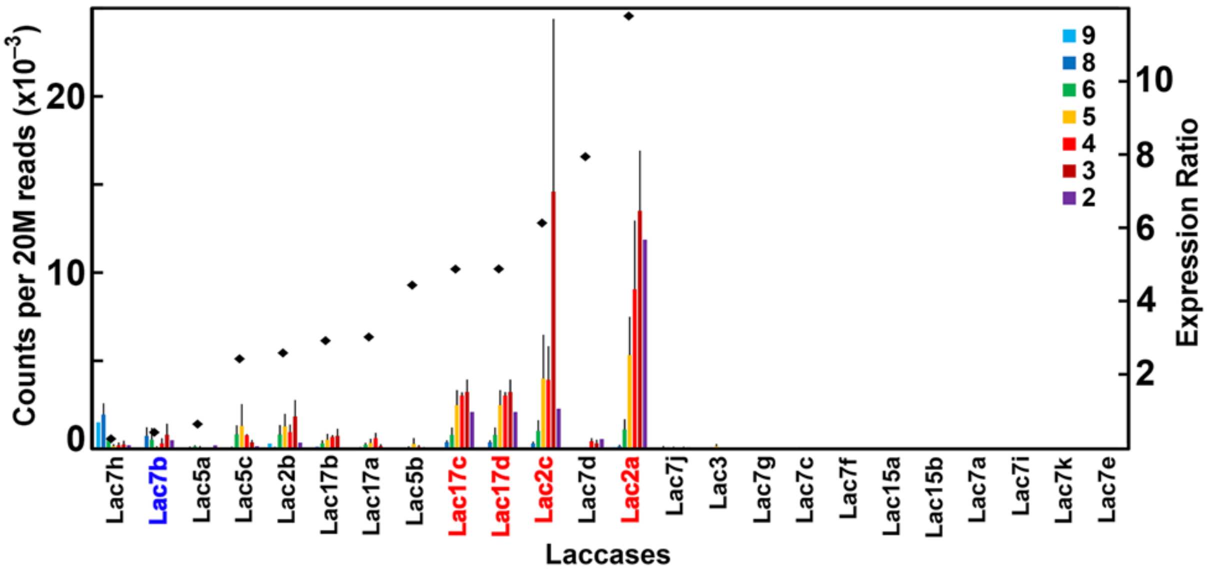

Figure S6. Differential expression in genes of the laccase family. Expression ratios and potential Arabidopsis orthologs were determined as described in the legend of Figure S1.

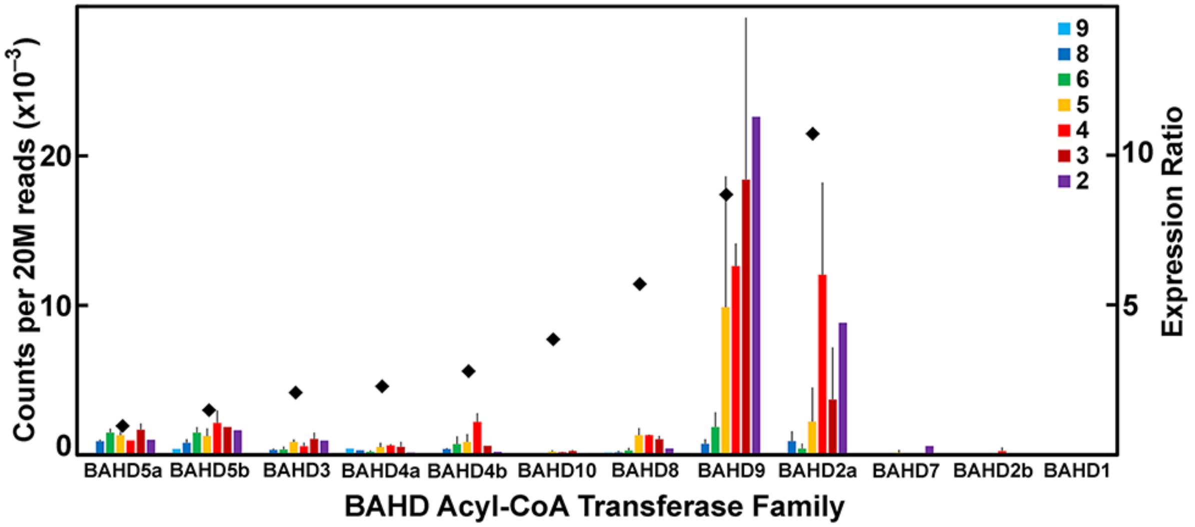

Figure S7. Differential expression in the BAHD gene family of Acyl-CoA transferases. Expression ratios were determined as described in the legend of Figure S1.

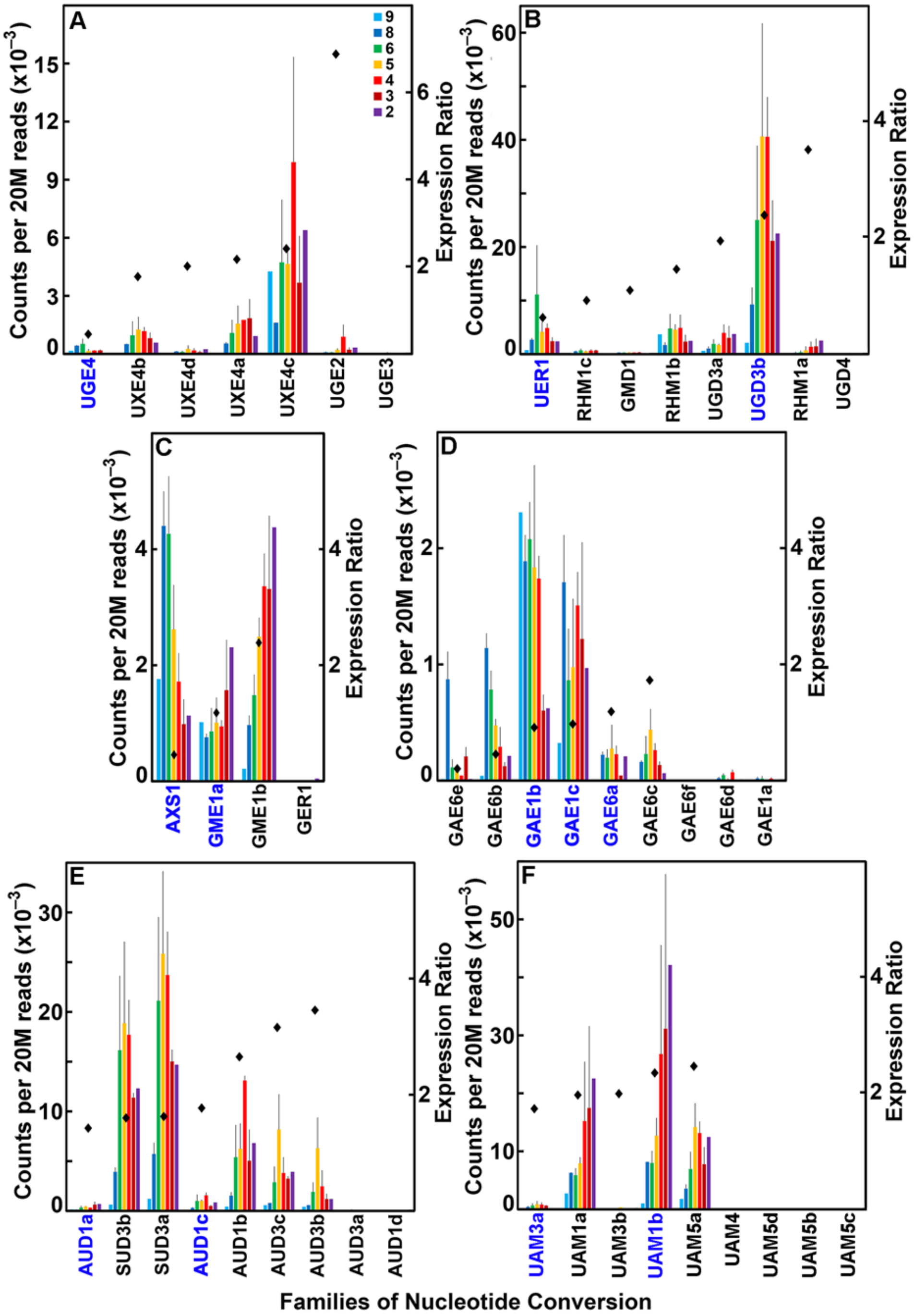

Figure S8. Differential expression in families associated with nucleotide-sugar interconversion. Expression ratios and potential Arabidopsis orthologs were determined as described in the legend of Figure S1. **A.** Family UXE, UDP-D-Xylose 4-epimerases; and Family UGE, UDP-D-Glucose 4-epimerases. **B**. Family UER, UDP-4-keto-6-deoxy-D-Glucose 3,5-epimerase-4-reductases; Family GMD, GDP-D-Mannose 4,6-dehydratases; Family RHM, UDP-L-Rhamnose synthases; and Family UGD, UDP-D-glucose dehydrogenases. **C**. Family AXS, UDP-D-Apiose/UDP-D-xylose synthases; Family GME, GDP-D-Mannose 3,5-epimerase; and Family GER, GDP-4-keto-6-deoxy-D-Mannose 3,5-epimerase-4-reductases. **D.** Family GAE, UDP-D-Glucuronate 4-epimerases. **E.** Family AUD, membrane-anchored UDP-D-Glucuronate decarboxylase; and SUD, soluble UDP-D-Glucuronate decarboxylase. **F**. Family GT75, RGP (UAM), Reversibly glycosylated proteins (UDP-L-Arabinose mutases).

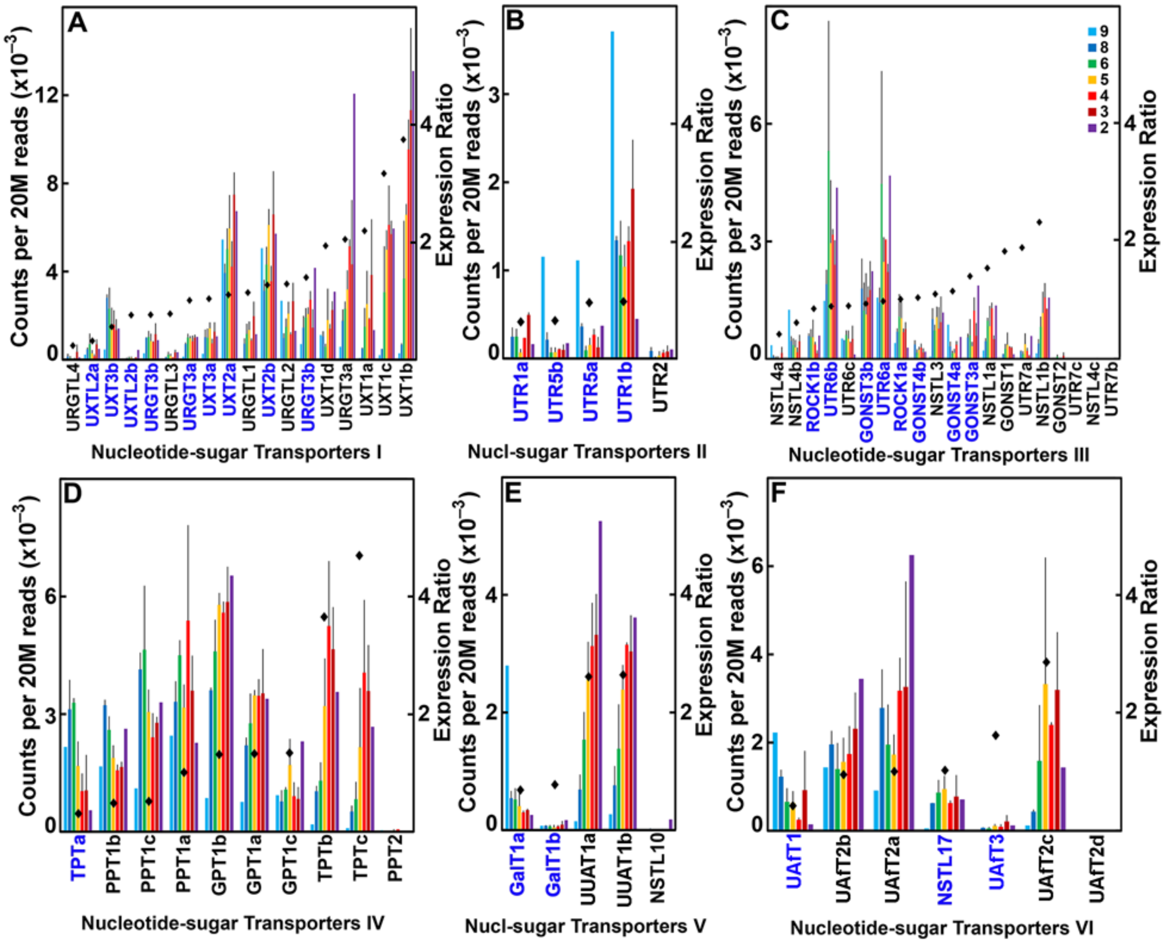

Figure S9. Differential expression in gene families associated with nucleotide-sugar transport. Expression ratios and potential Arabidopsis orthologs were determined as described in the legend of Figure S1. **A.** Group I transporters, **B.** Group II transporters, **C.** Group III transporters, **D.** Group IV transporters, **E.** Group V transporters, **F.** Group VI transporters.

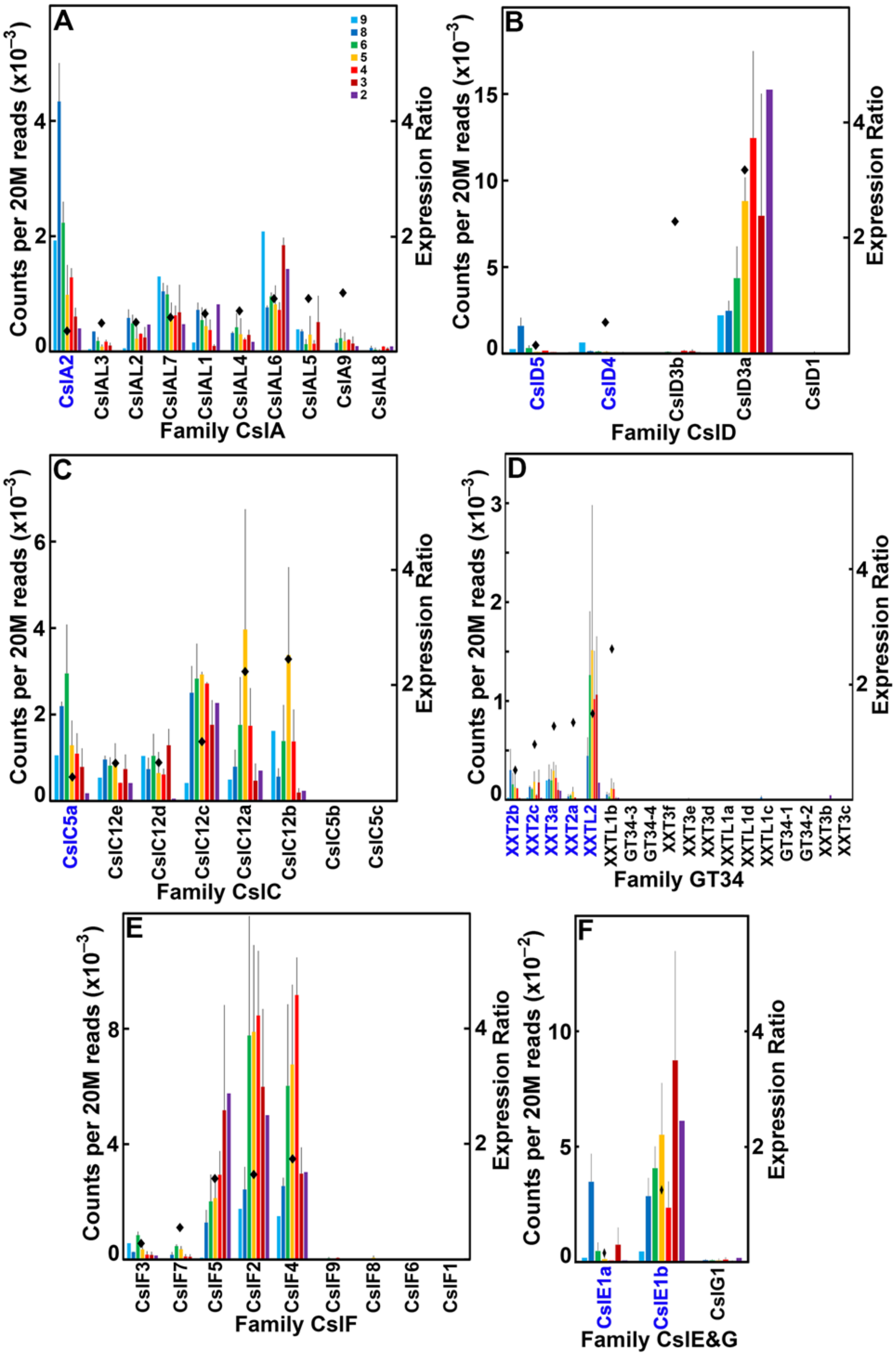

Figure S10. Differential expression in gene of families associated with non-cellulosic glycan synthesis. Expression ratios and potential Arabidopsis orthologs were determined as described in the legend of Figure S1. **A.** Family CslA, (1→4)-β-D-Mannan and Glucomannan synthases. **B.** Family CslD, (1→4)-β-D-Glucan synthases. **C.** Family CslC, putative Xyloglucan (1→4)-β-D-glucan backbone synthases. **D.** Family GT34, Xyloglucan xylosyl transferases (XXTs). **E.** Family CslF, Mixed-linkage (1→3),(1→4)-β-D-glucan synthases. **F.** Family CslE & CslG, putative (1→4)-β-D-Glycan synthases.

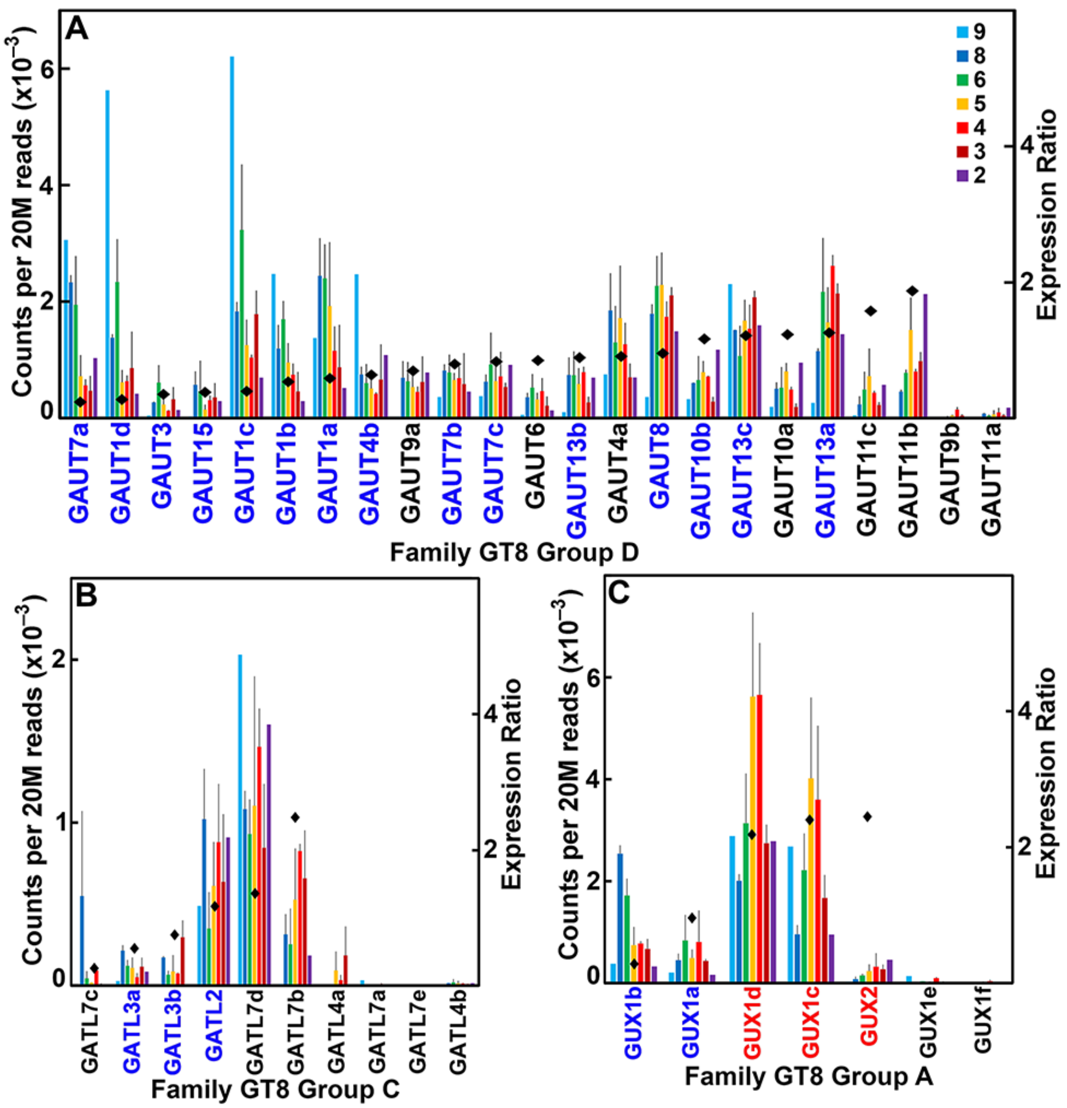

Figure S11. Differential expression of genes within subgroups of Family GT8 retaining glycosyl transferases. Expression ratios and potential Arabidopsis orthologs were determined as described in the legend of Figure S1. **A.** Family GT8D GAUT, pectin UDP-D-GalA transferases. **B.** Family GT8C GATL, pectin UDP-D-GalA transferase-like, **C.** Family GT8A GUX, UDP-GlcA transferases.

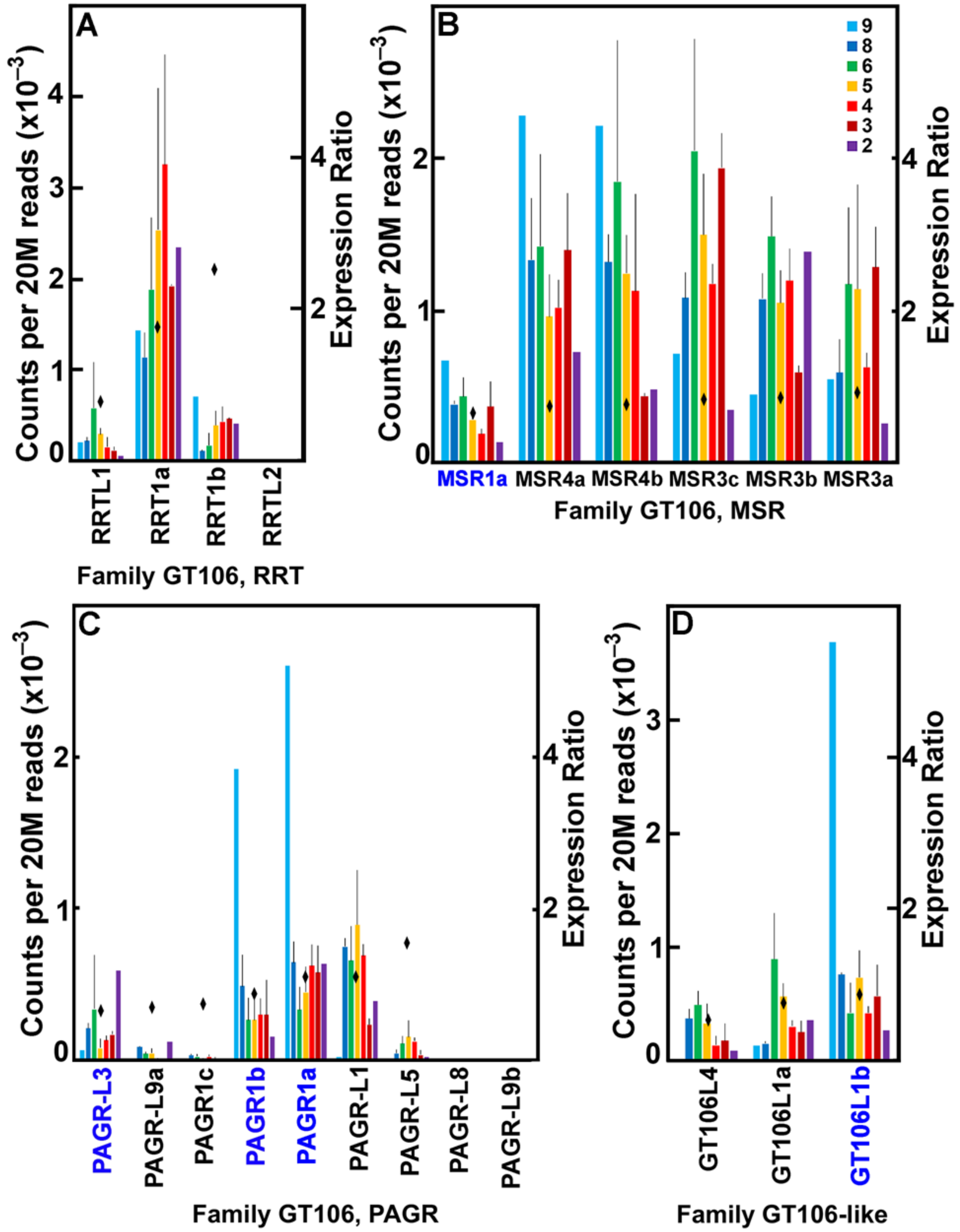

Figure S12. Differential expression of genes within subgroups of Family GT106 inverting glycosyl transferases. Expression ratios and potential Arabidopsis orthologs were determined as described in the legend of Figure S1. **A.** Family GT106, subgroup A of pectin-related Rhamnosyl transferases. **B.** Family GT106, subgroup B of Mannosyl transferases. **C.** Family GT106, subgroup C of pectin type I Arabinogalactan-related transferases. **D.** Family GT106-like, subgroup D.

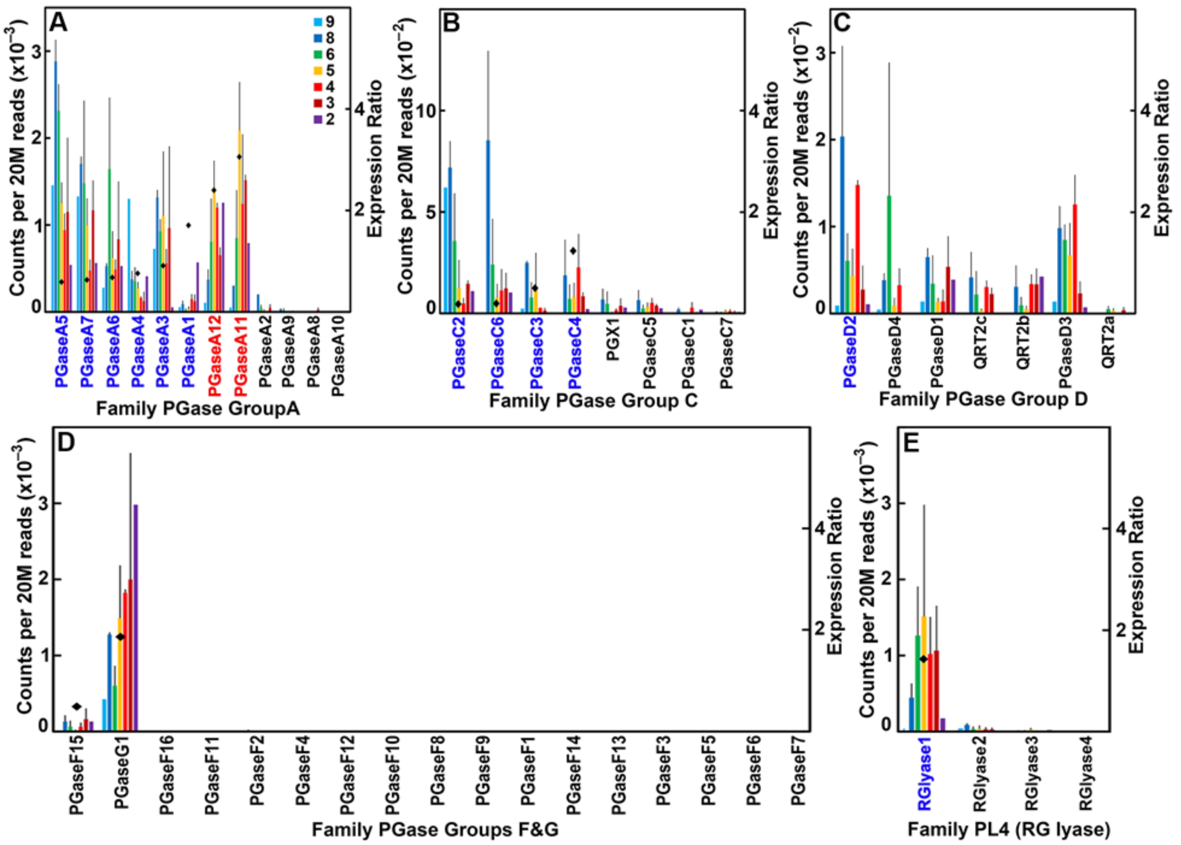

Figure S13. Differential expression of genes in the GH28 polygalacturonase superfamily and PL4 RG lyase family. Expression ratios and potential Arabidopsis orthologs were determined as described in the legend of Figure S1. **A**. Family Polygalacturonase, subgroup A. **B**. Family Polygalacturonase, subgroup C. **C**. Family Polygalacturonase, subgroup D. **D**. Family Polygalacturonase, subgroups F and G. **E**. Family PL4, Rhamnogalacturonan-I lyases.

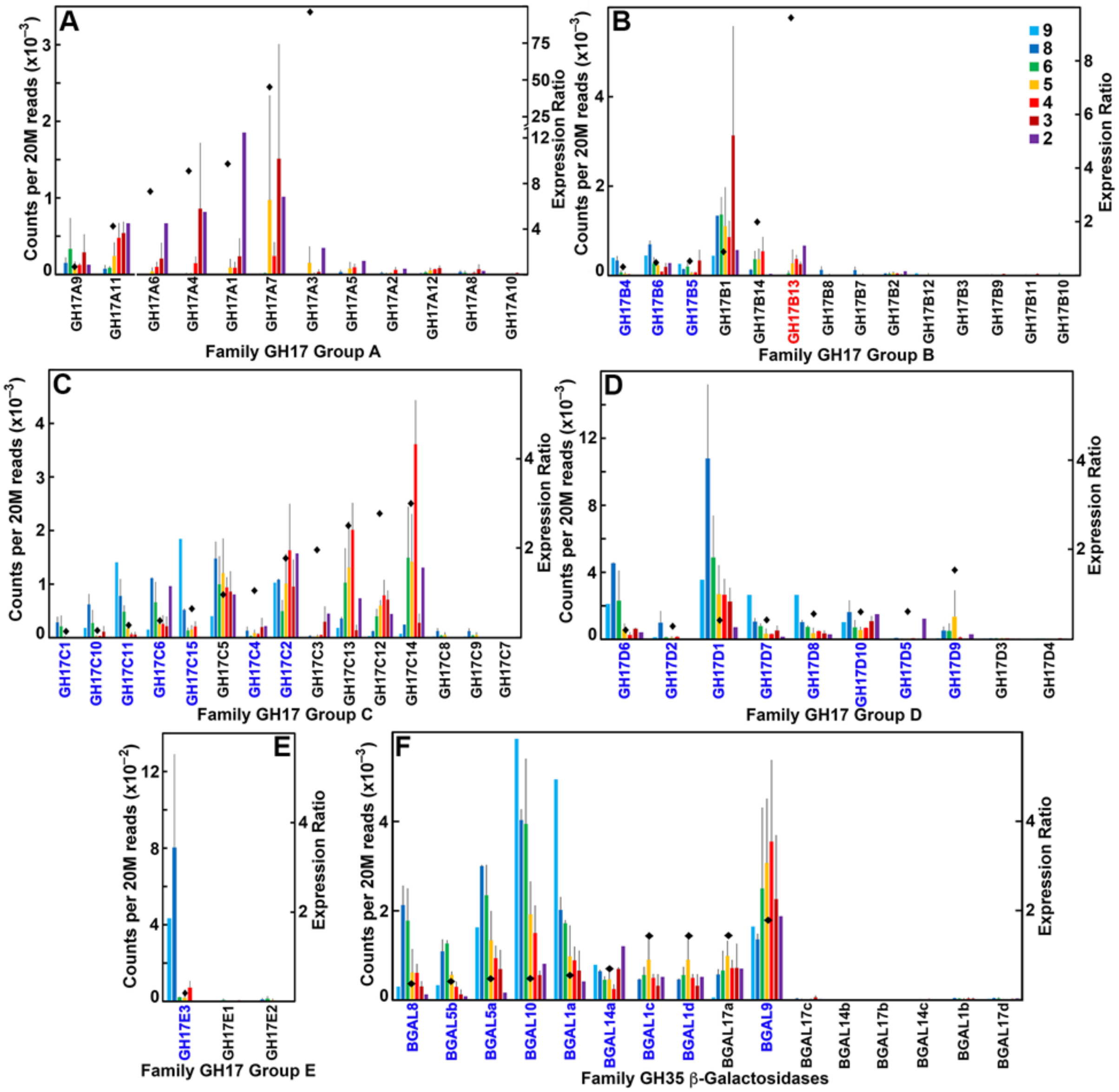

Figure S14. Differential expression within subgroups of families associated with (1→3)-β-D-glucan (callose) hydrolysis and β-galactosidases. Expression ratios and potential Arabidopsis orthologs were determined as described in the legend of Figure S1. **A.** Family GH17 Group A. **B.** Family GH17 Group B. **C.** Family GH17 Group C. **D.** Family GH17 Group D. **E.** Family GH17 Group E. **F.** Family GH35 β-galactosidases.

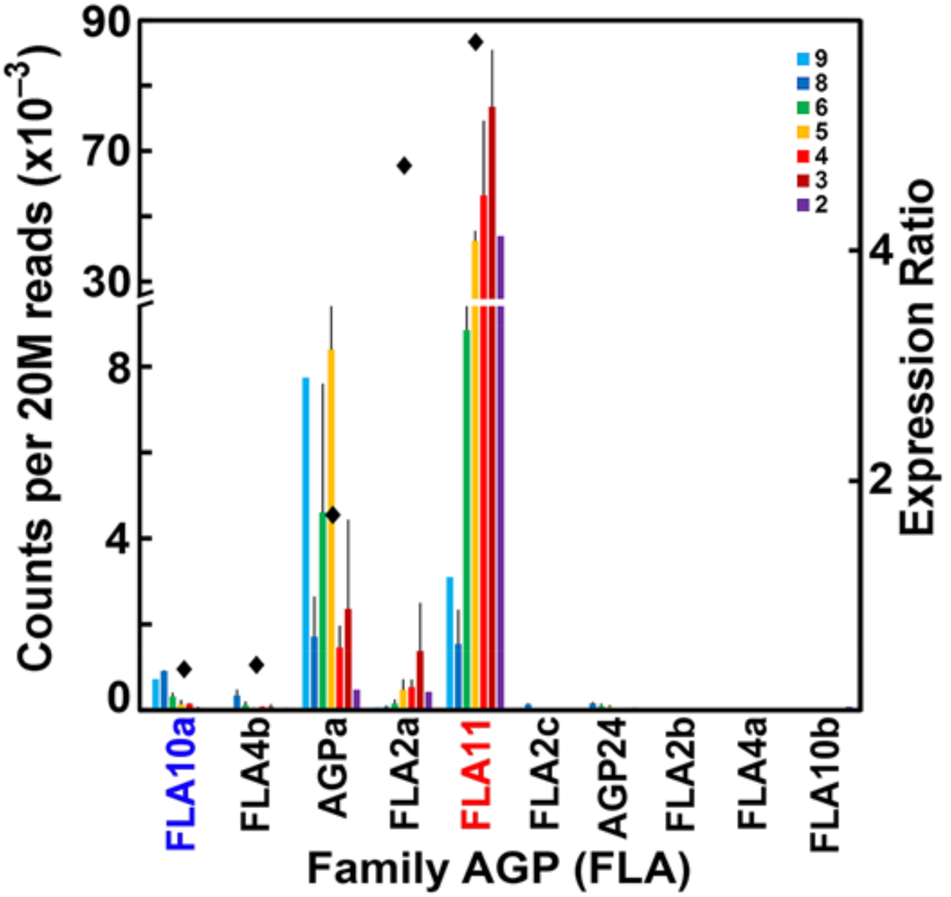

Figure S15. Differential expression of genes within subgroups of the Fasciclin-like (FLA) gene family of core Arabinogalactan-Proteins (AGPs). Expression ratios and potential Arabidopsis orthologs were determined as described in the legend of Figure S1.

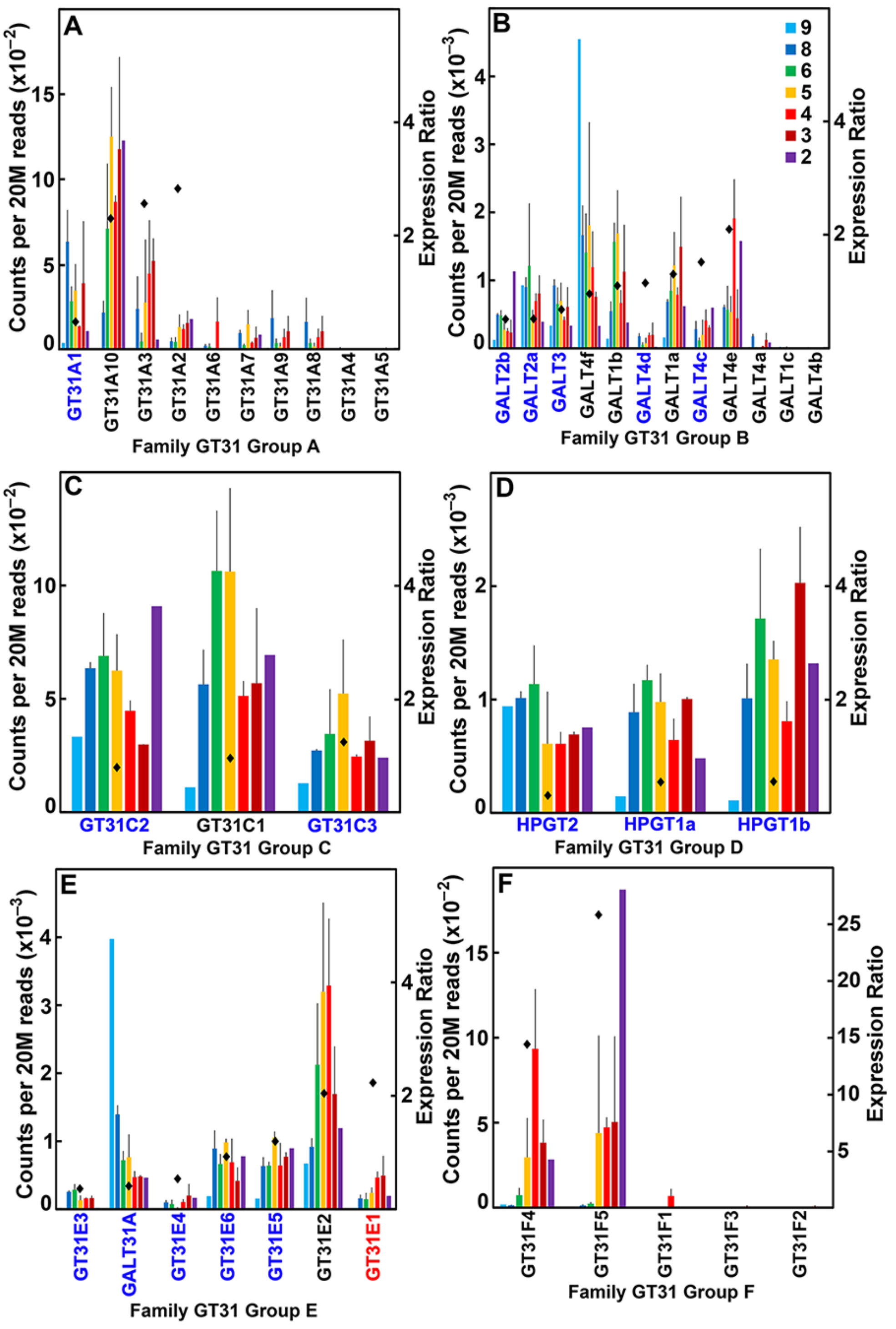

Figure S16. Differential expression of genes within subgroups of Family GT31 glycosyl transferases. Expression ratios and potential Arabidopsis orthologs were determined as described in the legend of Figure S1. **A.** GT31 subgroup A. **B.** Family GT31, subgroup B, galactosyl transferases. **C.** Family GT31, subgroup C. **D.** Family GT31, subgroup D, Hyp *O*-galactosyltransferases. **E.** Family GT31, subgroup E. **F.** Family GT31, subgroup F.

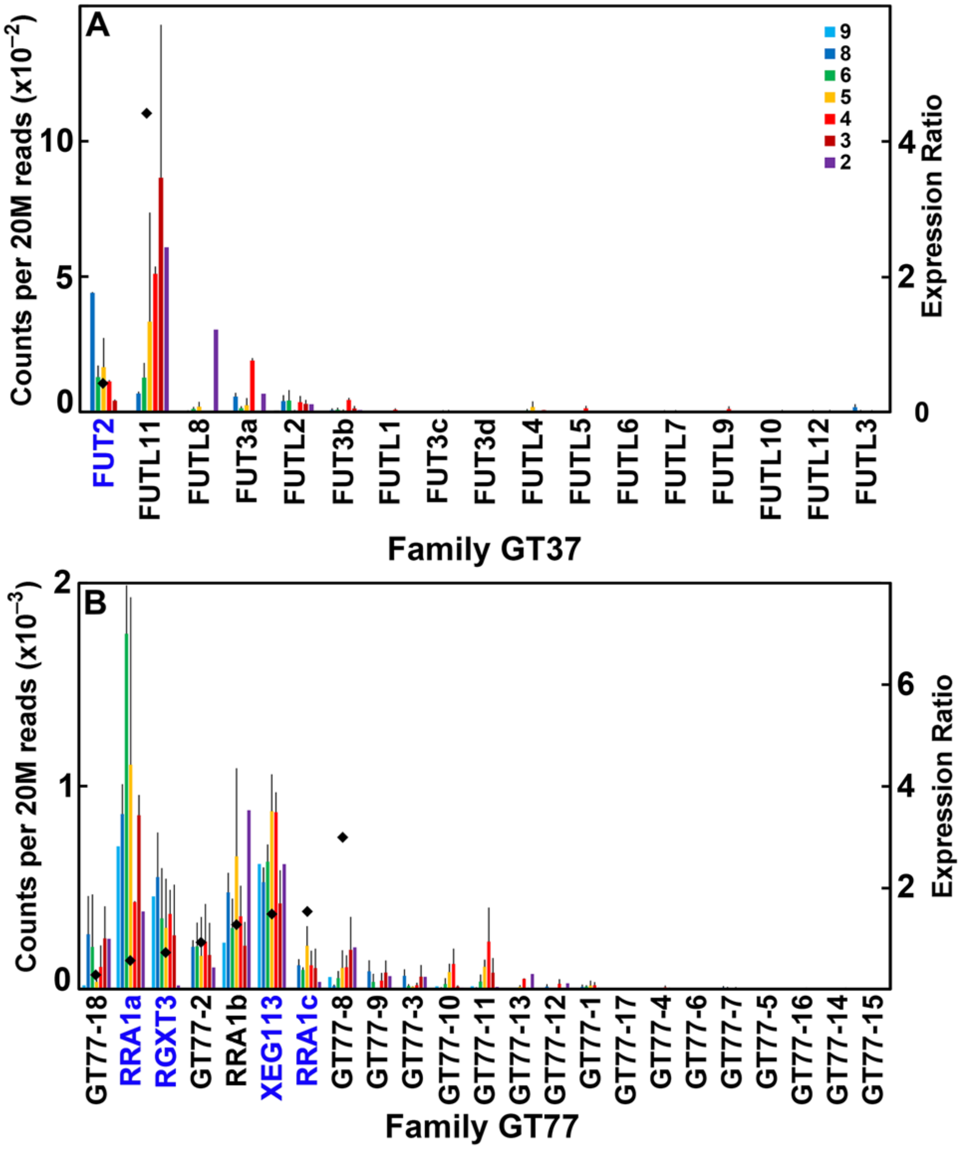

Figure S17. Differential expression of genes within subgroups of Family GT37 and GT77 glycosyl transferases. Expression ratios and potential Arabidopsis orthologs were determined as described in the legend of Figure S1. **A.** Family GT37 FUT, GDP-L-fucosyl transferases. **B.** Family GT77, α-xylosyltransferases, α-1,3-galactosyltransferases, arabinosyl transferases.

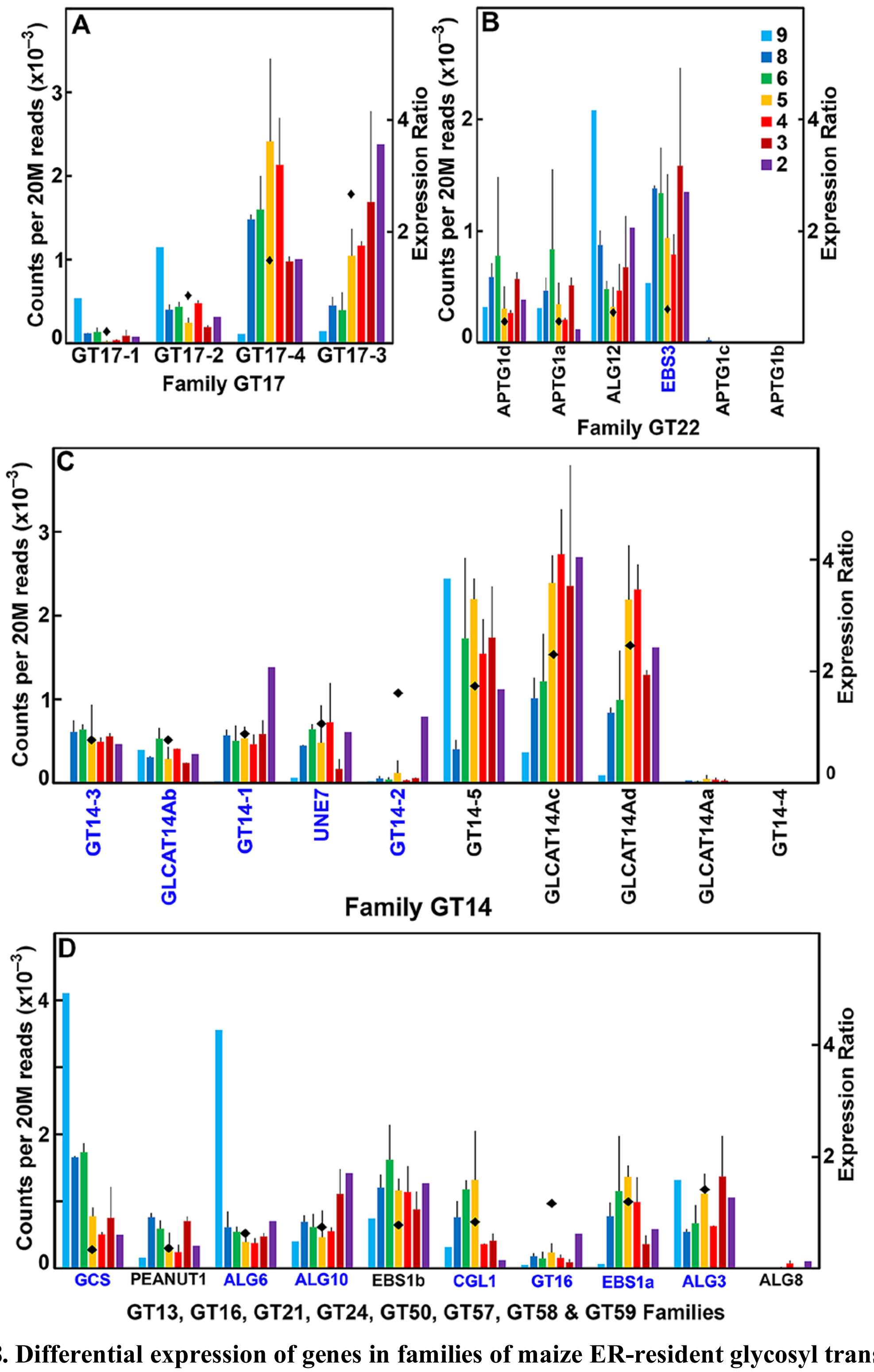

Figure S18. Differential expression of genes in families of maize ER-resident glycosyl transferases. Expression ratios and potential Arabidopsis orthologs were determined as described in the legend of Figure S1. **A.** Family GT17 β-1,4-mannosyl-glycoprotein, β-1,4-*N*-acetylglucosaminyltransferases and related proteins. **B.** Family GT22 Dol-P-Man: $Man_6GlcNAc_2$-PP-Dol α-1,2-mannosyltransferases and related proteins. **C**. Family GT14 β-1,3-galactosyl-*O*-glycosyl-glycoprotein β-1,6-*N*-acetylglucosaminyltransferases and related proteins. **D**. Members of eight additional families of GTs.

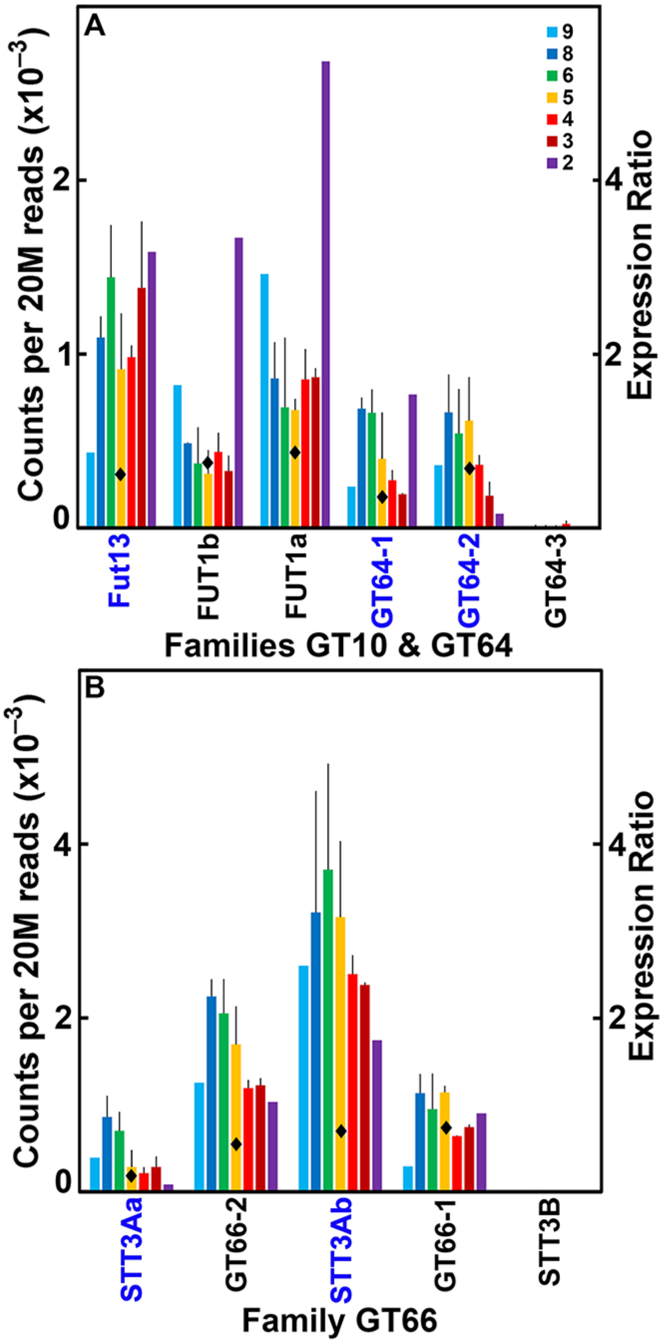

Figure S19. Differential expression of genes in families of maize Golgi-resident glycosyl transferases. Expression ratios and potential Arabidopsis orthologs were determined as described in the legend of Figure S1. **A.** Family GT10 galactoside α-1,3/1,4-L-fucosyltransferases and related proteins, and GT64 heparan α-*N*-acetylhexosaminyl-transferases and related proteins. **B**. Family GT66 dolichyl-diphospho-oligosaccharide-protein glycosyltransferases and related proteins.

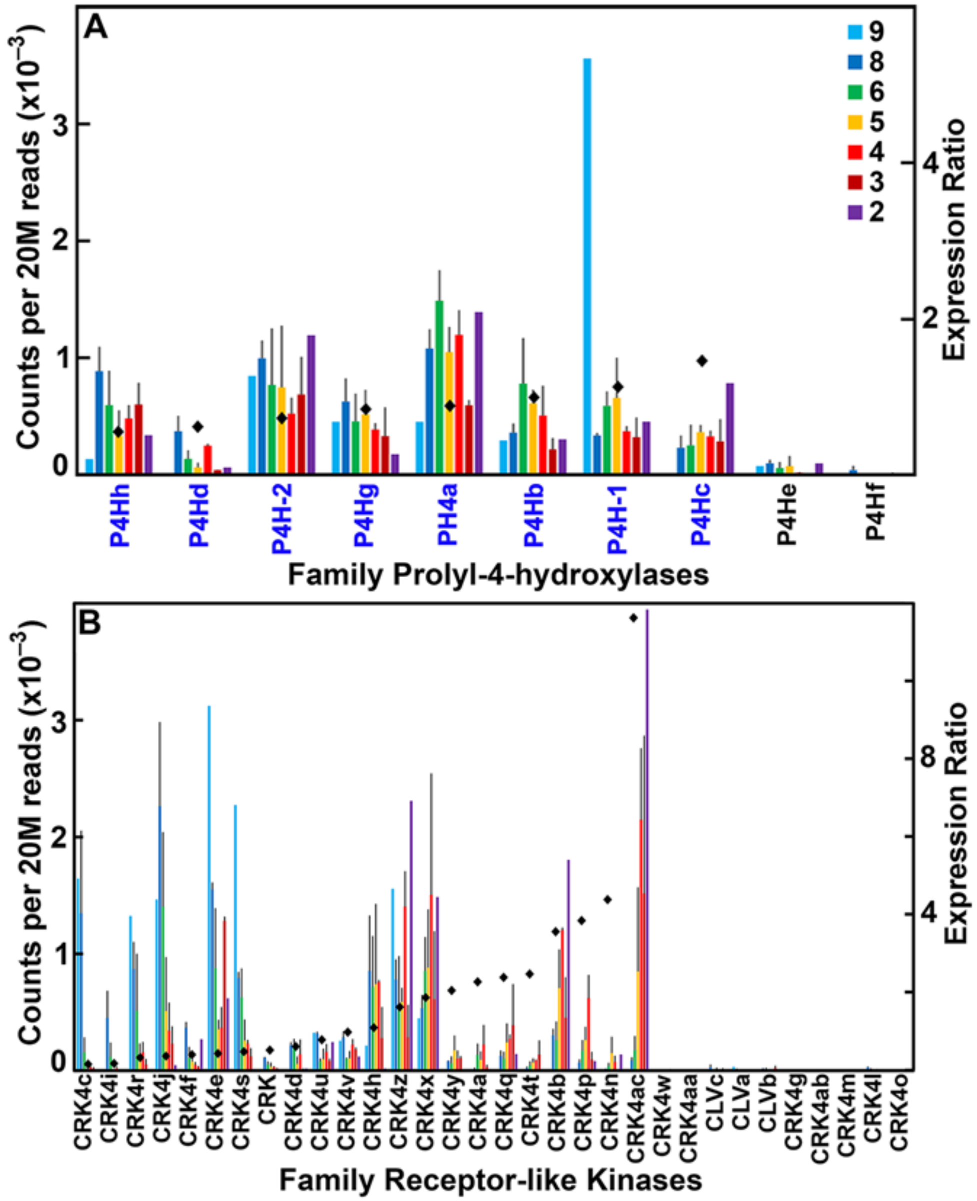

Figure S20. Differential expression of genes in families of maize signaling and secretion-associated proteins. Expression ratios and potential Arabidopsis orthologs were determined as described in the legend of Figure S1. **A.** Prolyl-4-hydroxylases involved in hydroxyproline synthesis. **B.** Receptor-like kinases.

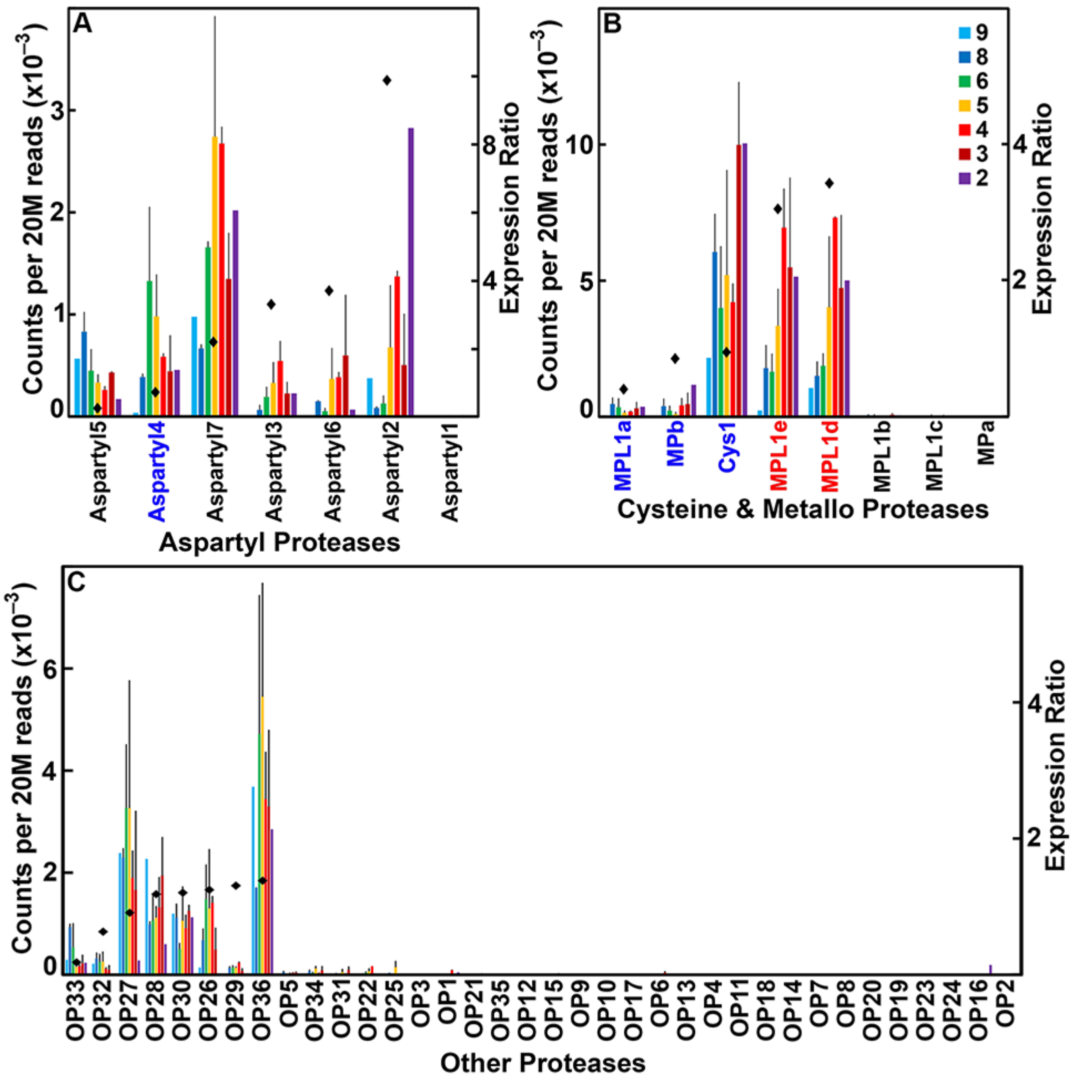

Figure S21. Differential expression of genes in families of maize genes of proteases involved in cell growth and cell wall metabolism. Expression ratios and potential Arabidopsis orthologs were determined as described in the legend of Figure S1. **A.** Aspartyl proteases. **B.** Cysteine- and metallo-proteases. **C.** Other proteases. [Maize Accession numbers for other proteases are in Additional file 2; Dataset 1]

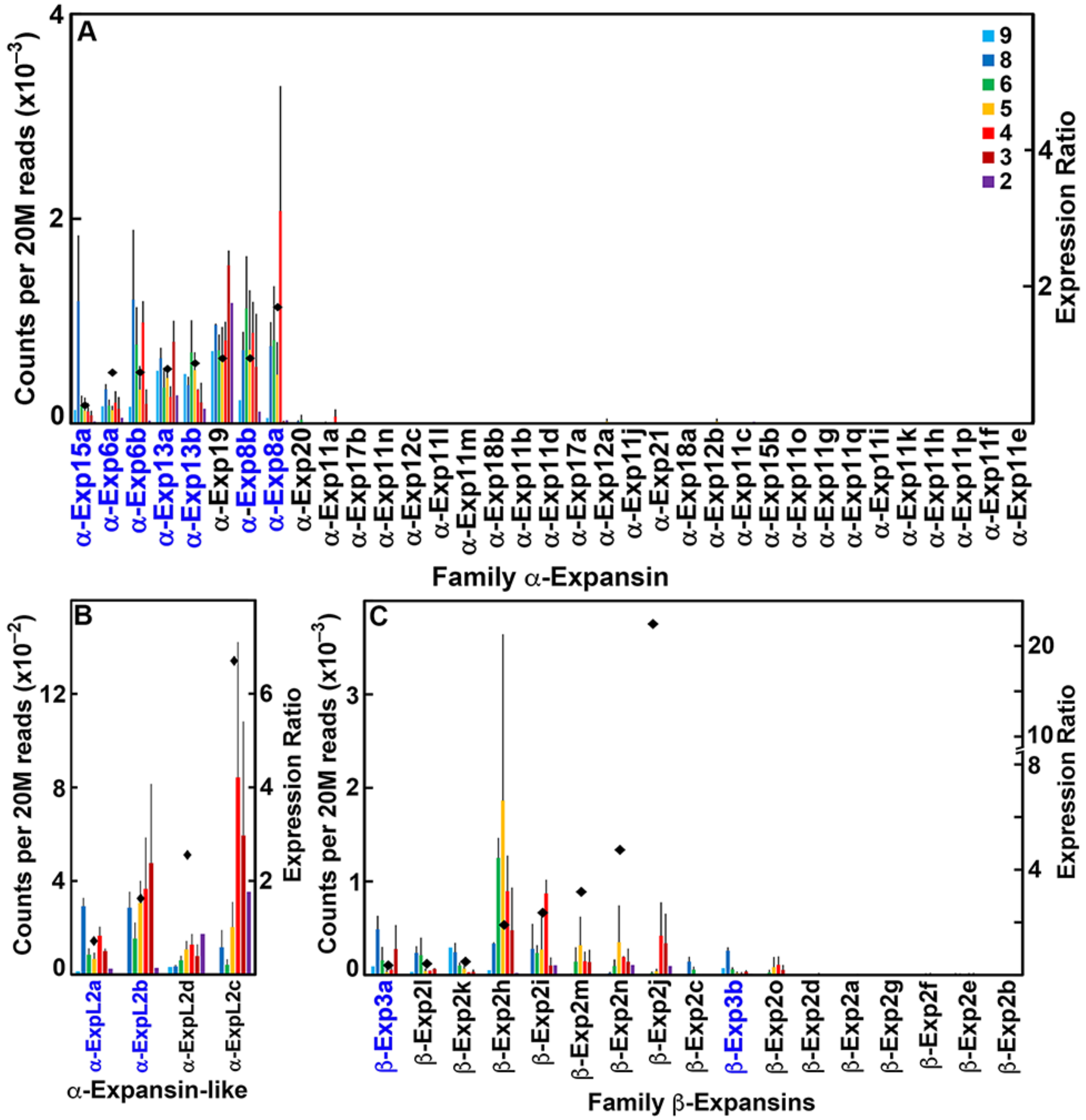

Figure S22. Differential expression of genes in the maize expansin superfamily. Expression ratios and potential Arabidopsis orthologs were determined as described in the legend of Figure S1. Family α-Expansins. **B.** Family α-Expansin-like. **C**. Family β-Expansins.

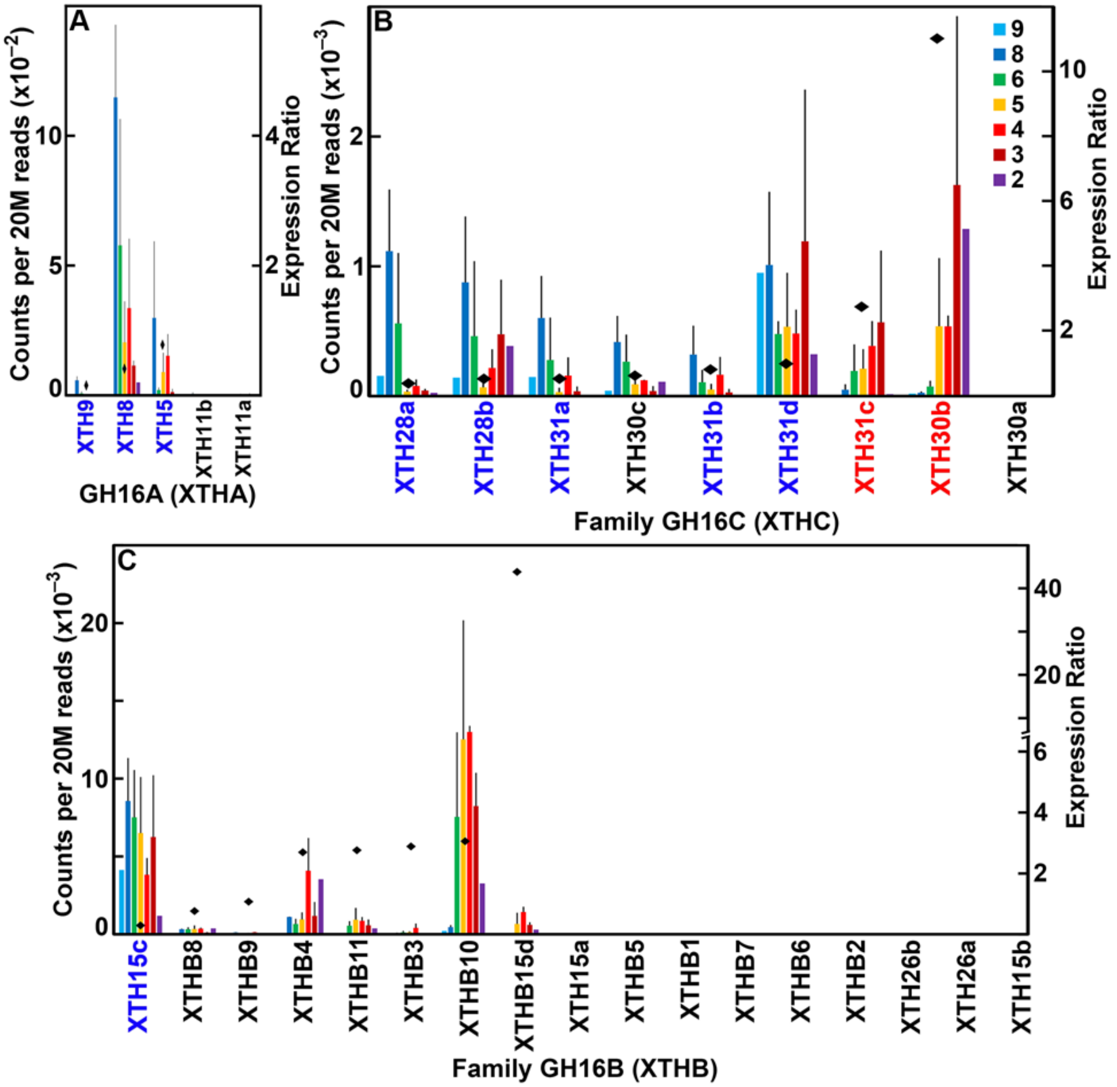

Figure S23. Differential expression of genes in the maize GH16 xyloglucan endo-β-D-glucan transferase/hydrolase superfamily. Expression ratios and potential Arabidopsis orthologs were determined as described in the legend of Figure S1. **A.** Family GH16 Group A (XTHA). **B.** GH16 Group C (XTHC). **C**. GH16 Group B (XTHB).
