# Supplementary material for: Expression profiles of cell-wall related genes vary broadly between two common maize inbreds during stem development
Source: BMC Genomics. 2019 Oct 29;20:785. doi: 10.1186/s12864-019-6117-z (PMC6819468; doi:10.1186/s12864-019-6117-z)
Supplement: Supplementary file 6 — Additional file 6: Figure S24. Principal Components Analysis of expression classes defined by hierarchical clustering. [file 12864_2019_6117_MOESM6_ESM.pdf]

Additional file 6: Figure S24

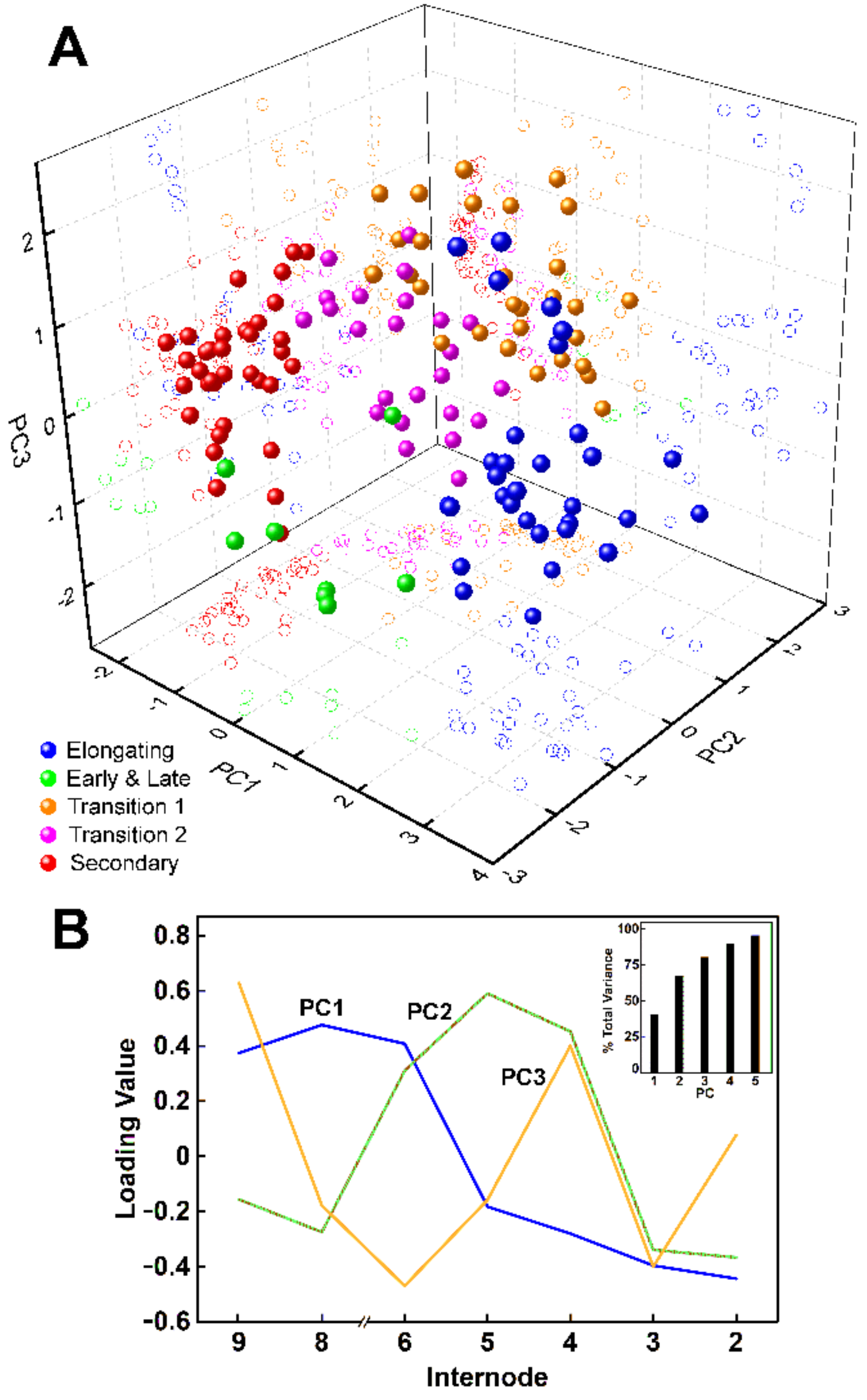

Figure S24. Principal Components Analysis of five expression classes defined by hierarchical clustering. A. Three-dimensional plot of the first three principal components (PCs) for genes classified by HC into five general patterns of expression. **B.** The loadings of the first three PCs, which capture over 80% of the variation. *Inset*: Cumulative % variance accounted for in the first five loadings.
