# Supplementary material for: Expression profiles of cell-wall related genes vary broadly between two common maize inbreds during stem development
Source: BMC Genomics. 2019 Oct 29;20:785. doi: 10.1186/s12864-019-6117-z (PMC6819468; doi:10.1186/s12864-019-6117-z)
Supplement: Supplementary file 7 — Additional file 7: Figure S25. Abundance within classes of genes that best fit the secondary wall pattern defined by slope-metric analysis. [file 12864_2019_6117_MOESM7_ESM.pdf]

Additional file 7: Figure S25

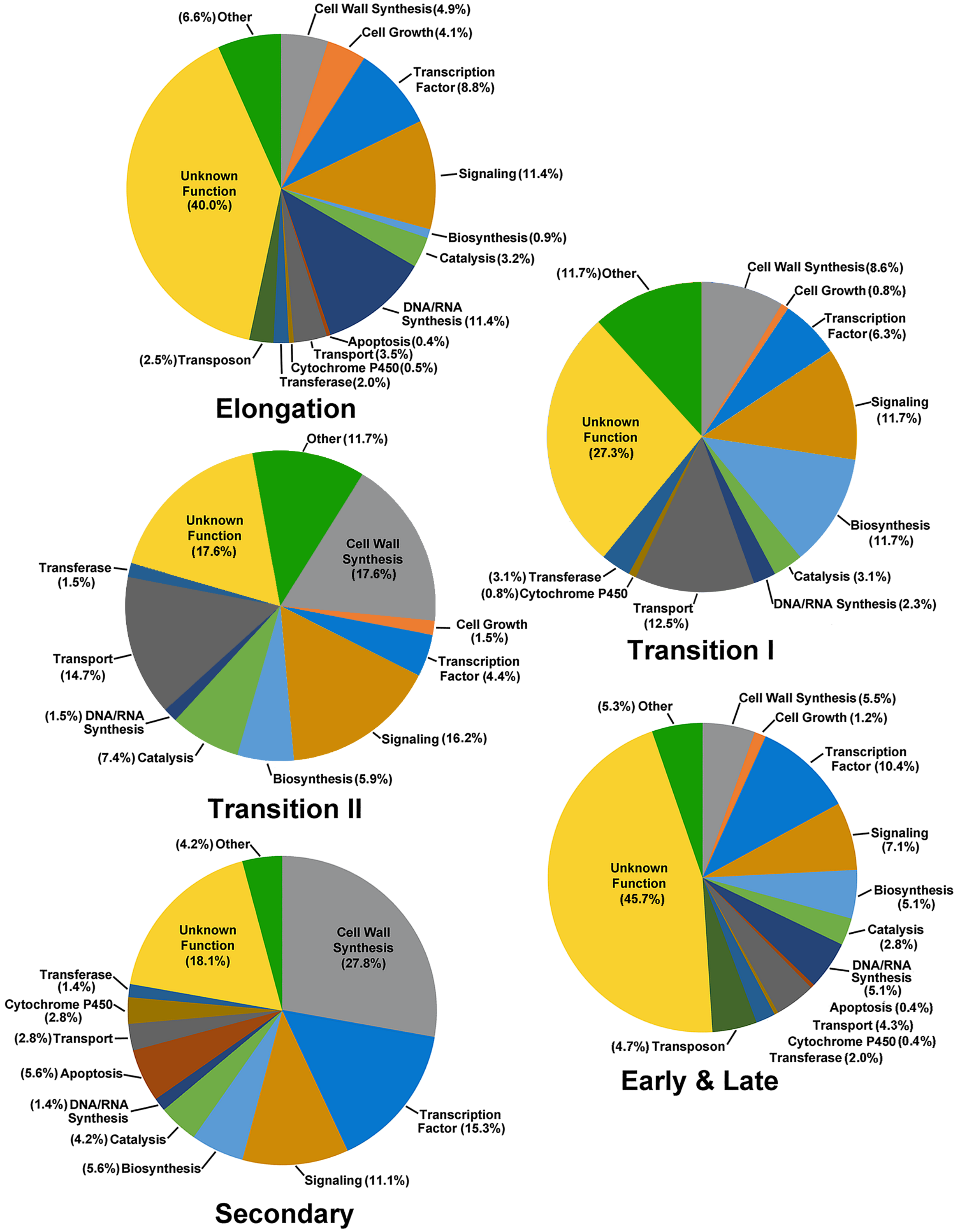

Figure S25. Abundance within classes of genes that best fit the developmental patterns of Elongation, Transition I, Transition II, Secondary wall, and Early and Late, as defined by slope-metric analysis (Figure 7).
