# Supplementary material for: Expression profiles of cell-wall related genes vary broadly between two common maize inbreds during stem development
Source: BMC Genomics. 2019 Oct 29;20:785. doi: 10.1186/s12864-019-6117-z (PMC6819468; doi:10.1186/s12864-019-6117-z)
Supplement: Supplementary file 8 — Additional file 8: Table S2. Numbers of each of the fifteen motifs identified by PromZea analysis in the promoter regions of genes expressed during the Secondary wall stage of development. Motifs and their classification by STAMP are provided in Fig. 8. [file 12864_2019_6117_MOESM8_ESM.pdf]

Additional file 8: Table S2. Numbers of each of the fifteen motifs identified by PromZea analysis in the promoter regions of genes expressed during the Secondary wall stage of development. Motifs and their classification by STAMP are provided in Figure 8.

Gene Name	Motif Number: Group 1				Group 2					Group 3		Group 4	Group 5		
	1	4	9	12	3	6	8	10	15	11	14	2	5	7	13
	Numbers of motifs														
GT47 IRX10-1c	2		2		1	1		1					1	1	1
CesA12b			3	1	1	1	1	1	1	1	1		1	1	1
CslE1b	1	2	7	1		1	1			1	1	1	1	1	1
FUTL11	2	2	8			1	1	1	2	1	1	1	1	1	1
PAL5	3	3	10	1	1	1	1	1		1	1	1	1	1	
HCTb			7	1		1	1	1	2	1	1	1	1	1	
GT43 IRX9Lc	1		3			1		1					1	1	1
GT47E IRX10-1a				1		1	1	1		1	1		1	1	1
GT47G IRX10-1e		1	4			1	1	1		1	1	3	1	1	1
PAL2	1	3	9	1	2	1	1			1	1	2	1	1	1
PRDA4	1		4	2		1	1	1			1	1	1	1	1
4CLL4			5			1	1		1	1	1		1	1	1
CCoAOMT1b		1	4		1	1	1	1	1	1	1	1	1	1	
F5Ha		2	5	2		1		1			1		1	1	1
GT43 IRX9Ld			5	3	1	1	1	2	1	1	1		1	1	
GT43 IRX9a	1	2	8	2	2	1	1	1	1	1	1		1	1	1
CCR1a	1	1	4	1	1			1	1		1		1	1	1
C3H1a		1	1		1	1		1	2		1		1	1	1
C3H1		1	4	2	1	1	1	1		1	1		1	1	1
CesA12a			5	1	1	1	1	1	1	1	1		1	1	
CesAL4			1					1					1	1	
HCTa	1		3			1	1						1	1	1
PAL1	2	1	8		1	1	1	1		1	1	2	1	1	1
PAL9	21	7	1	1	1	1	1	1		1	1	1	1	1	
Lac2a			1	1	1	1	1	1	1		1	1	1	1	1
Sus3					1	1		1		1	1		1	1	
4CLL6			6				1	1		1	1		1	1	1
CAD6		1	3			1	1				1		1	1	1
GT47E IRX10-1d		2	4	2		1	1	1	2	1	1		1	1	1
Total	18	24	131	23	17	26	22	25	17	18	25	14	28	29	24
