# Supplementary material for: Expression profiles of cell-wall related genes vary broadly between two common maize inbreds during stem development
Source: BMC Genomics. 2019 Oct 29;20:785. doi: 10.1186/s12864-019-6117-z (PMC6819468; doi:10.1186/s12864-019-6117-z)
Supplement: Supplementary file 9 — Additional file 9: Table S3. Twenty-five genes best fitting the Secondary wall pattern contain the PALBOXA promoter consensus sequence CCGTCC. [file 12864_2019_6117_MOESM9_ESM.pdf]

Additional file 9: Table S3. Twenty-five genes best fitting the Secondary wall pattern contain the PALBOXA promoter consensus sequence CCGTCC.[1]

Group	Gene	Start Location	Sequence
ATP binding protein	GRMZM2G180254	–873	CCGTCC*
[Unknown]	GRMZM2G056068	–300	CCGTCG
–	GRMZM2G056068	–337	CCGTCC*
–	GRMZM2G056068	–408	CCGTCC*
–	GRMZM2G056068	–752	CCGTCA
–	GRMZM2G056068	–988	CCGTCT
Fructose-1,6-bisphosphatase,	GRMZM2G322953	–526	CCGTCG
–	GRMZM2G322953	–911	CCGTCC*
ATP binding protein	GRMZM2G180244	–196	CCGTCA
–	GRMZM2G180244	–223	CCGTCG
–	GRMZM2G180244	–876	CCGTCA
YSL2, ATYSL2	GRMZM2G156599	–118	CCGTCG
–	GRMZM2G156599	–192	CCGTCG
[Unknown]	GRMZM2G477146	–67	CCGTCT
IRX3 (CESA7)	GRMZM2G011651	–407	CCGTCG
–	GRMZM2G011651	–689	CCGTCC*
–	GRMZM2G011651	–887	CCGTCC*
–	GRMZM2G011651	–896	CCGTCG
CesA12b	GRMZM2G002523	–415	CCGTCG
–	GRMZM2G002523	–697	CCGTCC*
–	GRMZM2G002523	–895	CCGTCC*
–	GRMZM2G002523	–904	CCGTCG
Ankyrin repeat family protein	GRMZM2G016668	–193	CCGTCT
[Unknown]	GRMZM2G142379	–40	CCGTCG
CYP72A15	GRMZM2G129860	–169	CCGTCG
–	GRMZM2G129860	–957	CCGTCC*
ANNAT3, Arabidopsis Annexin3	GRMZM2G132442	–415	CCGTCC*
–	GRMZM2G132442	–437	CCGTCC*
–	GRMZM2G132442	–486	CCGTCC*
–	GRMZM2G132442	–508	CCGTCC*
–	GRMZM2G132442	–559	CCGTCC*
FLA13, Fasciclin13	GRMZM2G001514	–213	CCGTCG
NAM, ANAC018, ATNAM, NARS2	GRMZM2G100583	–858	CCGTCA
Aldo/keto Reductase family protein	GRMZM2G024315	–29	CCGTCG
–	GRMZM2G024315	–155	CCGTCG
–	GRMZM2G024315	–339	CCGTCA
–	GRMZM2G024315	–558	CCGTCT
–	GRMZM2G024315	–751	CCGTCT
–	GRMZM2G024315	–779	CCGTCT
CesA12a	GRMZM2G142898	–905	CCGTCC*
[Unknown]	GRMZM5G892467	–971	CCGTCG
Alpha/beta fold hydrolase	GRMZM2G467007	–673	CCGTCT

[1] PAL-box motif from: Kaothien P, Shimokawatoko Y, Kawaoka A, Yoshida K, Shinmyo A. Plant Cell Rep. 2000;19:558-62.
* Exact match to PALBOXA promoter sequence
