# Supplementary material for: Expression profiles of cell-wall related genes vary broadly between two common maize inbreds during stem development
Source: BMC Genomics. 2019 Oct 29;20:785. doi: 10.1186/s12864-019-6117-z (PMC6819468; doi:10.1186/s12864-019-6117-z)
Supplement: Supplementary file 10 — Additional file 10: Figure S26. Comparison of cellulose, lignin, and sugar accumulation in developing internodes of greenhouse-grown B73 and Mo17. [file 12864_2019_6117_MOESM10_ESM.pdf]

Additional file 10: Figure S26

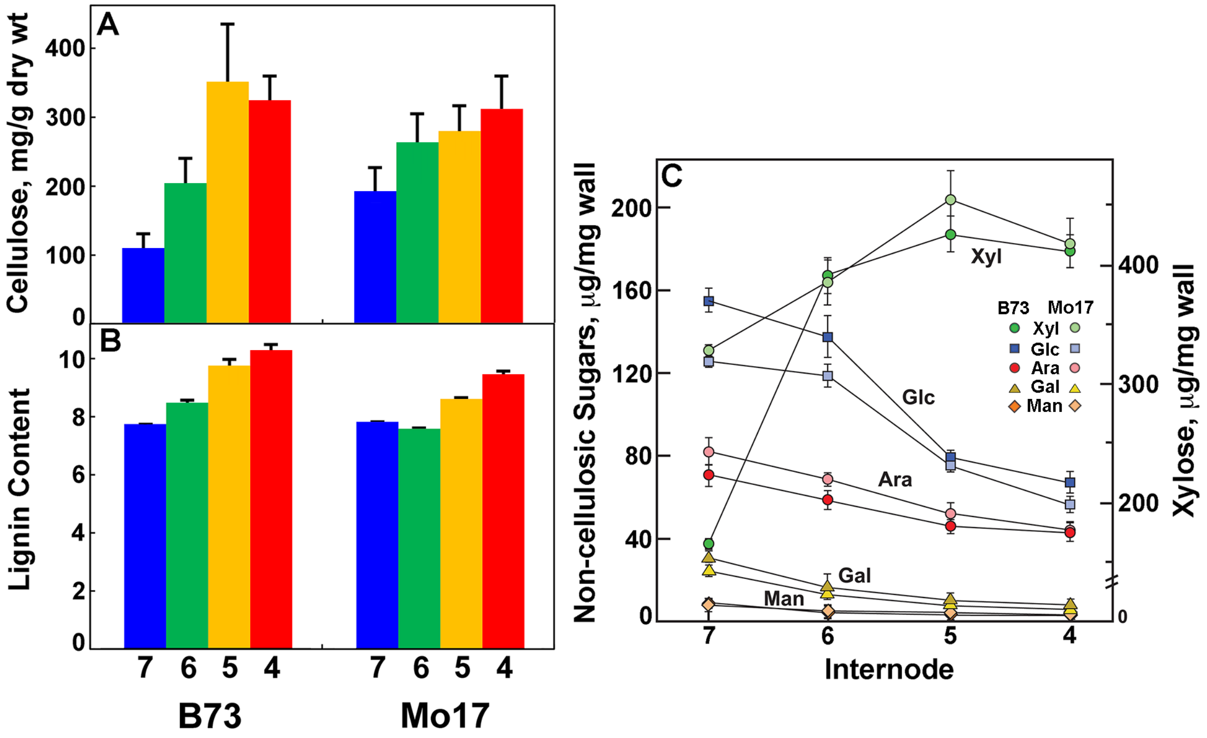

Figure S26. Comparison of cellulose and relative lignin content in developing internodes of inbreds B73 and Mo17. A. Crystalline cellulose was determined by acetic-nitric digestion. **B**. Relative lignin content was determined by Pyrolysis molecular-beam MS. **C**. Non-cellulosic monosaccharide distribution was determined by GC-MS separation of alditol acetates. Values are the mean ± S.D. of three independent samples.
