# Supplementary material for: Expression profiles of cell-wall related genes vary broadly between two common maize inbreds during stem development
Source: BMC Genomics. 2019 Oct 29;20:785. doi: 10.1186/s12864-019-6117-z (PMC6819468; doi:10.1186/s12864-019-6117-z)
Supplement: Supplementary file 12 — Additional file 12: Figures S27-S52. Comparative expression of maize B73 and Mo 17 gene families during stem development. [file 12864_2019_6117_MOESM12_ESM.pdf]

**Additional file 12. Figures S27-S53**. Comparative expression of maize B73 and Mo 17 gene families during stem development.

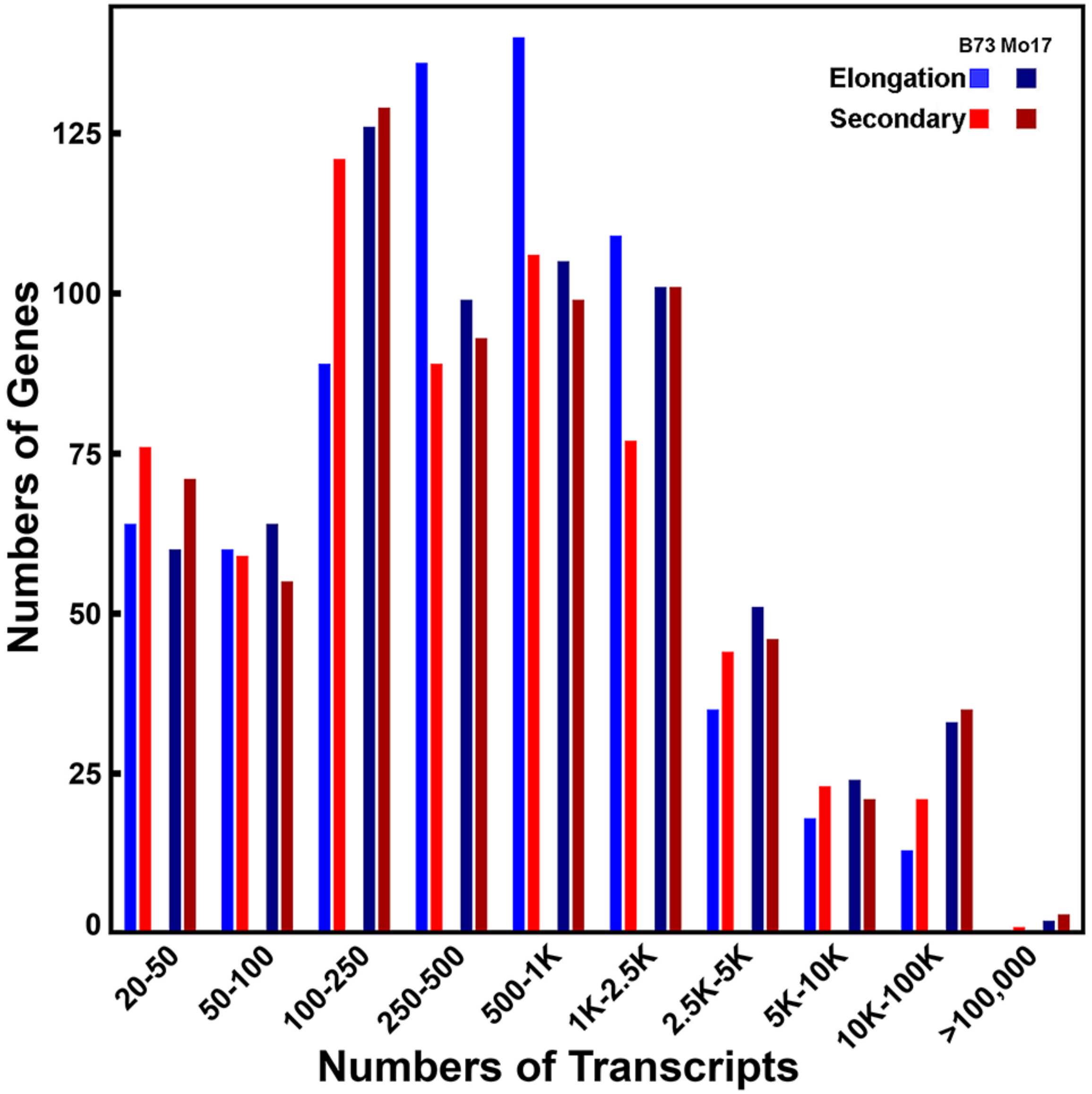


**Figure S27. Size distributions of transcript abundances of maize B73 and Mo17 expressed during stem development.** Transcript levels in rind tissues from Elongation stages (Internodes 7 and 6) and Secondary wall synthesis stages (Internodes 5 and 4) of greenhouse-grown plants were normalized and compared as counts per 20M reads.

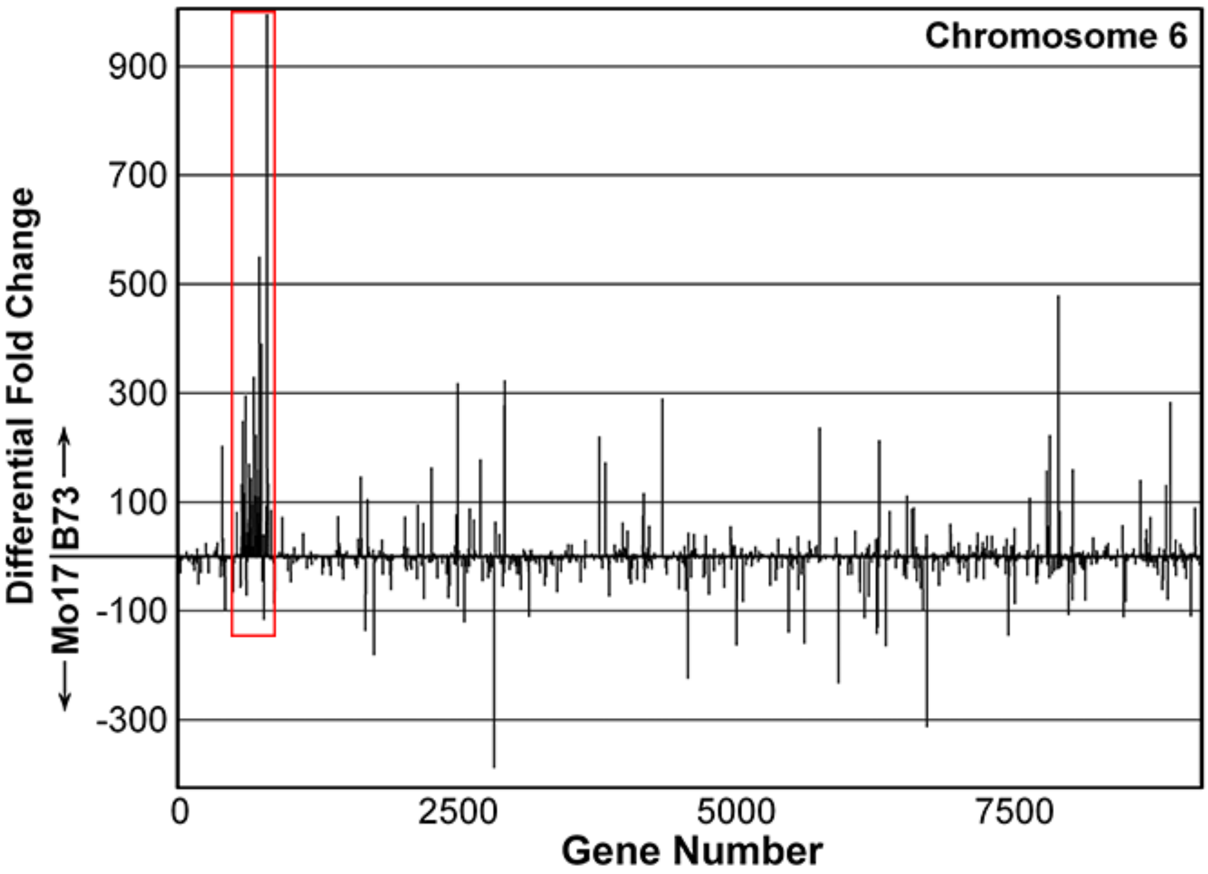


**Figure S28**. Example of variation in fold-change of expression for B73 and Mo73 during secondary wall formation across maize Chromosome 6. In Chromosome 6 of Mo17 deletions across 2.7 Mb yield 53 genes in B73 that show high relative expression because of their absence in Mo17 (red box).

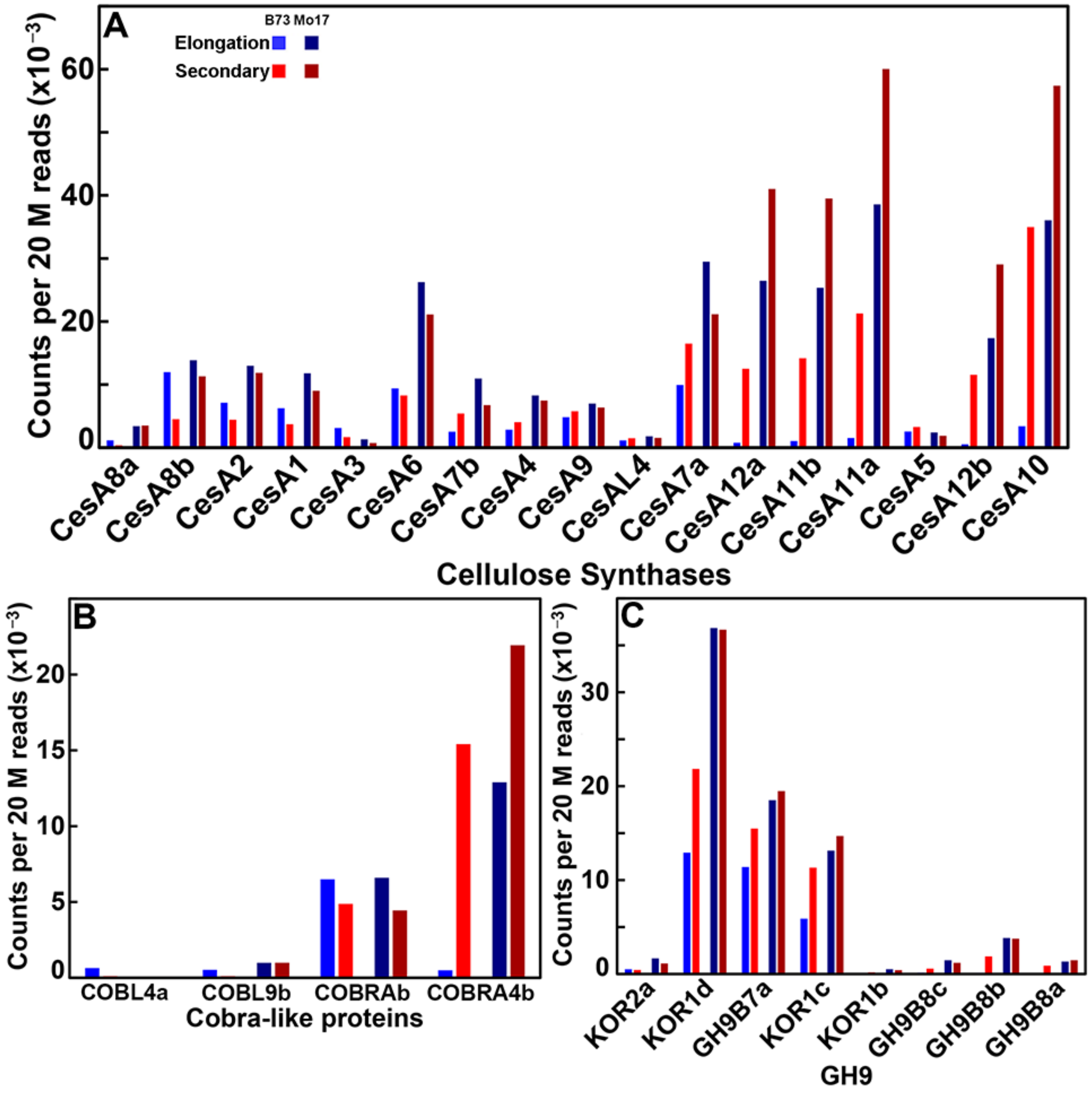


**Figure S29. Comparative expression of the maize B73 and Mo17 cellulose synthase (CesA), COBRA and KORRIGAN (KOR) gene family members during stem development.** Transcript levels were determined as described in the legend of Figure S27. **A.** The Cellulose Synthase (CesA) gene family. **B.** The COBRA-like gene family of GPI-anchored proteins. **C.** The Glycosyl Hydrolase 9 gene family, containing members of the KORRIGAN family.

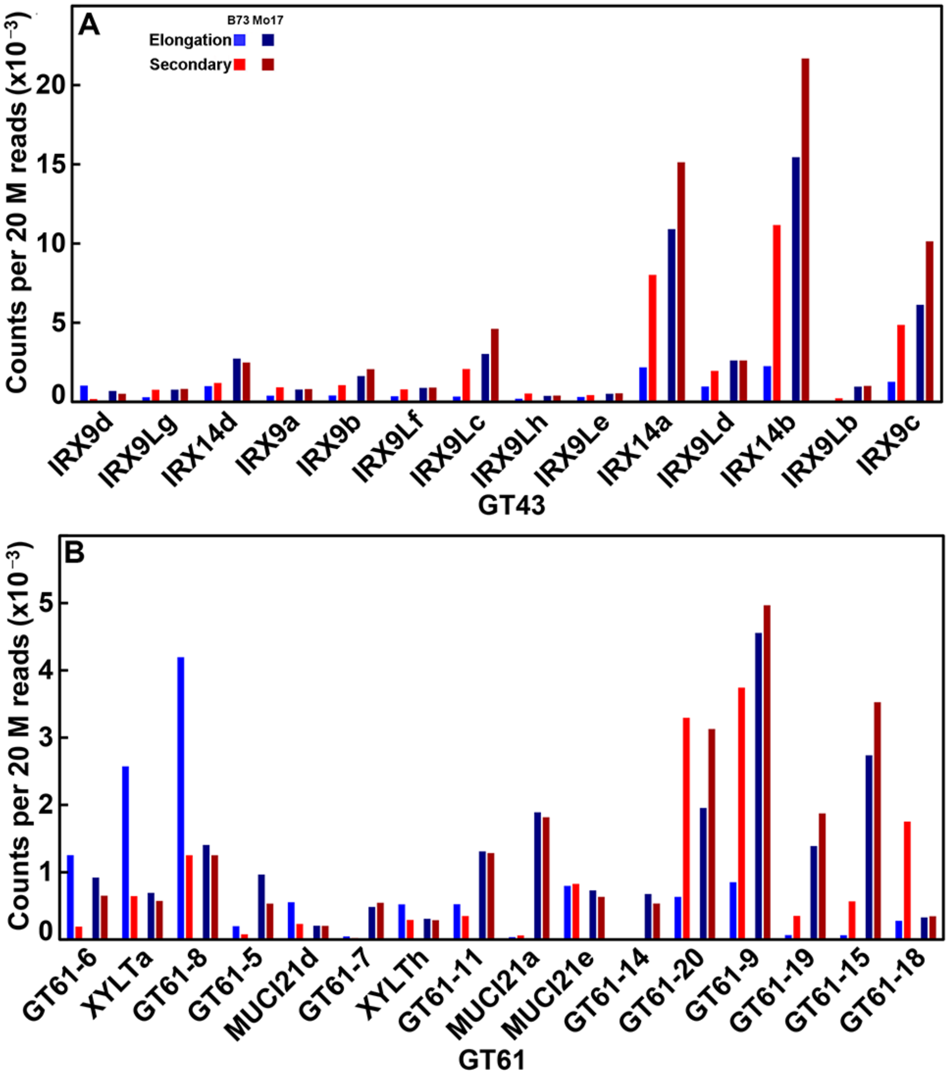


**Figure S30. Comparative expression of the maize B73 and Mo17 genes associated with glucuronoarabinoxylan synthesis during stem development.** Transcript levels were determined as described in the legend of Figure S27. **A**. Family GT43, containing xylan xylosyl transferases. **B**. Family GT61, containing xylan arabinosyl- and xylosyl transferases.

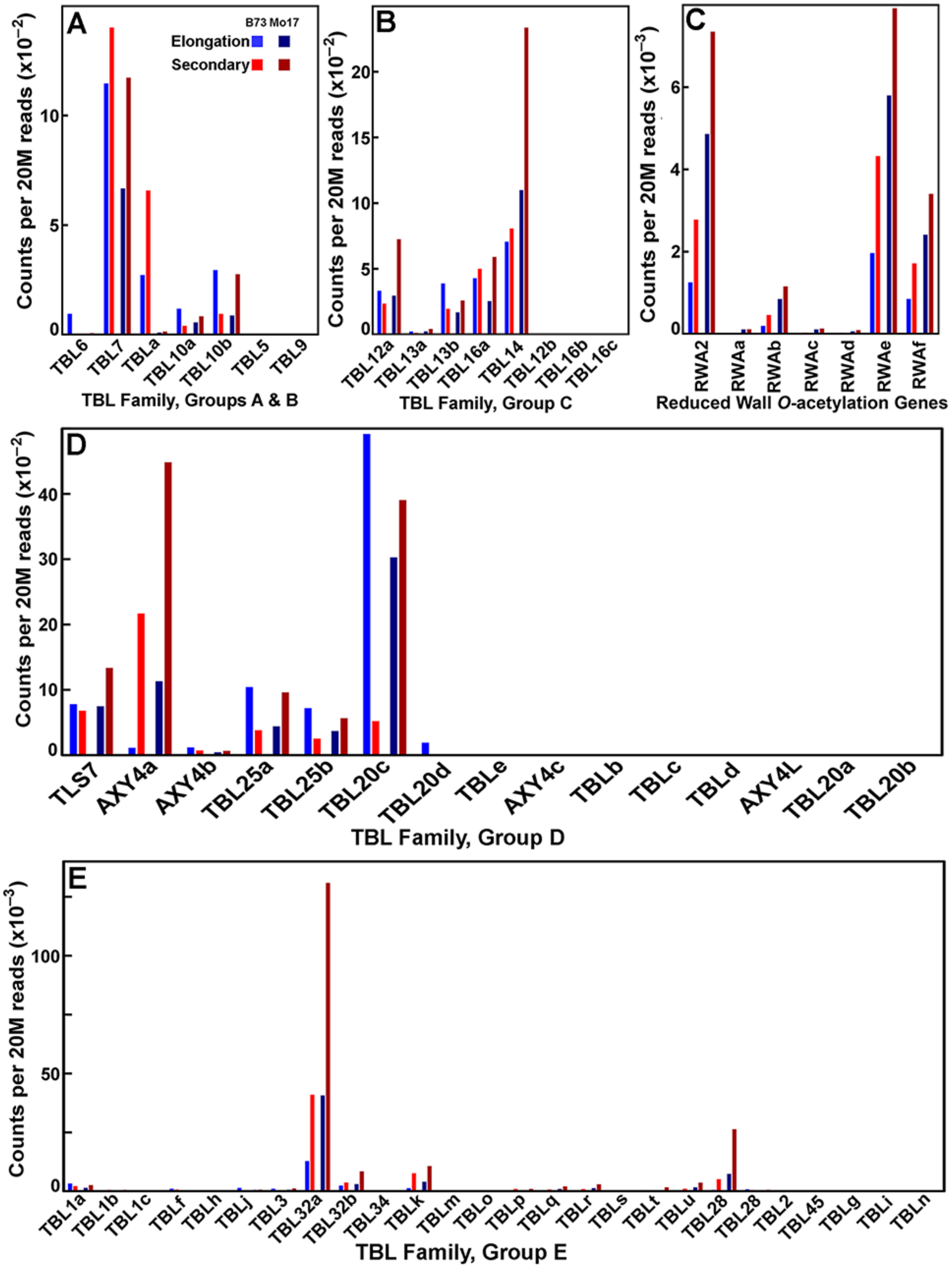


**Figure S31. Comparative expression of the maize B73 and Mo17 genes associated with acetylation of xylans.** Transcript levels were determined as described in the legend of Figure S27. **A**. Family TBL, Groups A and B. **B.** Family TBL, Group C. **C.** Family Reduced Wall *O*-Acetylation (RWA). **D.** Family TBL, Group D. **E.** Family TBL, Group E.

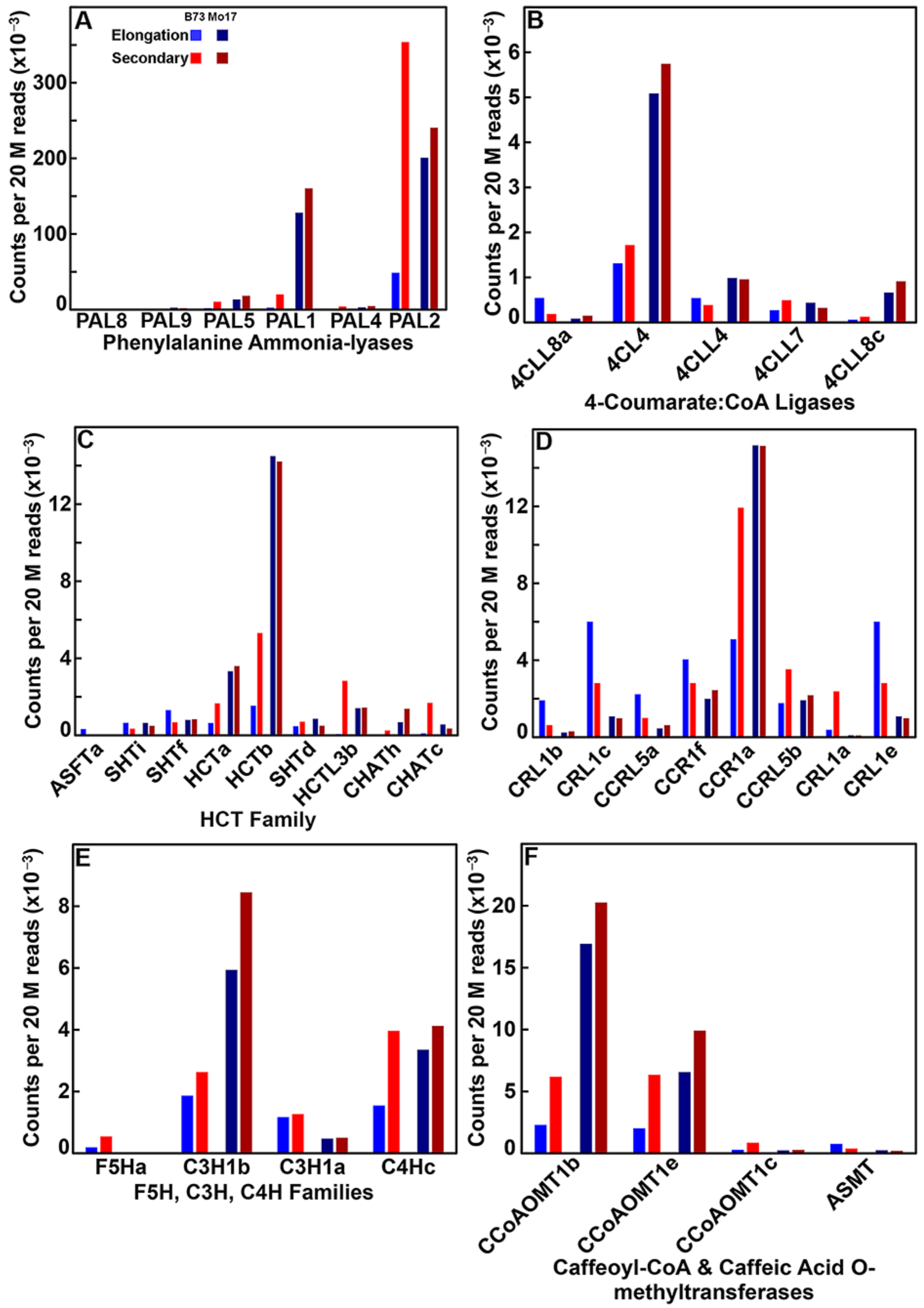


**Figure S32. Comparative expression of maize B73 and Mo17 genes associated with monolignol synthesis during stem development.** Transcript levels were determined as described in the legend of Figure S27. **A.** Family PAL, phenylalanine ammonia lyases. **B**. Family 4CL, 4-coumarate CoA ligases. **C**. Family HCT, Hydroxycinnamoyl-CoA:Shikimate Hydroxycinnamoyl Transferases. **D.** Family CCR, cinnamoyl-CoA reductases. **E.** Families C3H (coumarate-3-hydroxylases), C4H (cinnamate-4-hydroxylases), and F5H (ferulate-5-hydroxylases). **F.** Family CCoAOMT, caffeoyl-CoA *O*-methyltransferases, and COMT, caffeic acid *O*-methyltransferases.

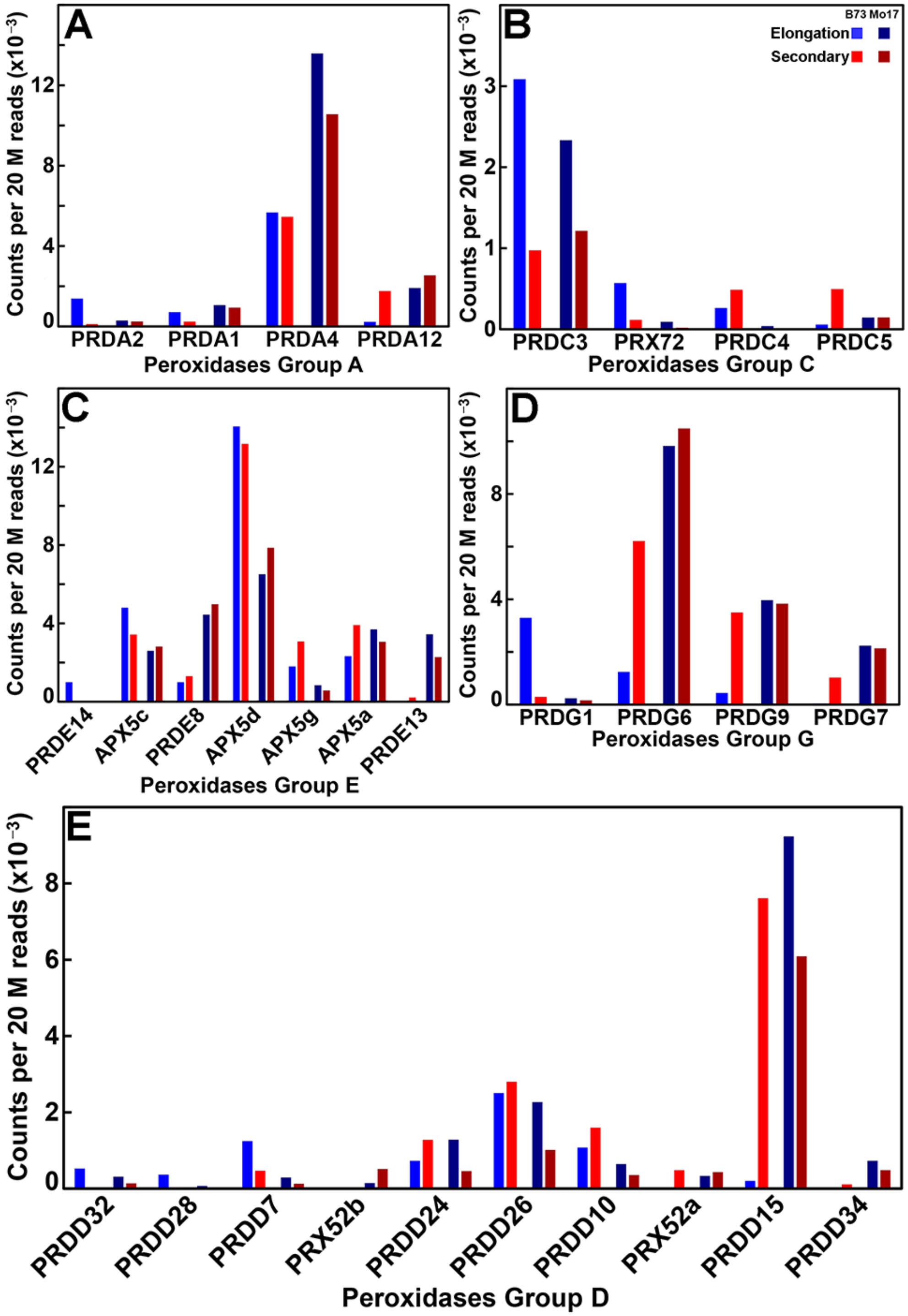


**Figure S33. Comparative expression of maize B73 and Mo17 genes of the peroxidase superfamily.** Transcript levels were determined as described in the legend of Figure S27. **A**. Family Peroxidase, subgroup A. **B**. Family Peroxidase, subgroups B and C. **C**. Family Peroxidase, subgroups E and F. **D.** Family Peroxidase, subgroup G. **E.** Family Peroxidase, subgroup D.

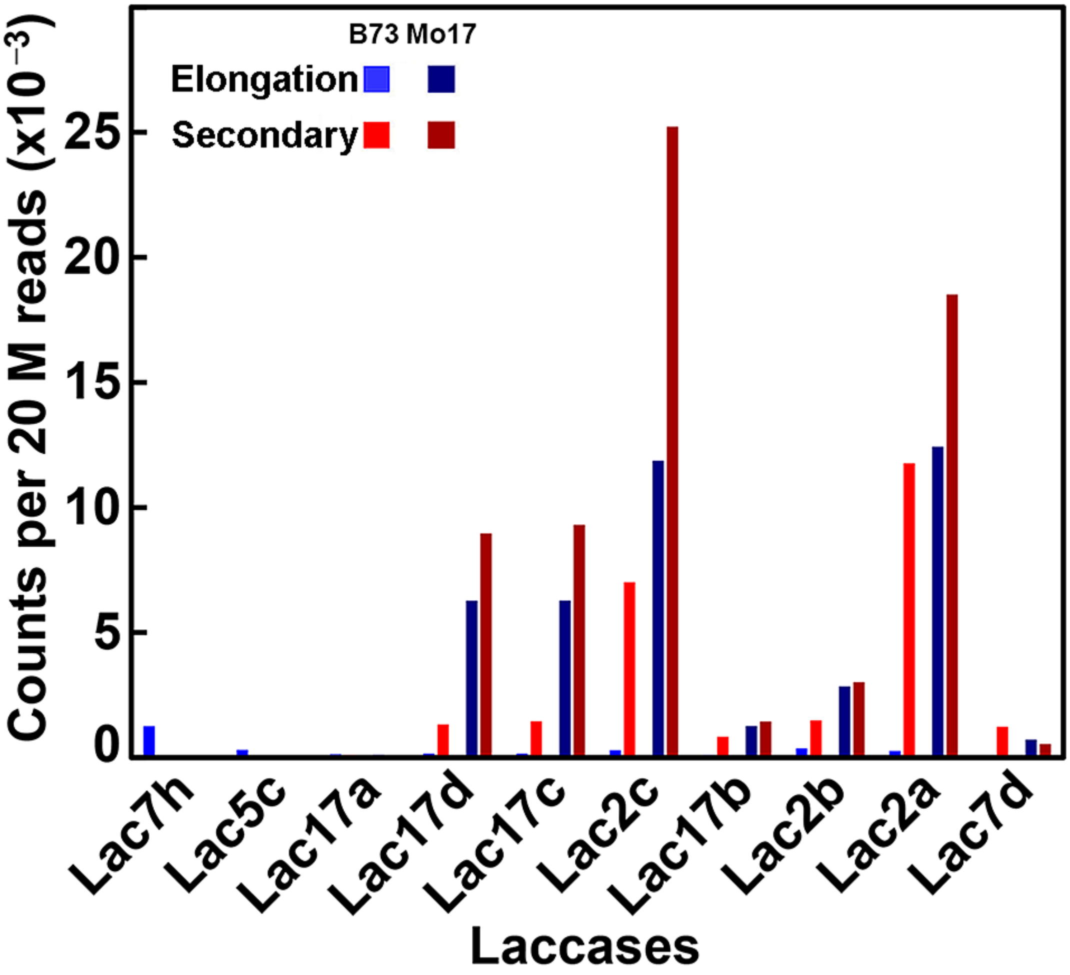


**Figure S34. Comparative expression of maize B73 and Mo17 genes of the laccase family.** Transcript levels were determined as described in the legend of Figure S27.

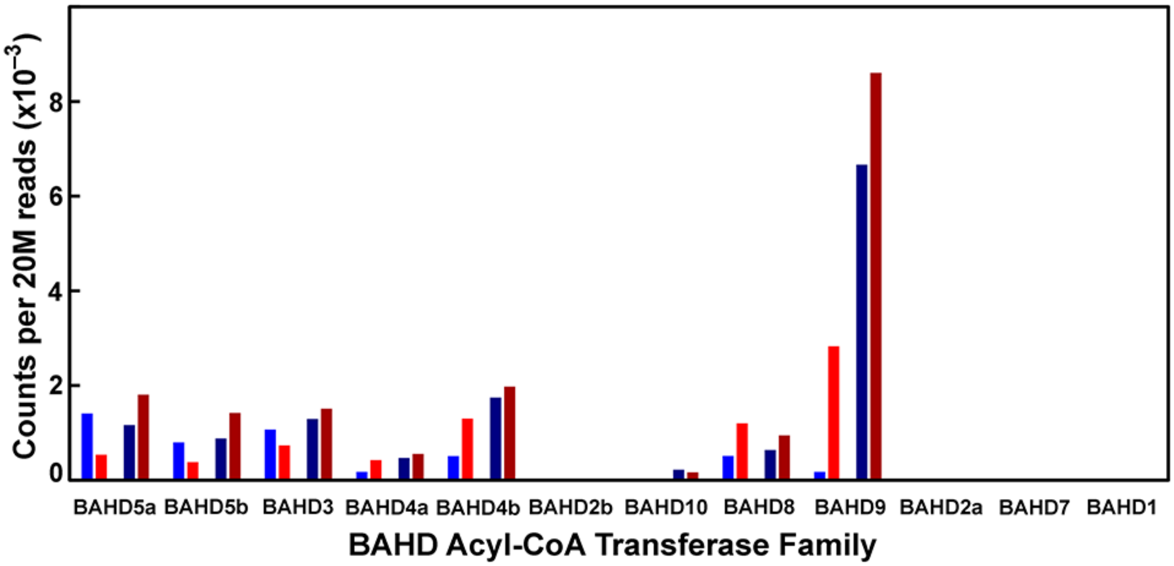


**Figure S35. Comparative expression of maize B73 and Mo17 genes of the BAHD family of CoA-dependent acyl-transferases.** Transcript levels were determined as described in the legend of Figure S27.

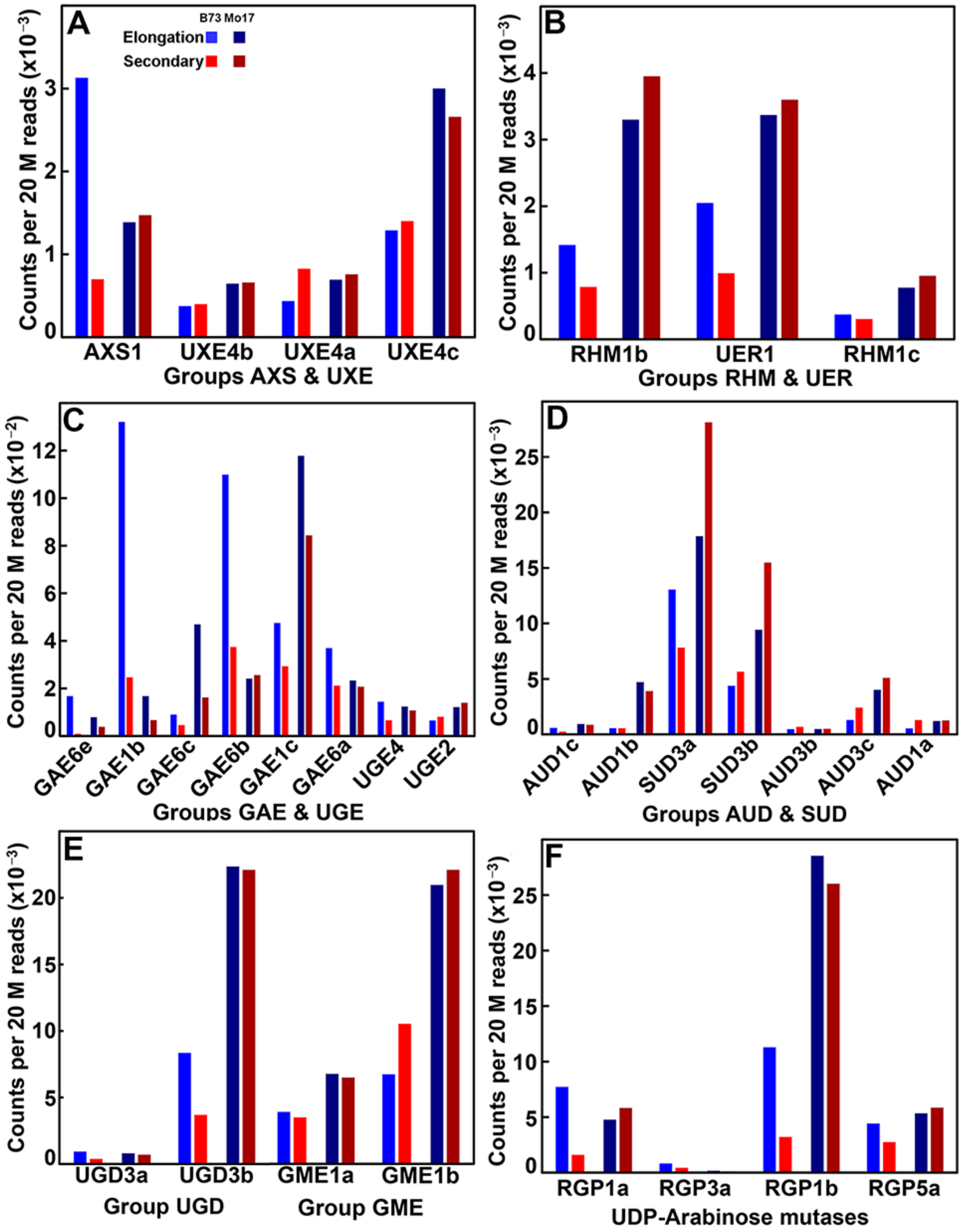


**Figure S36. Comparative expression of maize B73 and Mo17 genes in families associated with nucleotide-sugar interconversion.** Transcript levels were determined as described in the legend of Figure S27. **A.** Family AXS, UDP-D-apiose/UDP-D-xylose synthases; and Family UXE, UDP-D-xylose 4-epimerases. **B**. Family RHM, UDP-L-rhamnose synthases, and Family UER, UDP-4-keto-6-deoxy-D-glucose 3,5-epimerase-4-reductases **C.** Family GAE, UDP-D-glucuronate 4-epimerases; and Family UGE, UDP-D-glucose 4-epimerases. **D**. Family AUD, membrane-anchored UDP-D-glucuronate decarboxylases; and SUD, soluble UDP-D-glucuronate decarboxylases. **E**. Family UGD, UDP-D-glucose dehydrogenases; and Family GME, GDP-D-mannose 3,5-epimerase; and **F**. Family GT75, RGP (UAM), Reversibly Glycosylated Proteins (UDP-L-arabinose mutases).

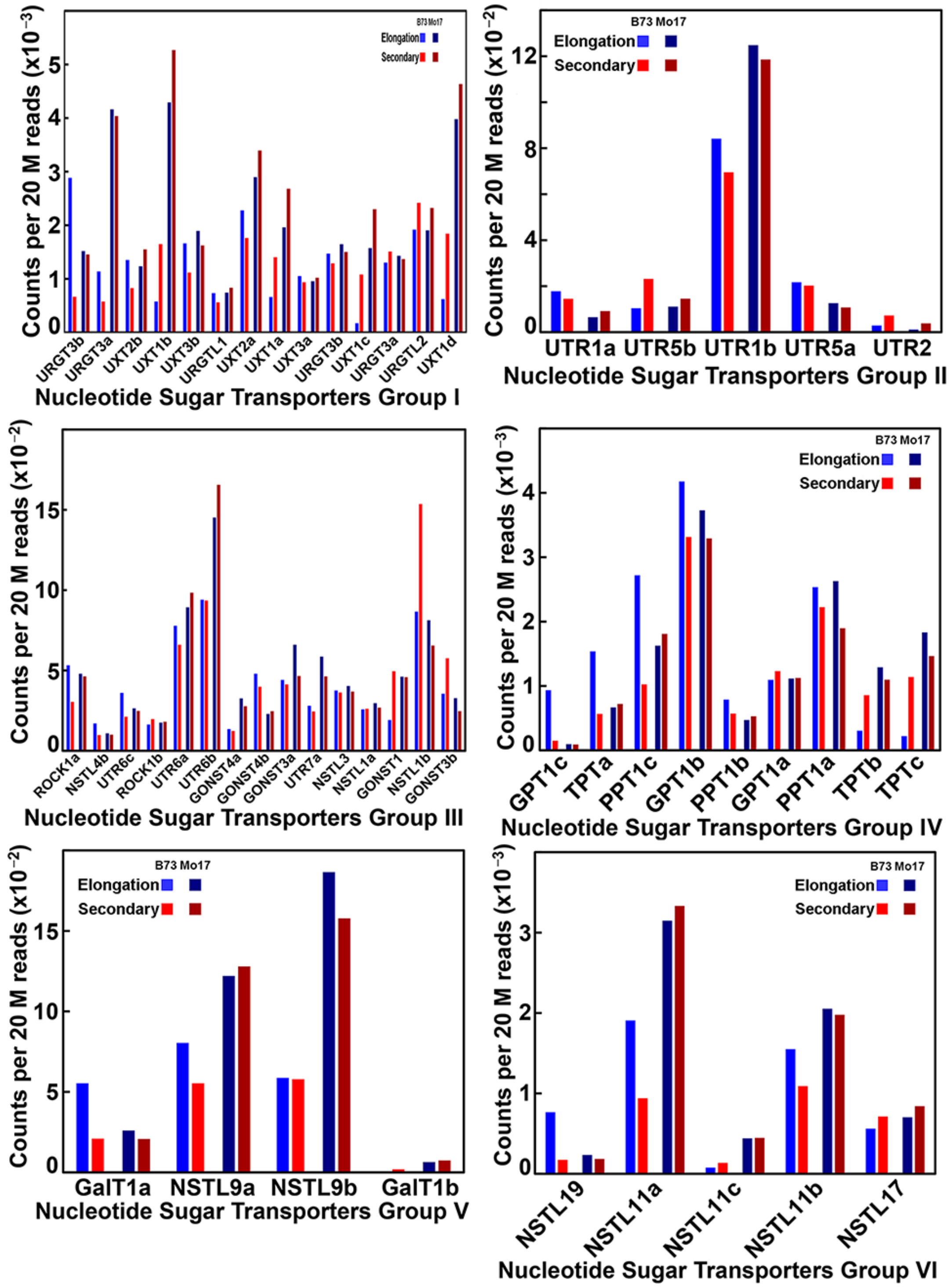


**Figure S37. Comparative expression of maize B73 and Mo17 genes associated with nucleotide-sugar transport.** Transcript levels were determined as described in the legend of Figure S27. **A.** Group I transporters, **B.** Group II transporters, **C.** Group III transporters, **D.** Group IV transporters, **E** Group V transporters, **F.** Group VI transporters.

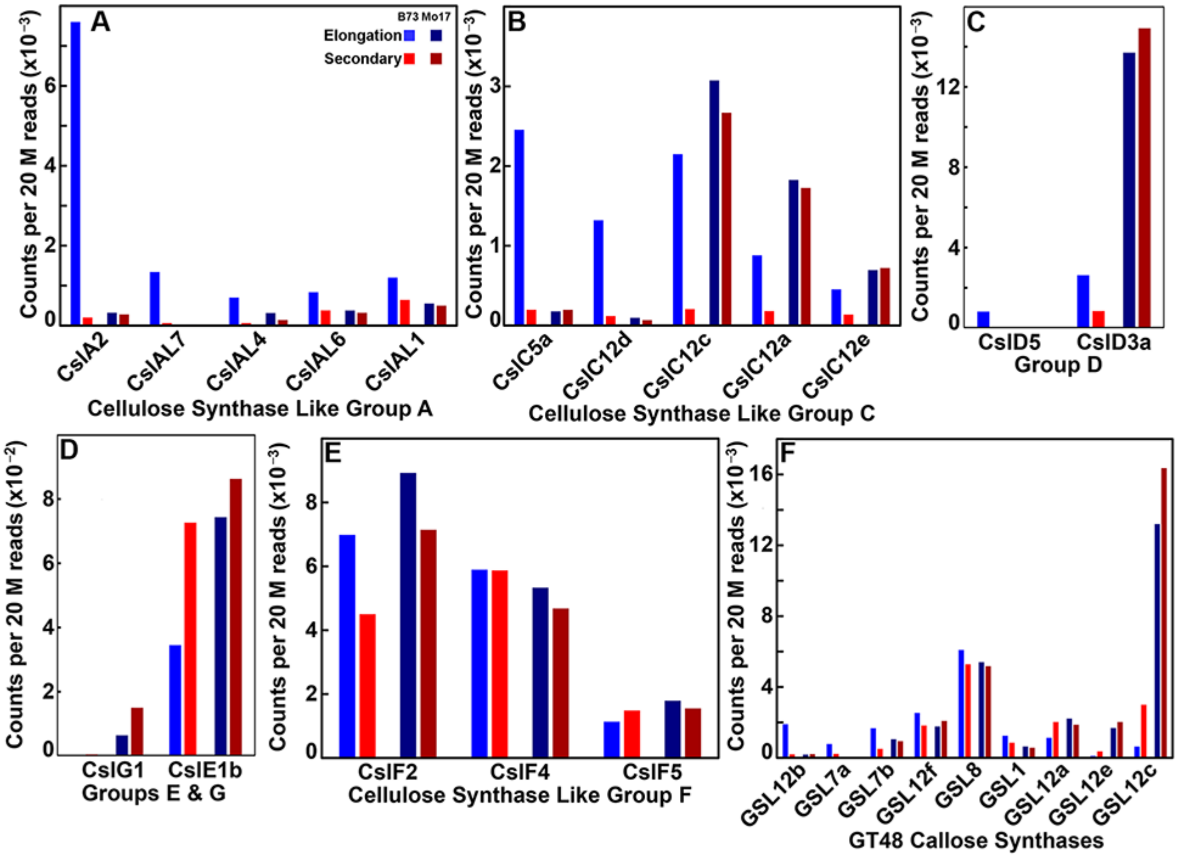


**Figure S38. Comparative expression of maize B73 and Mo17 genes associated with non-cellulosic glycan synthesis.** Transcript levels were determined as described in the legend of Figure S27. **A.** Family CslA, (1→4)-β-D-mannan and glucomannan synthases. **B.** Family CslC, putative xyloglucan (1→4)-β-D-glucan backbone synthases. **C.** Family CslD, (1→4)-β-D-glucan synthases **D.** Family CslE & CslG, putative (1→4)-β-D-glycan synthases. **E.** Family CslF, mixed-linkage (1→3),(1→4)-β-D-glucan synthases. **F.** Callose synthases**.**

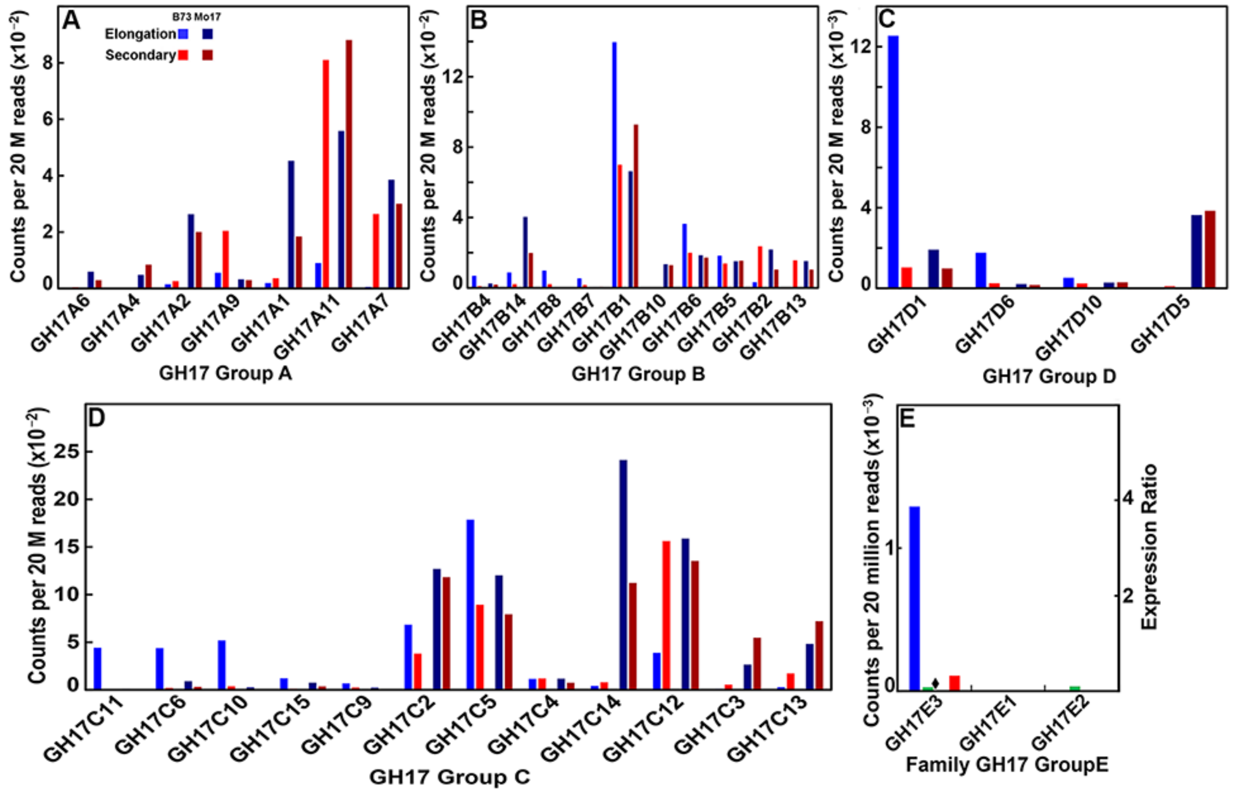


**Figure S39. Comparative expression of maize B73 and Mo17 genes associated with (1→3)-β-D-glucan (callose) hydrolysis.** Transcript levels were determined as described in the legend of Figure S27. **A.** Family GH17 Group A. **B.** Family GH17 Group B. **C.** Family GH17 Group D. **D.** Family GH17 Group C. **E.** Family GH17 Group E.

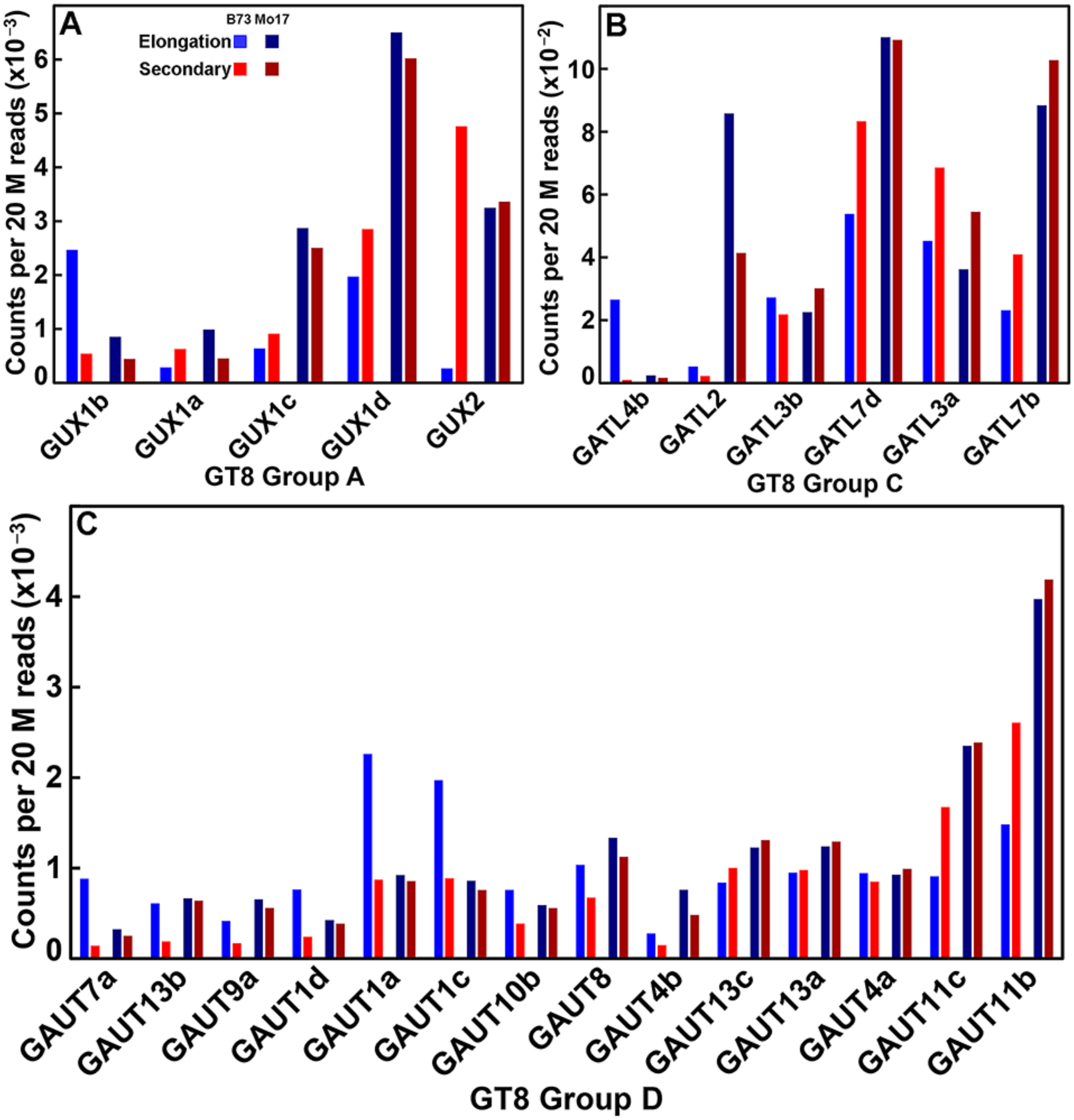


**Figure S40. Comparative expression of maize B73 and Mo17 genes of subgroups of Family GT8 retaining glycosyl transferases.** Transcript levels were determined as described in the legend of Figure S27. **A.** Family GT8 Group A, GUX, UDP-GlcA transferases. **B.** Family GT8 Group C, GATL, pectin UDP-D-GalA transferase-like. **C.** Family GT8 Group D, GAUT, pectin UDP-D-GalA transferases.

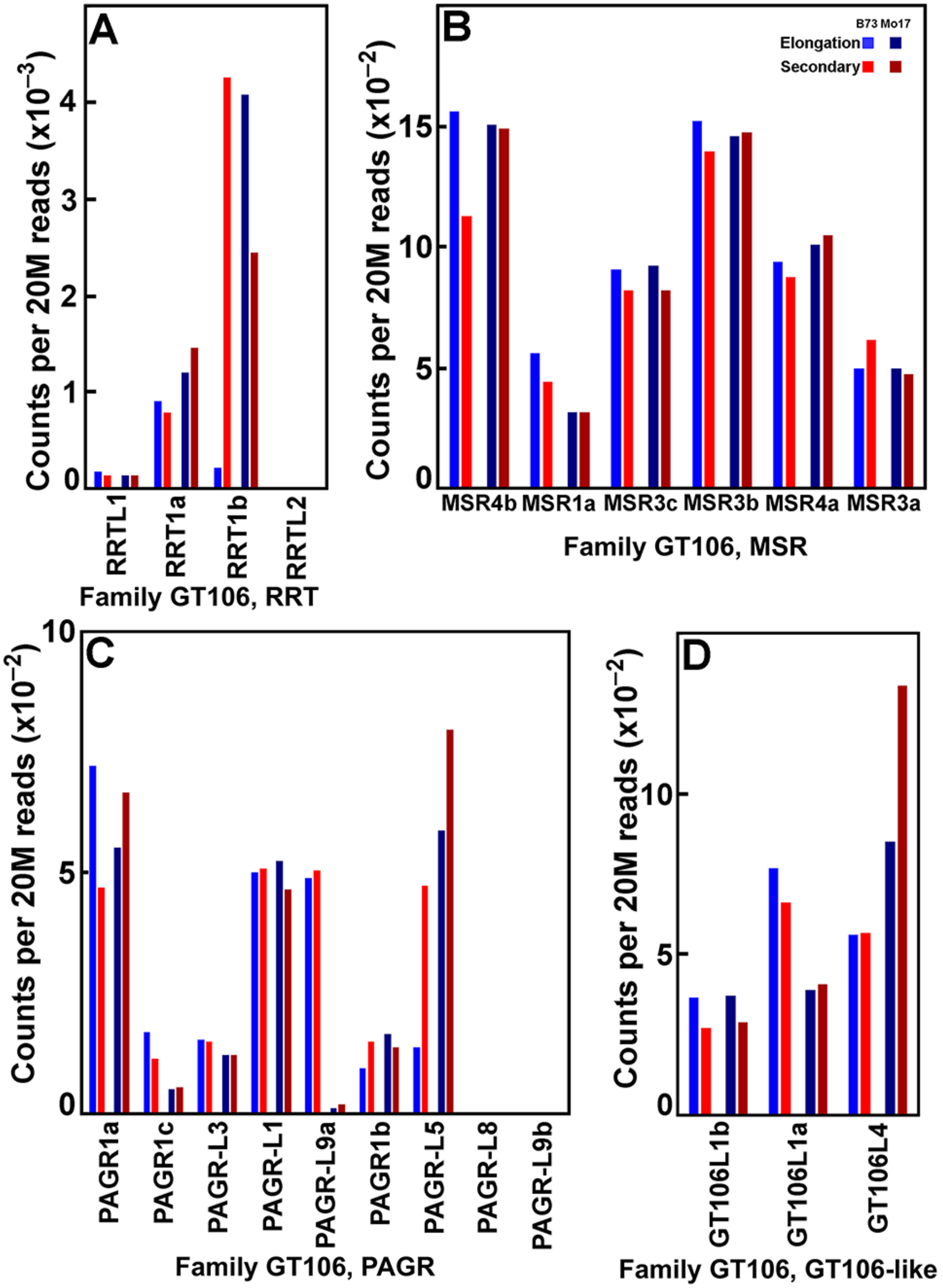


**Figure S41. Comparative expression of maize B73 and Mo17 genes of subgroups of Family GT106 inverting glycosyl transferases.** Transcript levels were determined as described in the legend of Figure S27. **A.** Family GT106, subgroup A of pectin-related rhamnosyl transferases (RRT). **B.** Family GT106, subgroup B of mannosyl transferases (MSR). **C.** Family GT106, subgroup C of pectin type I arabinogalactan-related transferases (PAGR). **D.** Family GT106-like, subgroup D.

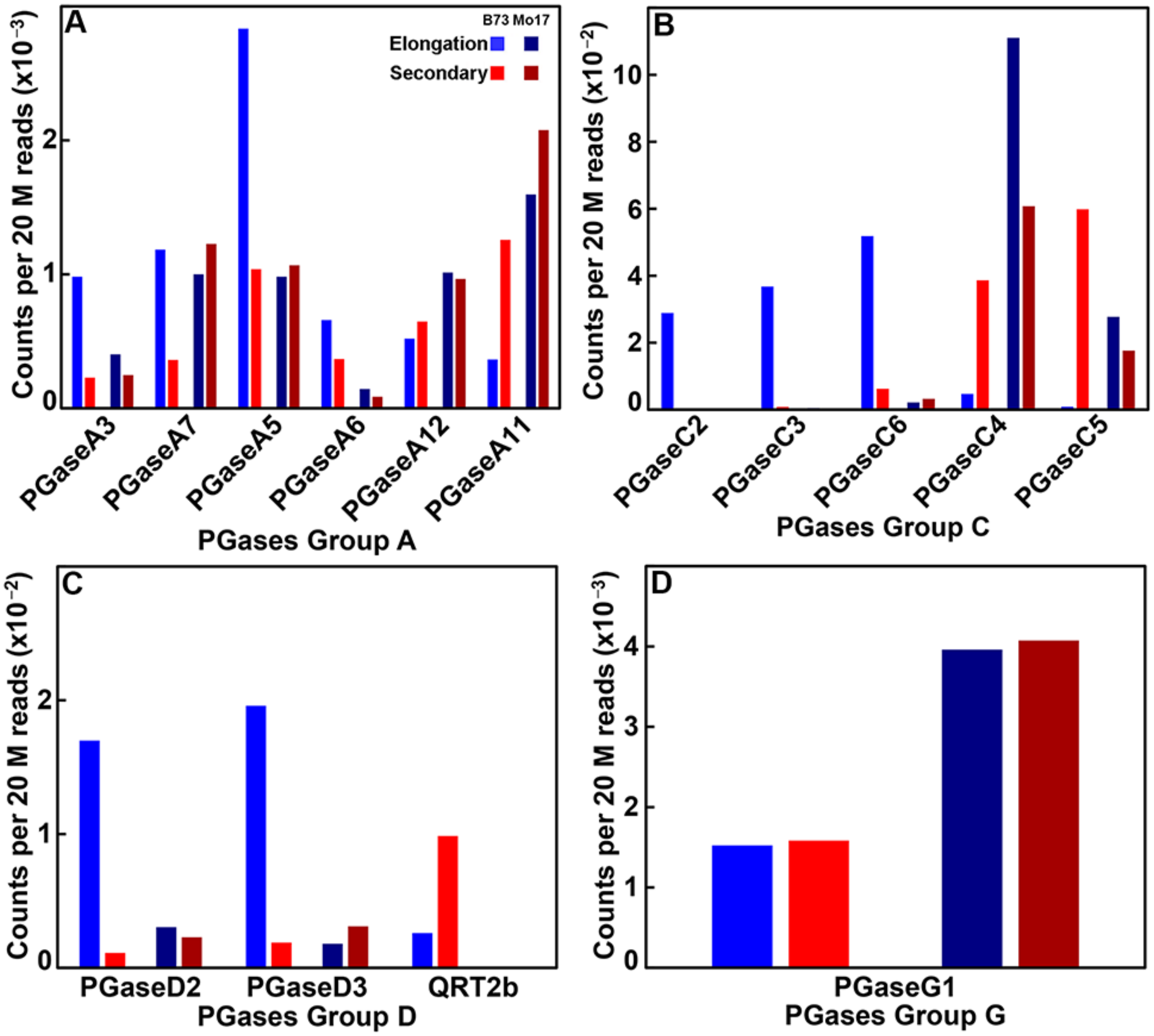


**Figure S42. Comparative expression of maize B73 and Mo17 genes of the GH28 polygalacturonase superfamily.** Transcript levels were determined as described in the legend of Figure S27. **A**. Family polygalacturonase, subgroup A. **B**. Family polygalacturonase, subgroup C. **C**. Family polygalacturonase, subgroup D. **D**. Family polygalacturonase, subgroup G.

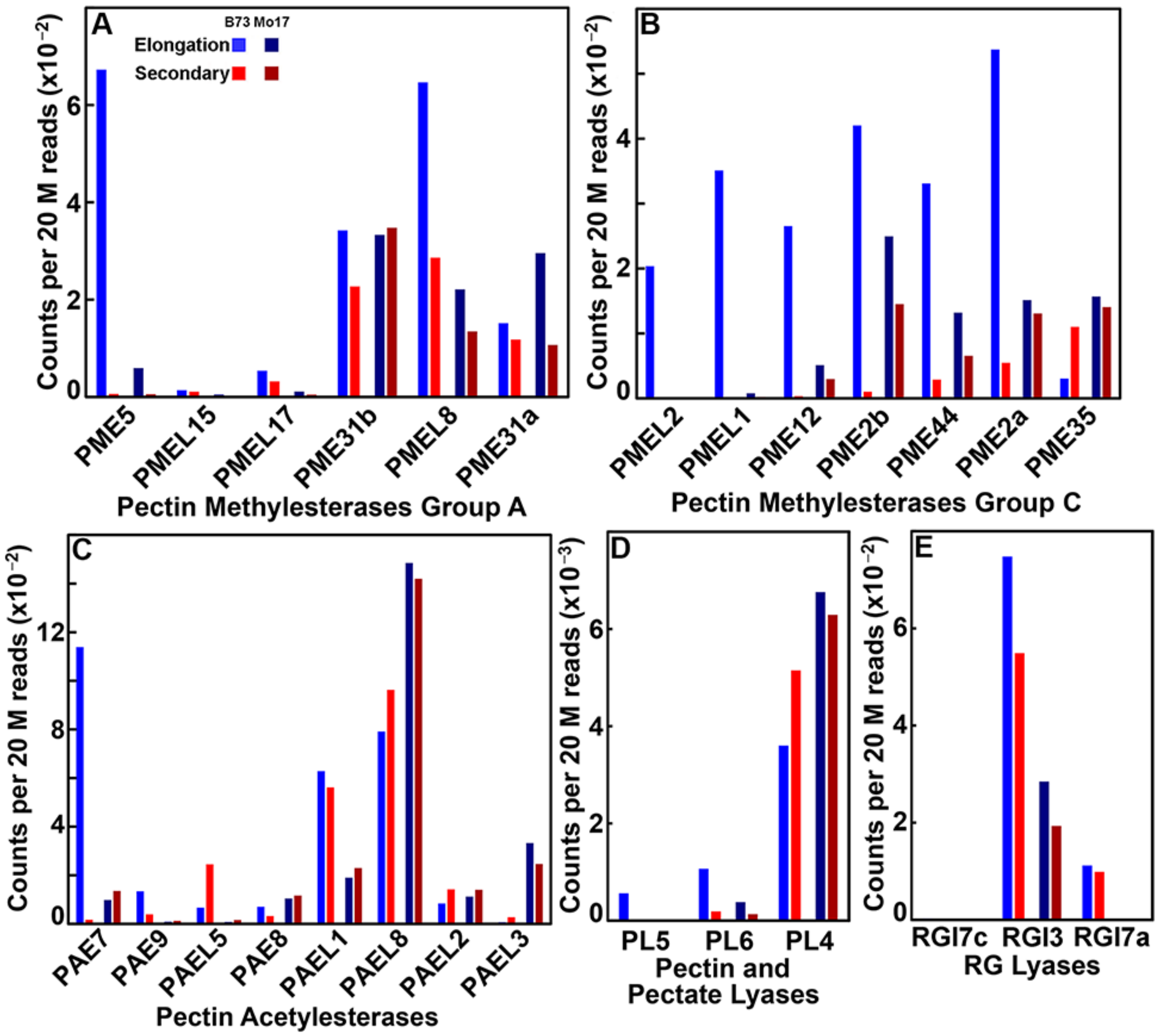


**Figure S43. Comparative expression of maize B73 and Mo17 genes of pectin esterases, and lyases.** Transcript levels were determined as described in the legend of Figure S27. **A.** Pectin methylesterase Group A. **B.** Pectin methylesterase Group C. **C**. Pectin acetylesterase. **D**. Pectin and pectate lyases. **E**. RG lyases.

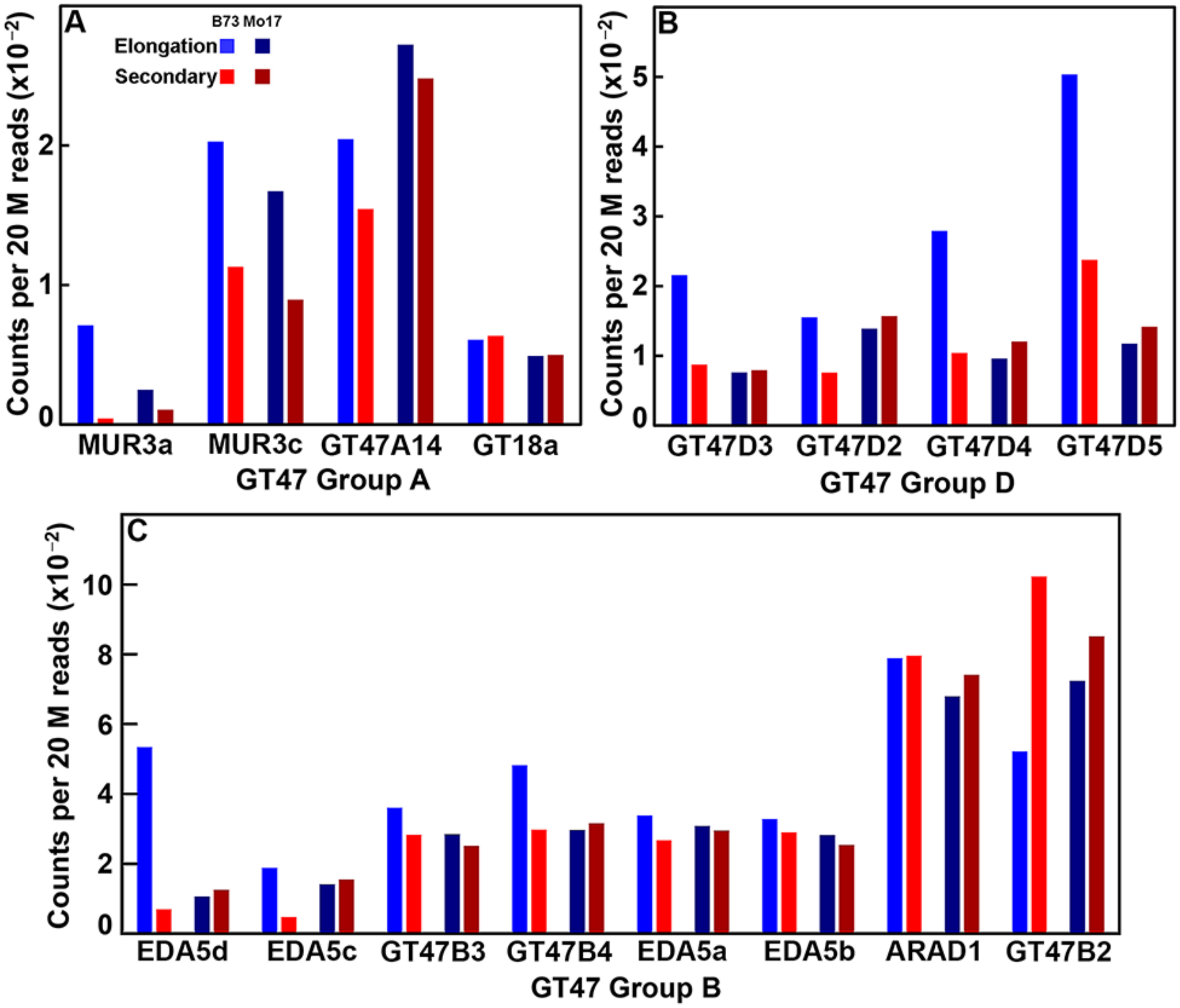


**Figure S44. Comparative expression of maize B73 and Mo17 genes of subgroups of Family 47 inverting glycosyl transferases.** Transcript levels were determined as described in the legend of Figure S27. **A.** Family GT47 Group A. **B.** Family GT47 Group D. **C.** Family GT47 Group B.

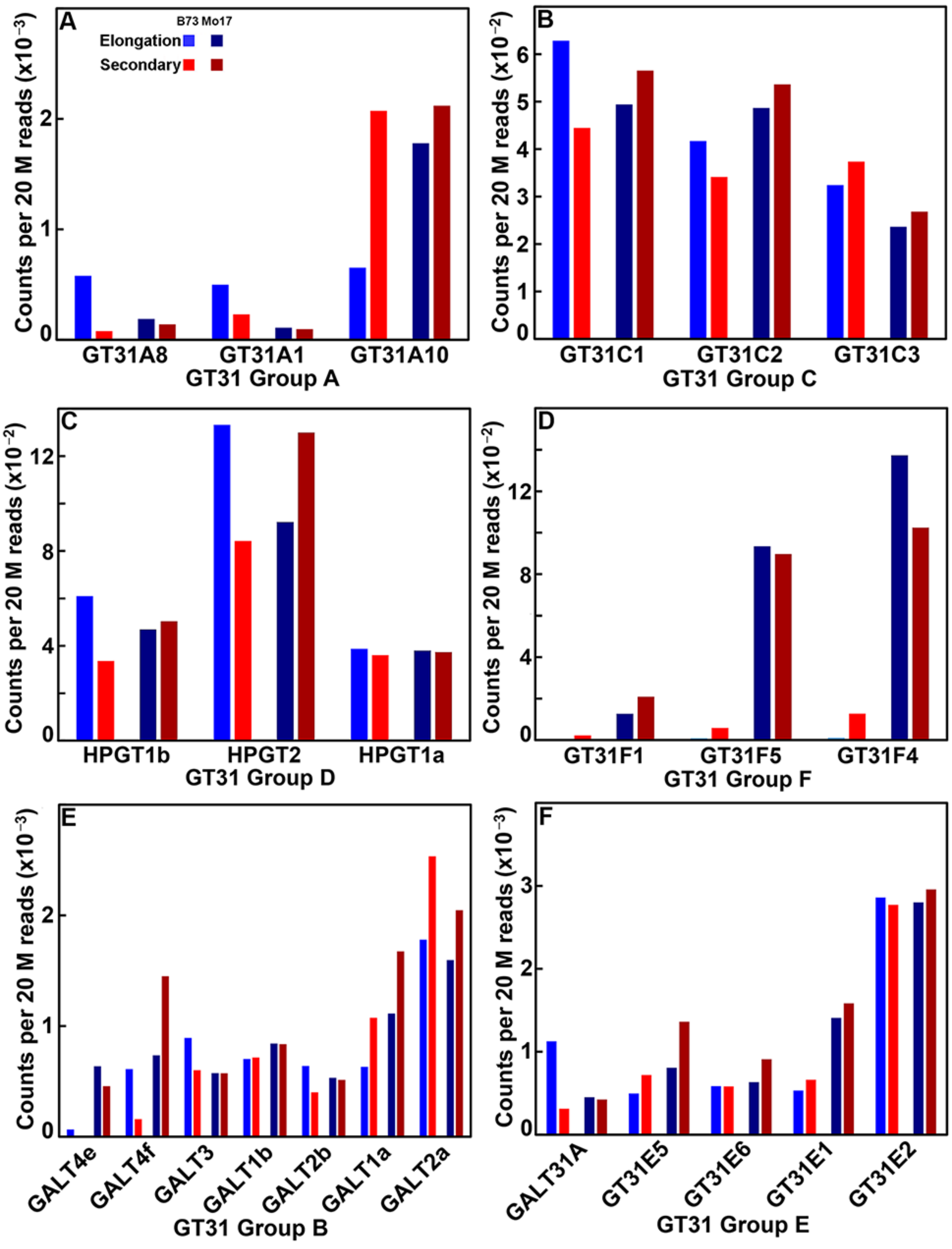


**Figure S45. Comparative expression of maize B73 and Mo17 genes of subgroups of Family GT31 glycosyl transferases.** Transcript levels were determined as described in the legend of Figure S27. **A.** GT31 subgroup A. **B.** Family GT31, subgroup C. **C**. Family GT31, subgroup D. **D.** Family GT31, subgroup F. **E.** Family GT31, subgroup B. **F.** Family GT31, subgroup E.

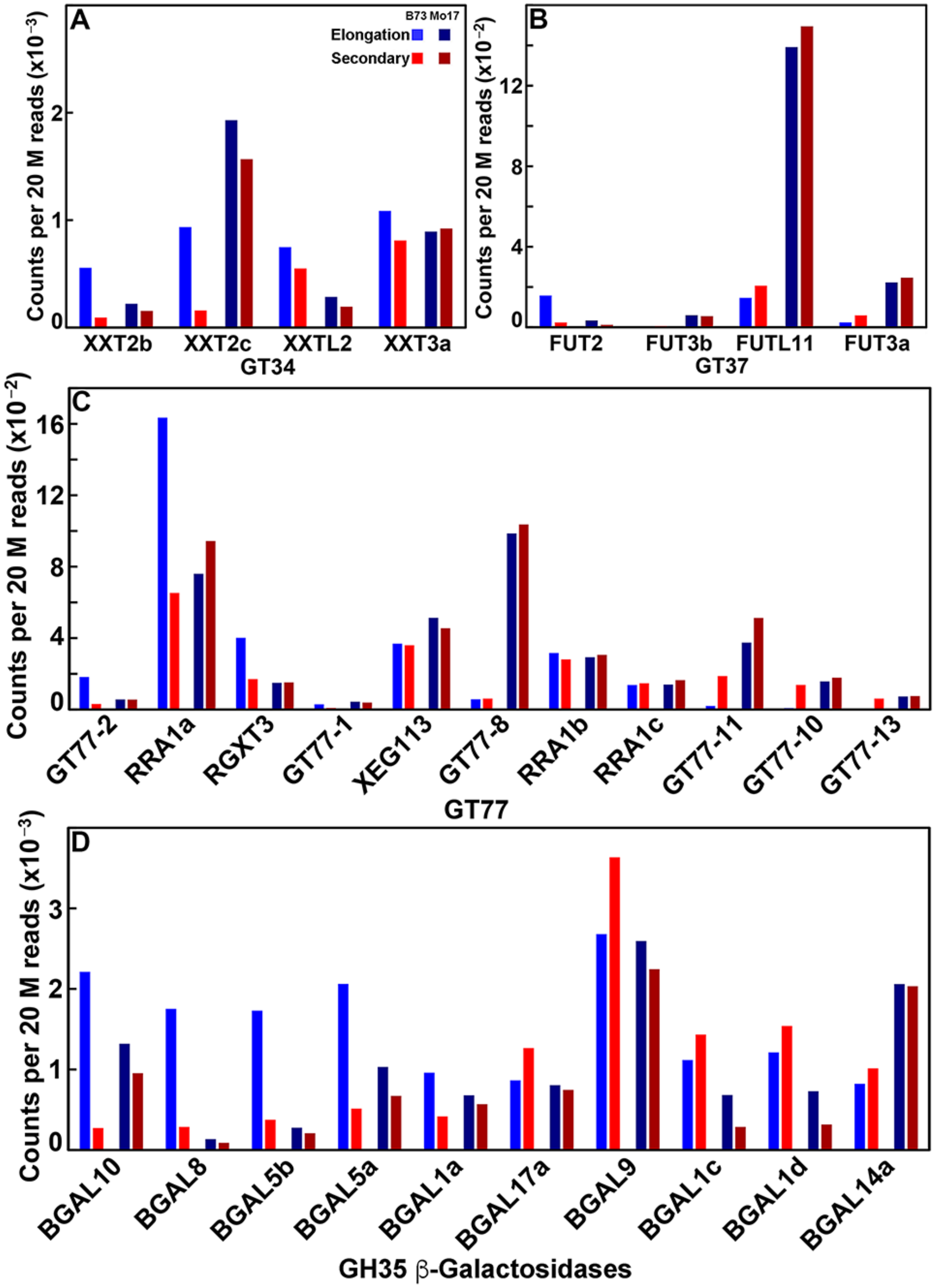


**Figure S46. Comparative expression of maize B73 and Mo17 genes of xyloglucan-related glycosyl transferases, Family GT77 glycosyl transferases, and Family GH35 β-galactosidase.** Transcript levels were determined as described in the legend of Figure S27. **A.** Family GT34, xyloglucan xylosyl transferases (XXTs). **B.** Family GT37, FUT and FUT-like GDP-L-fucose transferases. **C.** Family GT77, α-xylosyltransferases, α-1,3-galactosyltransferases, arabinosyltransferases. **D.** Family GH35, β-galactosidases.

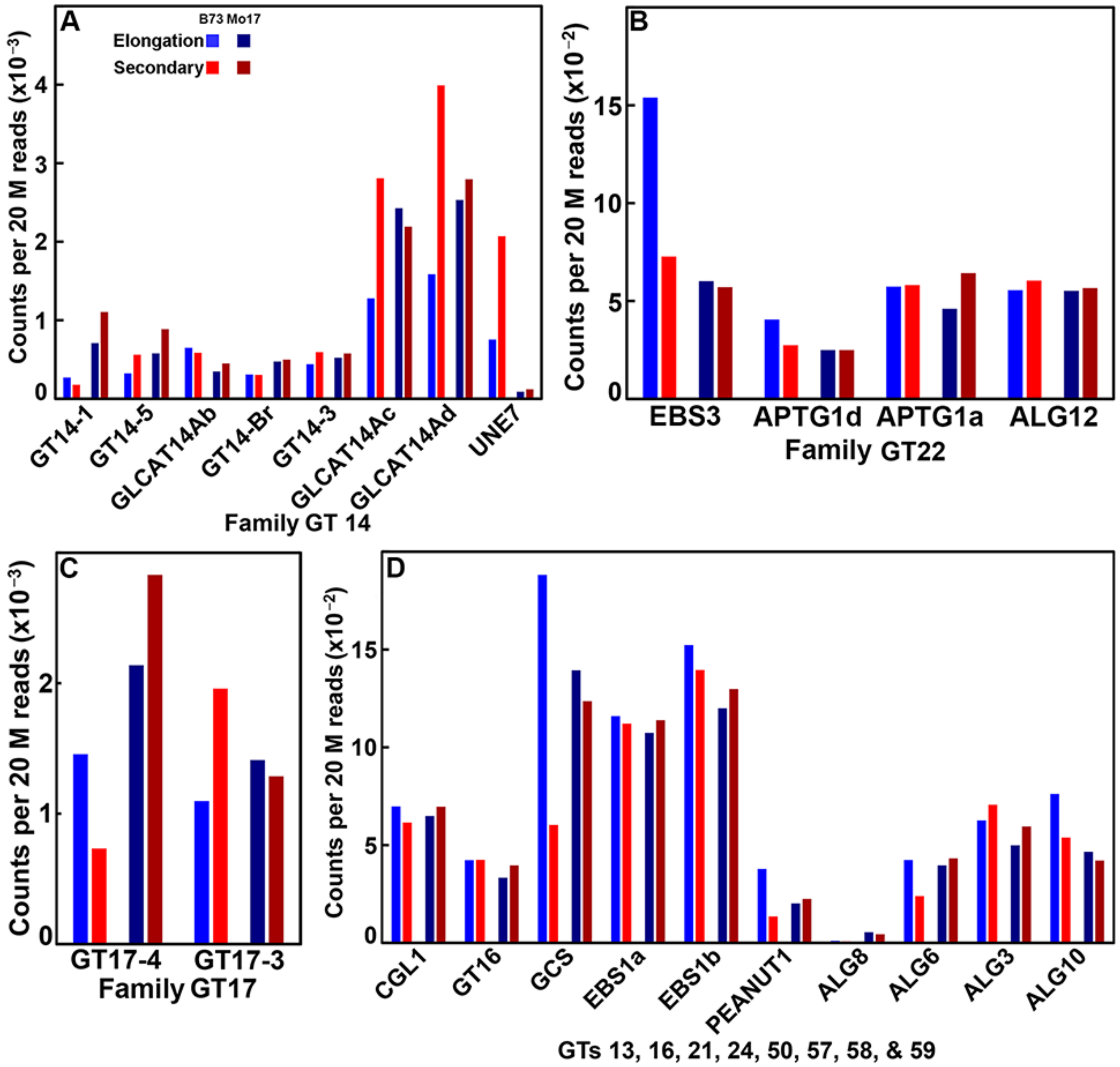


**Figure S47. Comparative expression of maize B73 and Mo17 genes of families of ER-resident glycosyl transferases.** Transcript levels were determined as described in the legend of Figure S27. **A.** Family GT14 β-1,3-galactosyl-*O*-glycosyl-glycoprotein β-1,6-*N*-acetylglucosaminyltransferases and related proteins. **B.** Family GT22 Dol-P-Man: $Man_6GlcNAc_2$-PP-Dol α-1,2-mannosyltransferases and related proteins. **C**. Family GT17 β-1,4-mannosyl-glycoprotein β-1,4-*N*-acetylglucosaminyltransferases and related proteins. **D**. Members of eight additional families of GTs.

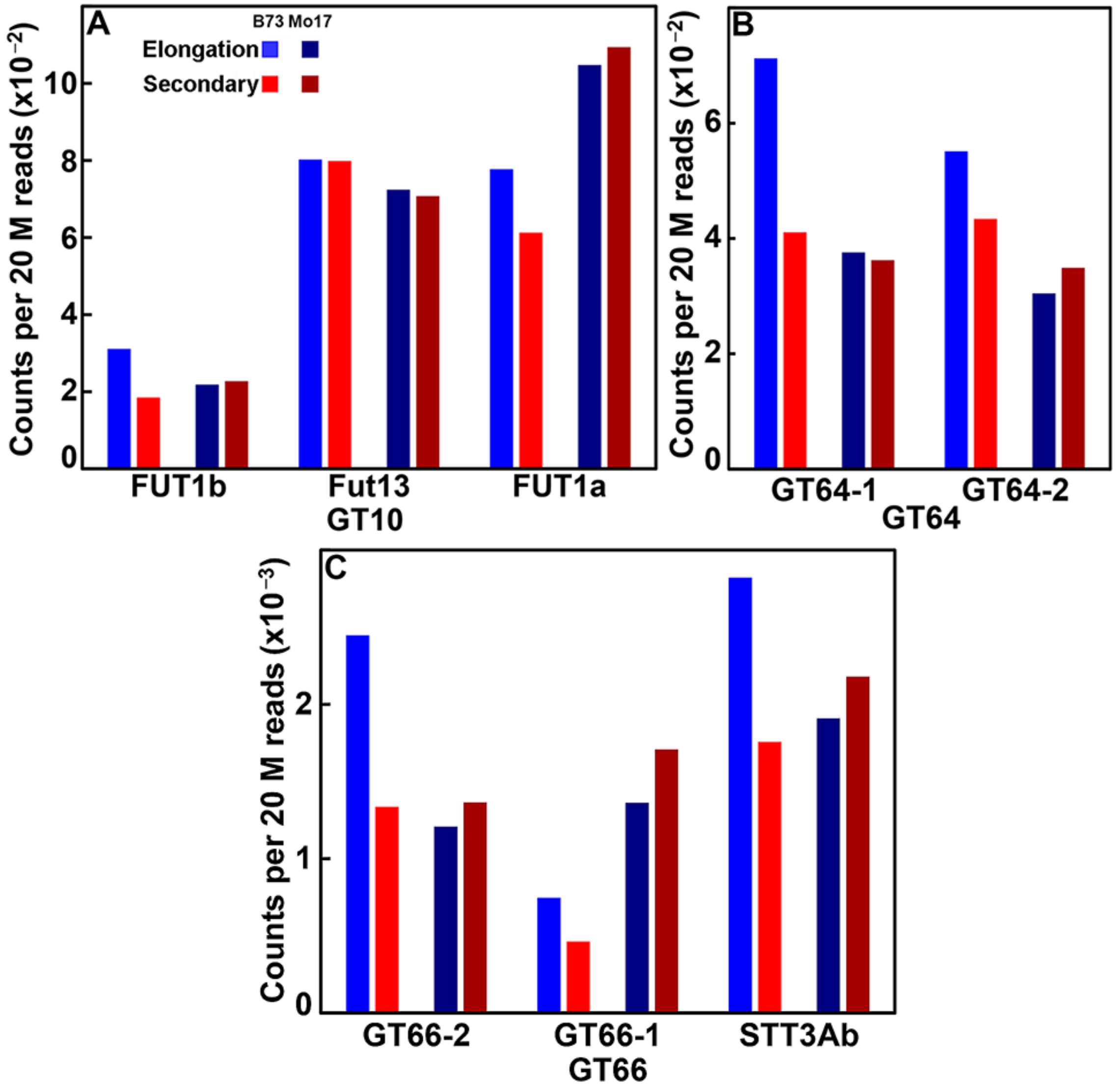


**Figure S48. Comparative expression of maize B73 and Mo17 genes families of Golgi-resident glycosyl transferases.** Transcript levels were determined as described in the legend of Figure S27. **A.** Family GT10, galactoside α-1,3/1,4-L-fucosyltransferases and related proteins. **B.** GT64, heparan α-*N*-acetylhexosaminyl-transferases and related proteins. **C**. Family GT66, dolichyl diphospho-oligosaccharide-protein glycosyltransferases and related proteins.

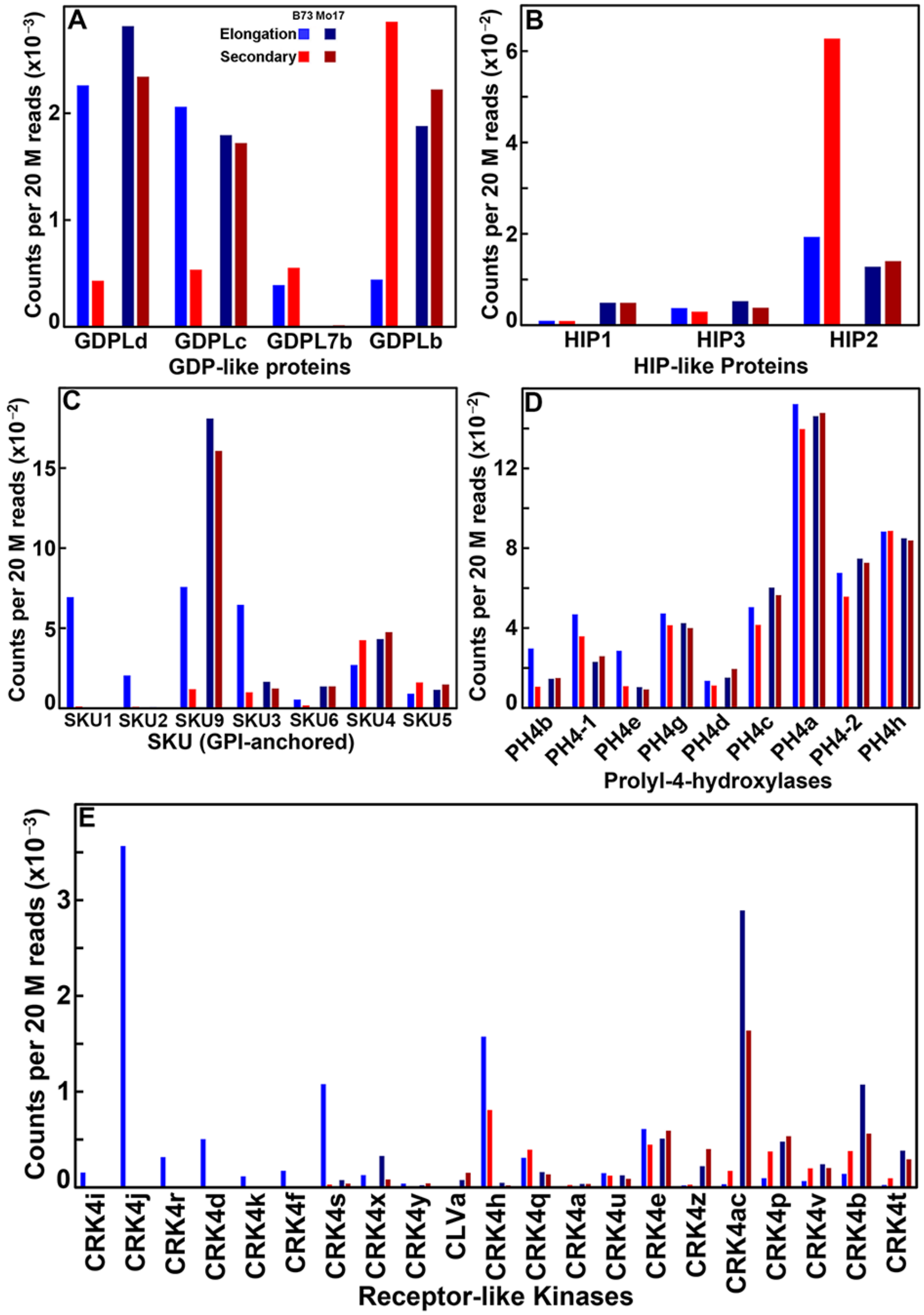


**Figure S49. Comparative expression of maize B73 and Mo17 genes of signaling and secretion-associated proteins.** Transcript levels were determined as described in the legend of Figure S27. **A.** GDP-like guanine nucleotide exchange factors for the α-subunit of the G protein. **B**. HIP-like Hsp70-interacting proteins. **C.** The SKU family. **D.** Prolyl-4-hydroxylases involved in hydroxyproline synthesis. **E**. Receptor-like kinases.

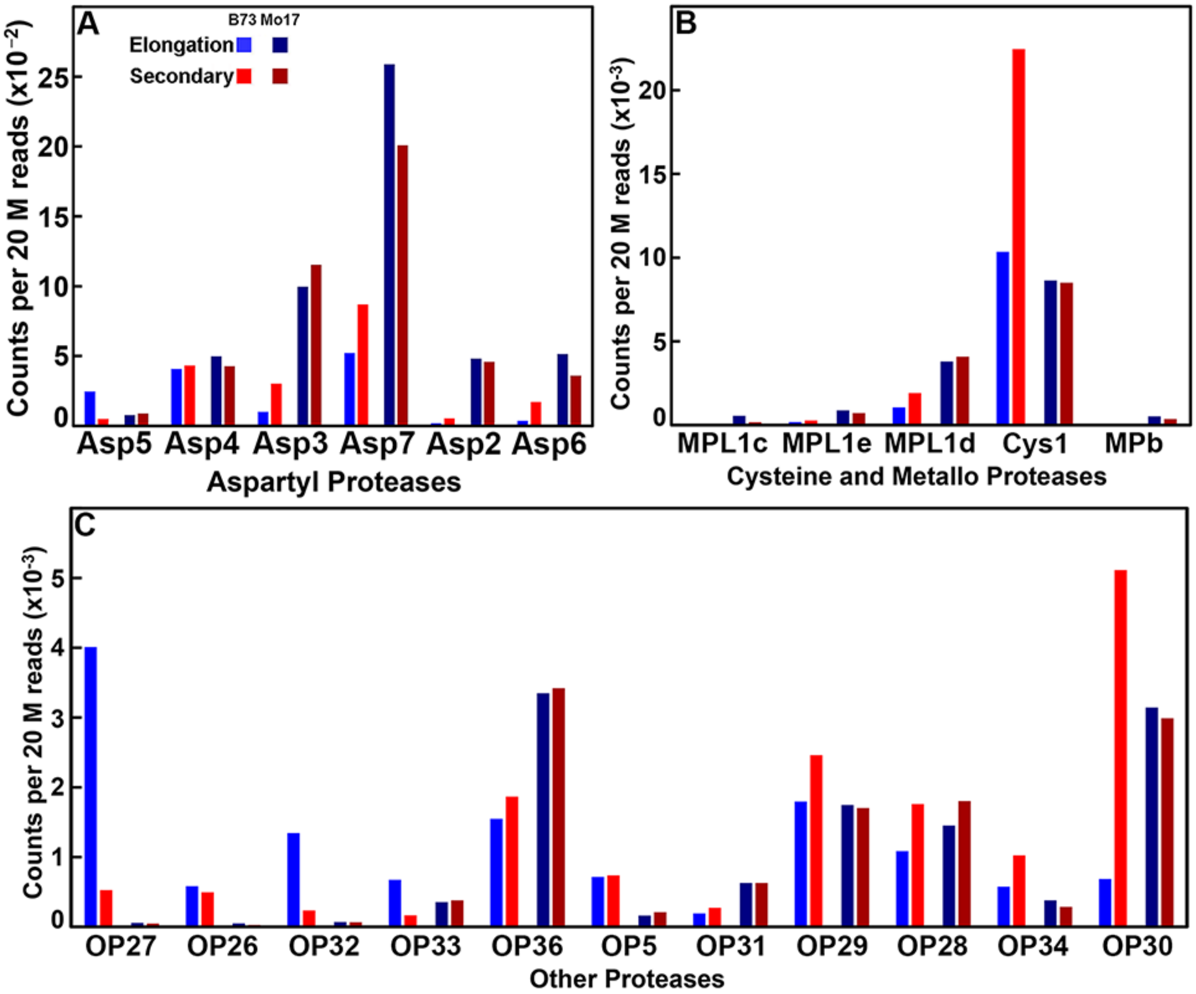


**Figure S50. Comparative expression of maize B73 and Mo17 genes of proteases involved in cell growth and cell wall metabolism.** Transcript levels were determined as described in the legend of Figure S27. **A**. Aspartyl proteases. **B.** Cysteine- and metalloproteases. **C.** Other proteases. [Maize Accession numbers for other proteases are in Additional file 3; Dataset 1]

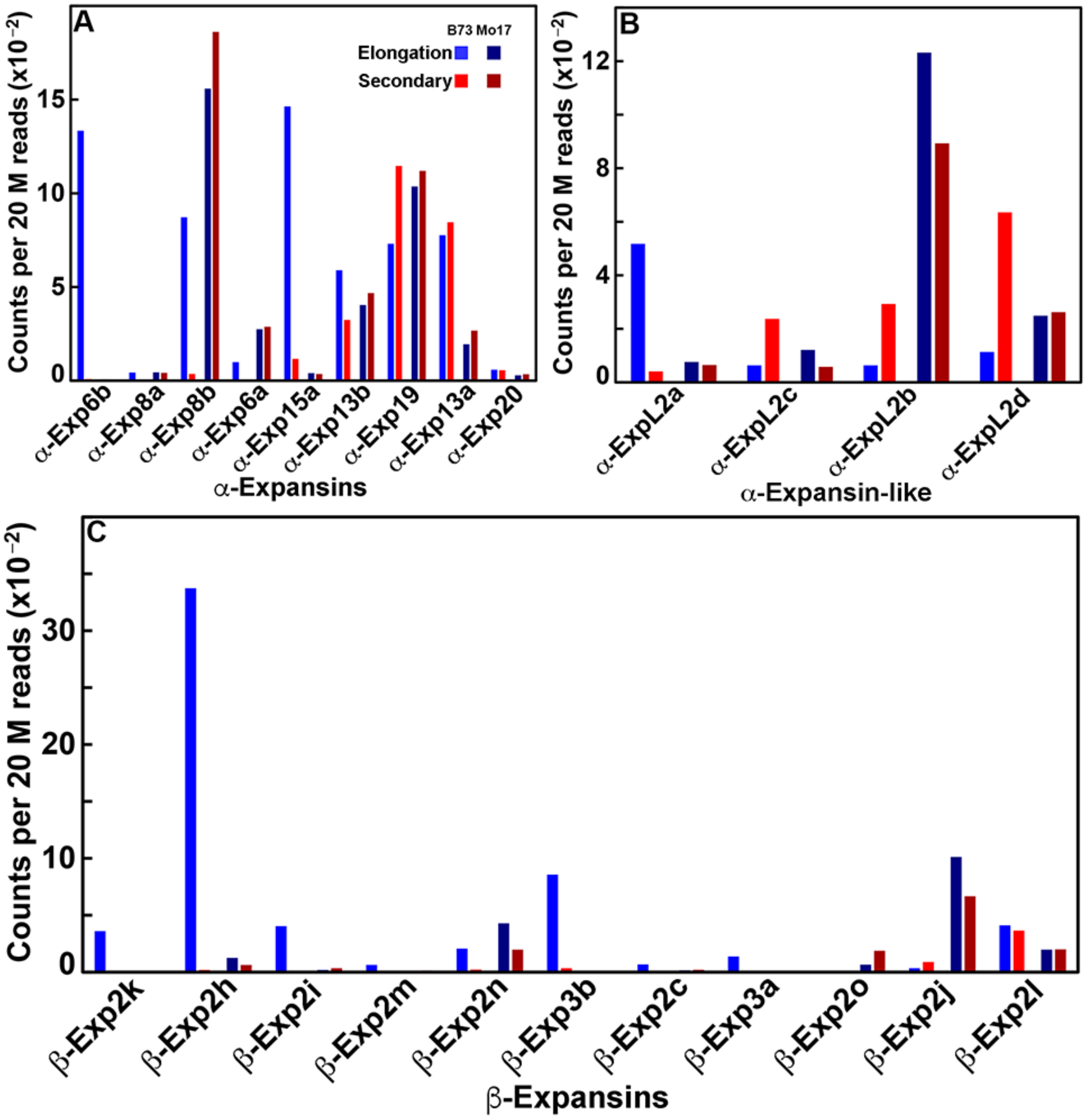


**Figure S51. Comparative expression of maize B73 and Mo17 genes of the expansin superfamily.** Transcript levels were determined as described in the legend of Figure S27. **A.** Family α-Expansins. **B.** Family α-Expansin-like proteins. **C**. Family β-Expansins.

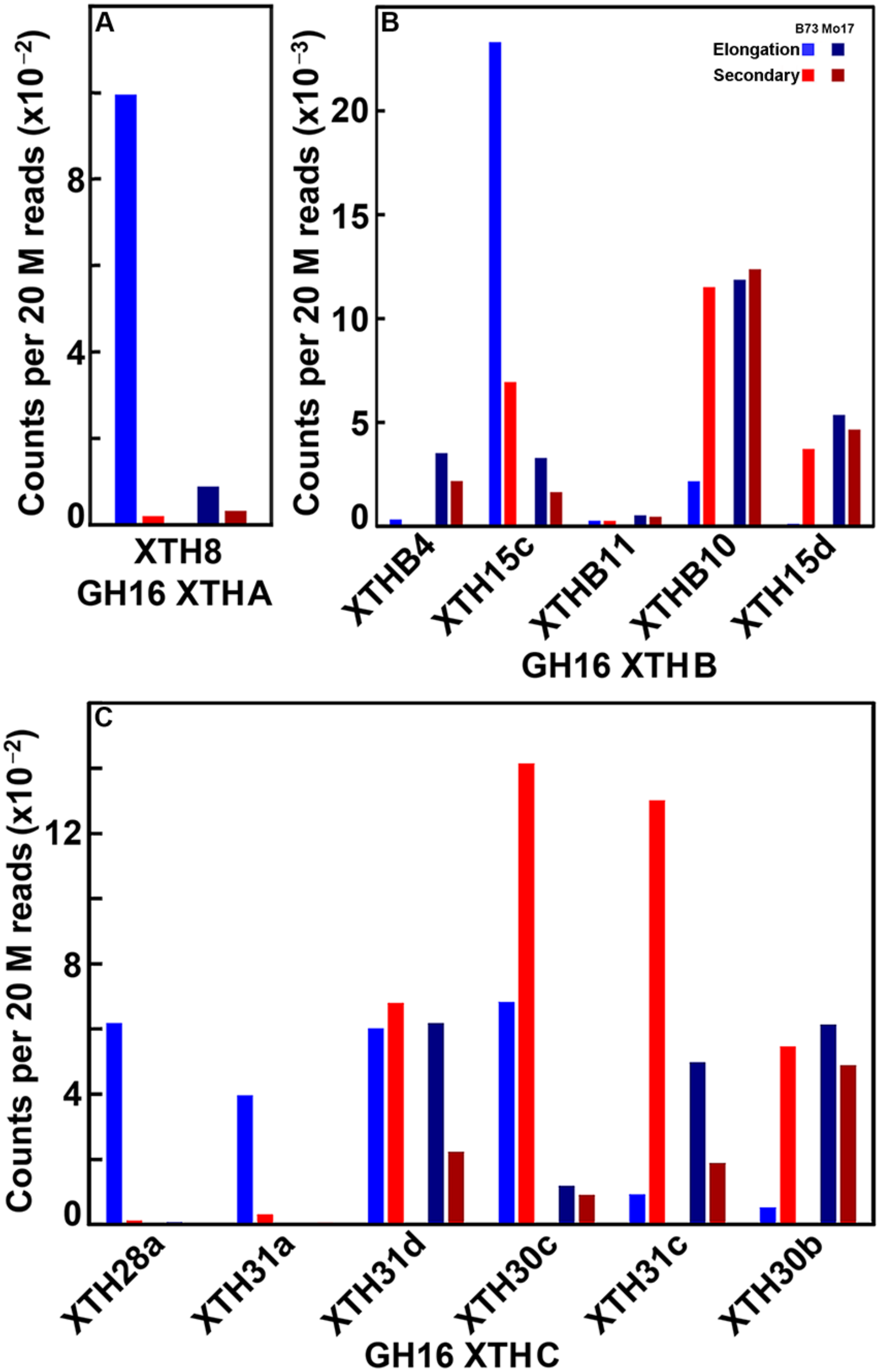


**Figure S52. Comparative expression of maize B73 and Mo17 genes of the GH16 xyloglucan endo-β-D-glucan transferase/hydrolase superfamily.** Transcript levels were determined as described in the legend of Figure S27. **A.** Family GH16 Group A (XTHA). **B.** GH16 Group B (XTHB). **C**. GH16 Group C (XTHC).
