# Supplementary material for: Expression profiles of cell-wall related genes vary broadly between two common maize inbreds during stem development
Source: BMC Genomics. 2019 Oct 29;20:785. doi: 10.1186/s12864-019-6117-z (PMC6819468; doi:10.1186/s12864-019-6117-z)
Supplement: Supplementary file 13 — Additional file 13: Table S4. Fold-change differences in levels of expression of B73 and Mo17 genes in common for both cell wall-related and all genes of elongation and secondary wall stages of stem development. [file 12864_2019_6117_MOESM13_ESM.pdf]

Additional file 13: Table S4. Fold-change differences in levels of expression of B73 and Mo17 genes in common for both cell wall-related and all genes of elongation and secondary wall stages of stem development.[1]

Fold Change	All Expressed Genes (38,264)		Cell Wall Related genes (716)	
	Elongation	Secondary wall	Elongation	Secondary wall
		% of genes		
≥2	59.6	69.9	55.7	44.4
≥5	26.7	33.1	29.7	21.1
≥10	15.4	19.1	20.4	10.5
≥100	1.5	2.0	1.3	0.6

[1]Elongation stage represents cell wall-related genes with ratios of expression of transcript abundance in Internodes 4 and 5 : Internodes 6 and 8 of ≤ 1; Secondary wall genes are those with ratios of expression ≥2.
